# LiLA: lipid lung-based ATLAS built through a comprehensive workflow designed for an accurate lipid annotation

**DOI:** 10.1038/s42003-023-05680-7

**Published:** 2024-01-05

**Authors:** Belén Fernández Requena, Sajid Nadeem, Vineel P. Reddy, Vanessa Naidoo, Joel N. Glasgow, Adrie J. C. Steyn, Coral Barbas, Carolina Gonzalez-Riano

**Affiliations:** 1 Centro de Metabolómica y Bioanálisis (CEMBIO), Facultad de Farmacia, Universidad San Pablo-CEU, CEU Universities, Urbanización Montepríncipe, 28660 Boadilla del Monte, España; 2 Department of Microbiology, University of Alabama at Birmingham, Birmingham, AL USA; 3 Africa Health Research Institute, Durban, South Africa; 4 Centers for AIDS Research and Free Radical Biology, University of Alabama at Birmingham, Birmingham, AL USA

**Keywords:** Lipidomics, Data processing

## Abstract

Accurate lipid annotation is crucial for understanding the role of lipids in health and disease and identifying therapeutic targets. However, annotating the wide variety of lipid species in biological samples remains challenging in untargeted lipidomic studies. In this work, we present a lipid annotation workflow based on LC-MS and MS/MS strategies, the combination of four bioinformatic tools, and a decision tree to support the accurate annotation and semi-quantification of the lipid species present in lung tissue from control mice. The proposed workflow allowed us to generate a lipid lung-based ATLAS (LiLA), which was then employed to unveil the lipidomic signatures of the *Mycobacterium tuberculosis* infection at two different time points for a deeper understanding of the disease progression. This workflow, combined with manual inspection strategies of MS/MS data, can enhance the annotation process for lipidomic studies and guide the generation of sample-specific lipidome maps. LiLA serves as a freely available data resource that can be employed in future studies to address lipidomic alterations in mice lung tissue.

## Abstract (web summary)

An LC-MS and MS/MS-based workflow, integrating 4 software tools and a decision tree, enables accurate annotation and semi-quantification of lipids, providing the Lipid Lung-based ATLAS (LiLA). LiLA then revealed the lipidomic Mtb infection signature.

## Introduction

Accurate annotation of lipid species in lipidomics is essential for advancing our understanding of lipid biology, identifying disease biomarkers, and developing potential therapeutic interventions. It is a foundational step that influences the validity and impact of lipidomics research. The lipid annotation is a multi-step process that begins with the extraction and analysis of lipids from a sample, followed by the characterization and identification of the lipids using different analytical techniques, mainly mass spectrometry (MS). This process also involves the use of software tools to obtain information about the lipid structure and the exact composition of fatty acid chains to increase the biological significance of the lipidome, including chain length and the unsaturation grade, which are essential for the specific role of a biological lipid. A liquid chromatography-mass spectrometry (LC-MS)-based untargeted lipidomics approach has become the most adopted strategy for clinical studies to unveil disease mechanisms and potential biomarkers. However, this strategy renders complex data matrices with thousands of features corresponding to adducts, in-source fragments, neutral losses, multimers, and isotopologues. Despite its utmost importance, the proper annotation of a wide variety of lipid species present in biological samples remains a crucial bottleneck found in untargeted lipidomics studies^1^, mainly due to the complexity of this family of biomolecules and the co-elution of different lipid classes during the chromatographic analysis. Additionally, the experimental conditions have a massive impact on the abundance of characteristic lipid fragments, some of them being common between isobaric and isomeric lipid species^2^ and favouring more than one adduct formation in electrospray ionization^3^, which increases the difficulty of the chromatogram and spectra interpretation. Furthermore, a lipidomics-based analysis usually renders features that do not correspond to unique metabolite compounds, and very frequently, we must deal with the presence of compounds which do not have any biological relevance in our study, and some of them are even unknown^4^. These compounds could interfere with those lipids that, in fact, are of great significance in our research, resulting in a very time-consuming process during data analysis in our studies^4,5^. In recent years, efforts have been combined to develop multiple lipid annotation tools to alleviate all these issues, increasing the number of mass spectral libraries^6^, however, this is insufficient. For these reasons came up the necessity of optimizing the lipid annotation process to increase the number of lipid species accurately annotated, with greater structural details and a higher degree of confidence.

Lipidomics studies can be conducted using either a targeted or an untargeted approach. Typically, targeted analyses aim to identify and quantify a limited number of lipids. However, obtaining all the required chemical standards for the lipids of interest can be difficult, if possible, resulting in limited coverage of detected lipid species. Conversely, the untargeted lipidomics approach focuses on simultaneously detecting as many compounds as possible, providing a wide range of lipids/features with various chemical and physical properties. Still, identifying and quantifying all detected lipids/features remains a challenge. The primary hurdle is maximizing the detection and accurate identification of thousands of metabolites while maintaining a decent detection dynamic range and quantification capability^7,8^. In our case, we were committed to implementing a different analytical strategy from the data processing point of view.

To achieve this goal, we performed an untargeted lipidomics analysis using lung tissue of control mice (*Mtb*^−^) by an RP-UHPLC-ESI-QTOF MS, followed by a data-dependent analysis (DDA) adopting an iterative-MS/MS strategy attempting to obtain the fragmentation data from the underlying features. Then, we established an annotation workflow to build an accurate database containing high-quality lipid annotations using the obtained DDA data. We relied on the use of four annotation software programs based on three different and complementary annotation approaches, including the spectra matching (Lipid Annotator^9^, MS-Dial^10^), the bottom-up strategy (LipidHunter^11^) and the fragments intensity rules (LipidMS^12^)^13^. Then, we generated a database with the tentative lipid annotations by combining all the information collected from the four software programs. Next, we introduced a decision tree to curating the tentative database by establishing different criteria to decide whether the tentative annotation was accurate or not, including the manual inspection of the MS and MS/MS spectra to reconstruct the parent–fragment ion relationships and verify the lipid annotations based on structural elucidation rules. Therefore, the decision tree allowed us to curate the initial database, validate the accuracy of the annotation, and subtract the false positives that could interfere with our studies’ outcomes. At the end of the process, we obtained a database containing 866 accurately annotated lipid molecular species.

The development of an ATLAS of lipids, which characterizes the quantitative distribution and relationship of lipids in various biological samples, is indeed valuable for understanding the metabolome and lipidome composition. By creating such an ATLAS, researchers can compare individual studies with animal models and human population health data, facilitating a comprehensive analysis of lipid profiles. Due to the scarcity of a detailed quantitative inventory of tissue lipidomes, we generated a Lipid Lung-based ATLAS (LiLA) by performing the semi-quantification of the lipid species included in the previously developed mice lung reference database. As a result, 709 lipid species were semi-quantified using the SPLASH® Lipidomix® internal standard mixture, providing a comprehensive and multi-dimensional analysis of lipids. Finally, LiLA was used to reprocess untargeted data acquired from *Mtb*-infected mice lung samples collected at two-time points, four- and twelve-weeks post-infection (*Mtb*^*+*^*4w*, *Mtb*^*+*^*12w*), to unveil the lipid dysregulation induced by Tuberculosis (TB) and its progression. The strategy of using an ATLAS containing hundreds of lipid species to reprocess untargeted data is what we named a hybrid lipidomics approach.

LiLA exemplifies a data-rich resource that is freely available and will sustain further understanding of lipidomic alterations within mice lung tissue. Furthermore, our decision tree-based proposal can assist the annotation process for any lipidomics-based study and could act as a guideline to generate other sample-specific lipidome maps.

## Results and discussion

### Setting up and optimizing the LC-MS/MS parameters is critical for the lipid annotation process

The optimization related to the analytical procedure, including the sample preparation, the chromatographic conditions, and the ionization efficiency are critical to the compound annotation process^4^. Data-dependent Analysis (DDA)^14,15^ is one of the most frequently applied data acquisition strategies, using user-optimized parameters, which is central to ensuring high spectral quality, coverage and the number of compound annotations, providing the most comprehensive molecular insights into a sample^16,17^. We performed the DDA analysis at two fixed collision energies (CE), 20 and 40 eV since it has been demonstrated that combining spectra acquired with different CE may provide more structural information and, therefore, better reference mass spectra for lipid annotation^18^. Additionally, we selected a small isolation width window (1.3 *m/z*) for the quadrupole to isolate the precursor ion, leading to higher MS/MS spectral quality and increasing the sensitivity, isotope coverage, and annotation rates. The MS and MS/MS *m/z* range was also optimized for the data acquisition. The MS detection range was set between 100 and 1700 *m/z* to prevent noise from being introduced in the analysis. In contrast, the range for MS/MS analysis was increased to 40–1700 *m/z* to detect potential characteristic fragment ions with low *m/z* values, such as the diagnostic fragment ions of acyl-carnitines (*m/z* 85.0289 and 60.0798)^19^. This approach allowed us to achieve a more comprehensive analysis while ensuring accuracy and minimizing noise. To improve MS/MS coverage, we set the number of MS peaks for subsequent fragmentation and MS/MS scans to three per duty cycle. We employed the Iterative-MS/MS mode, which selects the top three precursor ions based on intensity in each run. By doing so, we aimed to enhance the analysis of the sample by focusing on the most intense peaks, ultimately leading to better MS/MS coverage. The precursor ion threshold was also optimized, looking to a compromise between increasing the MS/MS coverage of the lipid species present in the sample at lower abundances but preventing the fragmentation of the background noise. An additional critical parameter to be considered when configuring the DDA parameters is the exclusion time. After an MS peak was selected for fragmentation, we established a 0.1-min interval to allow the algorithm to select it again if it remained the most intense peak. Finally, we created an exclusion list that included reference masses and potential contaminants detected in the blank samples. In this way, only the compounds from our sample were selected for fragmentation during the DDA analysis, while external contaminants were excluded.

**Fig. 1:**
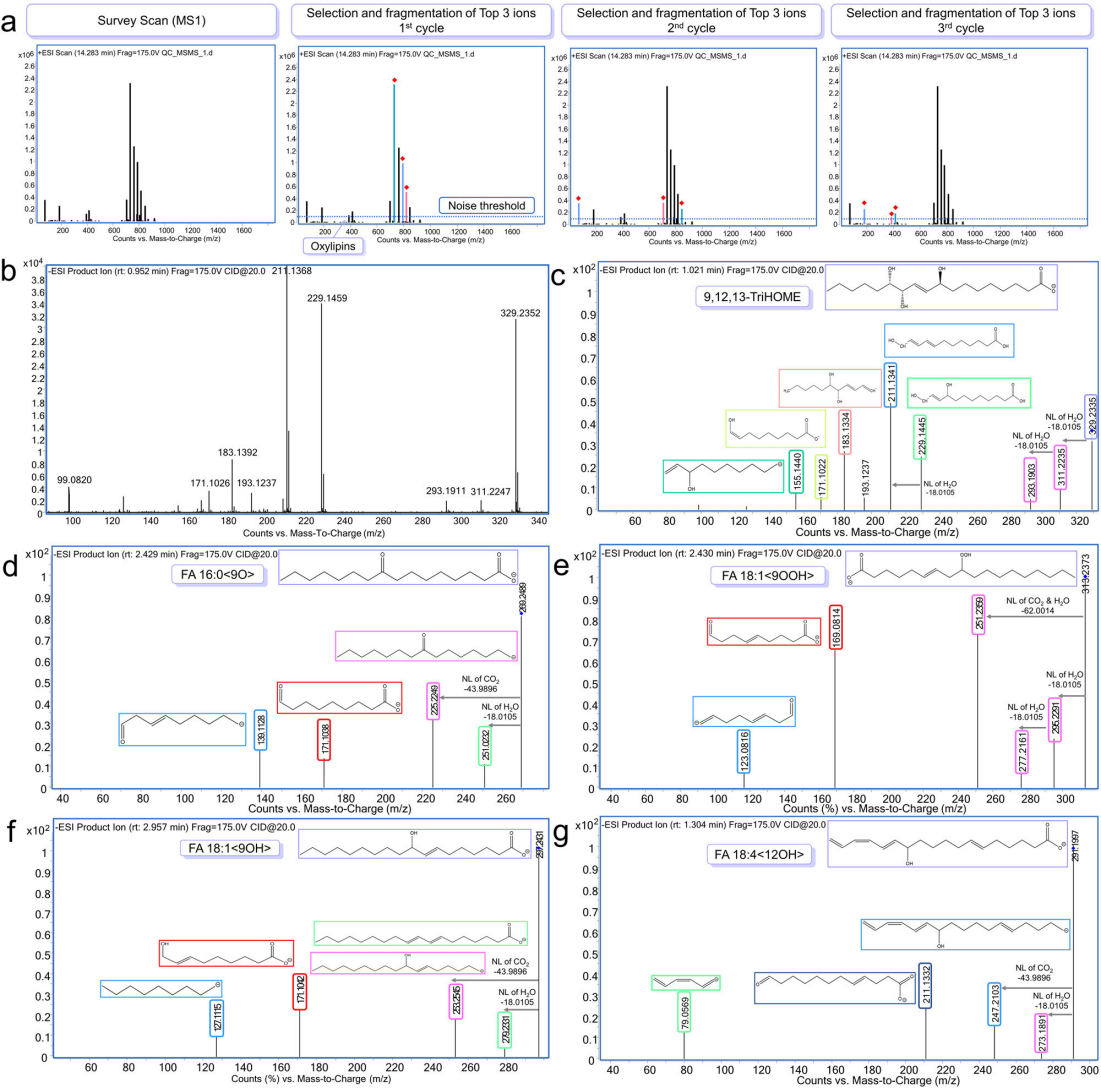
The manual inspection process allowed us to detect 5 oxylipins. **a** Graphical representation of the DDA-MS analysis. **b** ESI(–)-MS/MS spectrum of 9,12,13-TriHOME commercial standard (Cayman Chemical, MI, USA). **c** ESI(–)-MS/MS of 9,12,13-TriHOME detected in the mice lung sample displaying the same RT and fragmentation pattern as the commercial standard. **d** ESI(–)-MS/MS spectrum of FA 16:0 <9O> or 9-keto palmitic acid. **e** ESI(–)-MS/MS spectrum of FA 18:1 <9OOH>. **f** ESI(–)-MS/MS spectrum of FA 18:1 <9OH>. **g** ESI(–)-MS/MS spectrum of FA 18:4 <12OH>.

However, DDA is a process driven by an automated instrument which decides on the fly which are the top precursors and then fragments them one after the other, introducing a certain level of bias. Moreover, one of the DDA drawbacks is that sometimes the algorithm leaves lower abundant signals unanalyzed and therefore unidentified in the experiment^20^. Oxylipins, as bioactive lipid mediators, play a crucial role in numerous physiological processes, including blood flow regulation and inflammation. Nevertheless, the analysis of oxylipins poses important challenges, primarily due to their limited presence in samples and structural diversity^21^. Consequently, our study’s software tools have failed to annotate this class of compounds due to their low biological abundance, since most of them fell below the noise threshold set in the DDA analysis. Thus far, manual data inspection has allowed us to annotate five oxylipins, with only one having a commercially available standard, which allowed us to reach a confidence level 1 for this lipid species. Unfortunately, we have been unable to ascertain a higher level of structural detail for the remaining compounds, primarily due to the inherent challenge of determining the precise positions of the double bonds within their structures (Fig. 1a–g).

### Comprehensive workflow for an accurate lipid annotation using MS/MS data

**Fig. 2:**
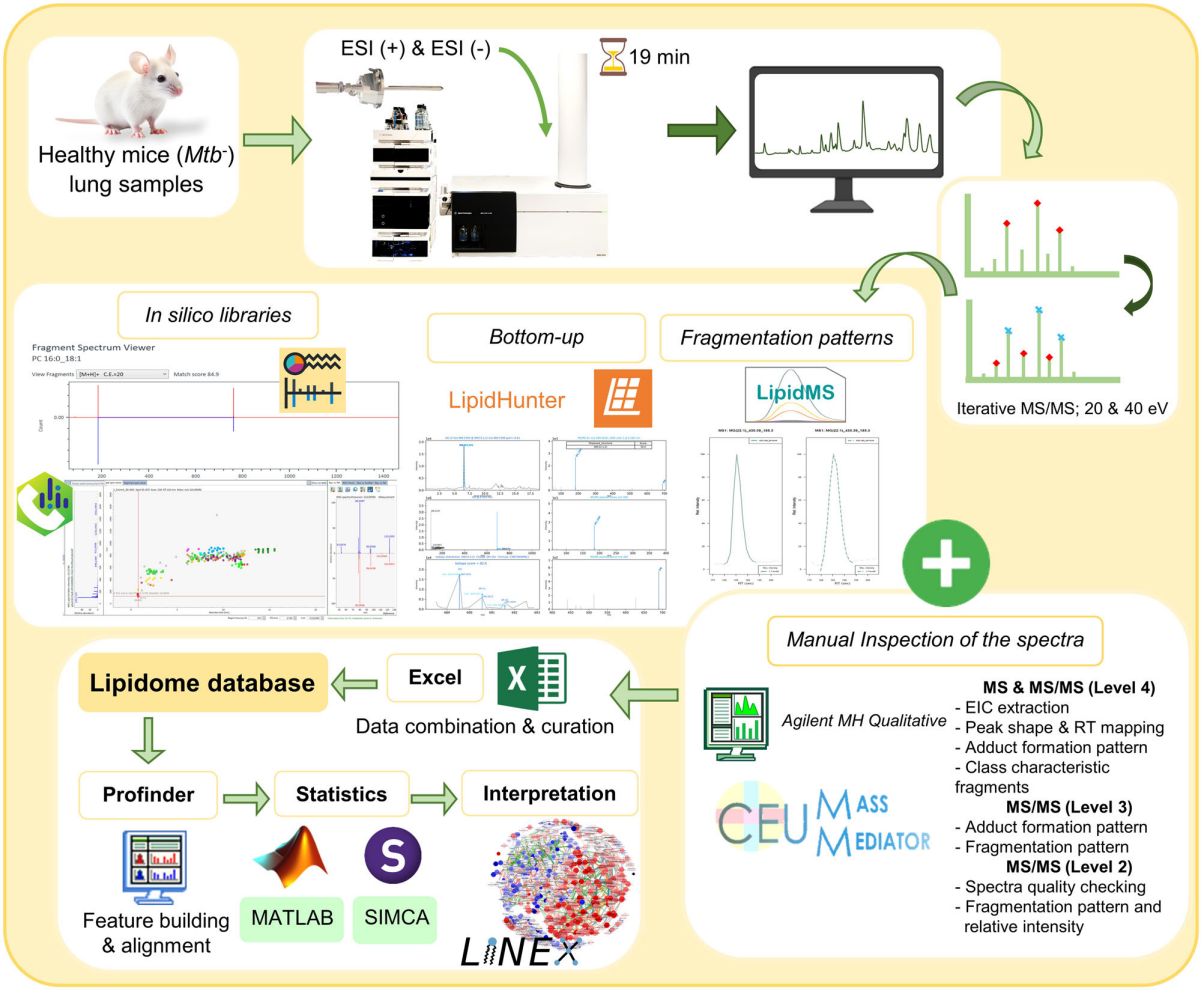
Schematic workflow followed in this study. The lipid annotation was made by using four different software, together with the manual inspection of the spectra. Data curation was performed using a decision tree to generate a lung lipidome database of healthy mice lung tissue. Finally, the usefulness of LiLA was tested by using it to unveil the lipid alterations induced by the *Mtb* in the mice lung samples, by using Profinder software for feature building and time alignment, MATLAB and SIMCA for statistical analysis, and LINEX for pathway analysis.

A thorough description of the lipidomic profile of a specific biological matrix sample can provide valuable insights into the diverse range of lipid classes and subclasses present in the matrix. This information could serve as a reference for future studies and aid in identifying lipid biomarkers or potential therapeutic targets^22–24^. However, the proper annotation/identification of metabolites and lipids present in biological samples is a big issue that has not yet had a unique and standardized solution. However, to date, hundreds of scientists worldwide are doing their best to find and combine different annotation tools to increase the annotation confidence level and the number of compounds found in biological samples under study. As part of this community, we tried to enhance the annotation process to overcome the obstacles that might appear during the performance of this arduous task. Here, using the iterative MS/MS data files obtained from lung samples of healthy control mice (*Mtb*^−^) in both positive (ESI+) and negative (ESI-) MS ionization modes, we selected four different annotation software programs based on three different annotation strategies and combined the information obtained from them to increase the lung lipidome coverage (Fig. 2).

Lipid Annotator v1.0 (Agilent) and MS-DIAL 4 (Riken) tools are based on the MS/MS annotation by spectral matching using in silico libraries included in the software. The in-silico library included in Lipid Annotator is a modified version of LipidBlast database. This software uses an algorithm that combines probability density, Bayesian scoring, and a non-negative least square fit to search a theoretical lipid library. The software allows the deconvolution of co-eluted lipid isomers using the Bayesian probability, determines the relative abundance of those isomers using non-negative least squares fit, and proposes a possible annotation for the dominant lipid species^9^. The Mass Spectrometry-Data Independent Analysis software (MS-DIAL) is a data-processing pipeline for large-scale untargeted metabolomics applicable to either data-independent or precursor-dependent MS/MS fragmentation methods. It was created as a universal program for untargeted metabolomics that supports multiple instruments and MS vendors. It contains an enriched LipidBlast library version and allows the independent spectral deconvolution of the data and, as a result, the software proposes a possible precursor ion for each detected peak^10^. The LipidHunter software tool performs the lipid annotation process based on a bottom-up strategy using a predefined fatty acids checklist. This program is an open-access computational tool for the high-throughput identification and characterization of lipids in mass spectrometry DDA data. It uses machine learning algorithms to analyse the mass spectra of lipids and predict the identities based on their mass and fragmentation patterns. The software tool matches product and neutral loss signals obtained by LC-MS/MS to a user-defined white list of fatty acid residues and lipid class-specific fragments^11^. The fourth software employed in this study was LipidMS 3.0. This tool, launched in 2022, performs the lipid annotation based on fragmentation rules and fragments intensity. LipidMS 3.0 is a software package for R, also implemented as a web-based application, that provides a set of functions for lipid annotation and includes MS data pre-processing options, including peak detection and peak alignment^12^. Since it is built on the R-environment, it is compatible with the mzR, XCMS and CAMERA R-packages. It is also integrated with LipidSearch, providing an easy-to-use interface, allowing users to search for putative lipid identifications and evaluate the quality of the results.

To date, no study has been published that utilizes the combination of these four software tools for lipid annotation, possibly due to the recent development of some of these tools, making them unavailable for previous studies. Moreover, learning how to use these tools and processing the data matrices generated from them can be time-consuming, further contributing to the lack of previous research on this topic. A recent review of bioinformatics tools for MS-based lipidomics analysis revealed over 30 open-access software tools for lipidomics data processing and annotation^25^. MS-Dial, for instance, is one of the most used software tools due to its versatility and free access, making it widely adopted in the lipidomics field^25–27^. Some annotation tools, such as Lipid Annotator, are not freely accessible, limiting their usage compared to other more widely available tools. Although most software provides information about retention time (RT), mass-to-charge ratio (*m/z*), adducts, ionization mode, total lipid composition, and unsaturation grade, only LipidHunter and Lipid Annotator provide a score for annotation confidence level and neutral molecular formula. Meanwhile, all software programs except LipidMS show the fragmentation pattern of each feature and the corresponding matching compound. This information is valuable for verifying the presence of characteristic lipid class fragments and ensuring annotation accuracy. Furthermore, in some cases, the software’s fragmentation pattern can help to identify and discard false-positive metabolites based on mismatched ion fragments.

### A decision tree to curate the massive amount of information obtained from the different software tools

**Fig. 3:**
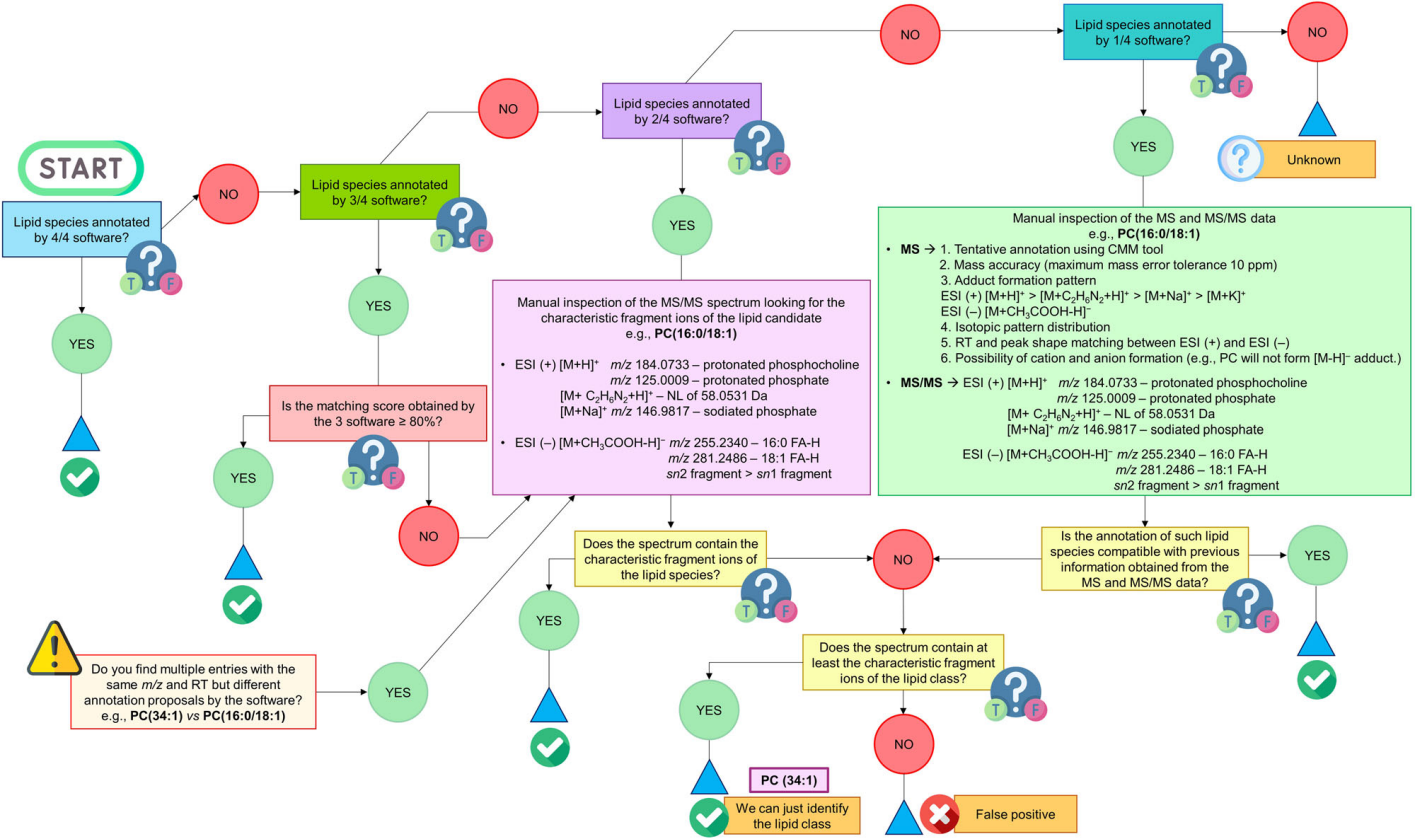
The designed decision tree helped us to curate the initial massive amount of information obtained by the four software tools. The decision tree was designed to increase confidence in the lipid species annotations and remove the false positives. This decision tree consisted of four main steps that properly guided us to perform an accurate lipid annotation based on quality criteria. The number of software tools that a particular lipid species is annotated by dictates the path to follow through the different steps within the decision tree. The confidence level of the annotation and its precision, which means if the software could adequately determine the chain length, the unsaturation grade and position within the lipid structure, were also considered.

To manage the large volume of data produced by the four annotation tools, we combined all CSV files into a single Excel file, which contained all the information provided by the four software programs relative to each annotated lipid, including the RT, *m/z*, abundance, lipid class, sum composition, the complete compound name where the chain length and unsaturation grade were indicated (if possible), the ion species and the formula. Considering all the information, we established a decision tree based on specific exclusion criteria to handle the overwhelming data produced by the annotation tools, avoid false positive annotations, and provide a comprehensive lipid profile of the mice’s lung tissue samples (Fig. 3 and Supplementary Fig. 1). We divided the decision tree into 5 main steps described below:

1. Lipid species annotated by all four software programs: We concluded that the lipid was accurately annotated if we found four entries within the raw database with the same structural annotation, *m/z*, and RT. Additionally, we considered the adduct pattern that each lipid class could exhibit. In instances where the same lipid species appeared with the same structural annotation and RT but different adducts and, consequently, *m/z*, we reviewed the adduct pattern that the lipid class could present and selected the adduct form that rendered the most intense signal^3^.
2. Lipid species annotated by three out of four software programs: When three of the four software programs displayed the same structural annotation, *m/z*, and RT, we evaluated the matching score provided by each software. If the matching score was above 80% in all three software programs, we determined that the lipid had been accurately annotated. However, if the matching score was below 80%, we conducted a manual review of the MS/MS spectra to identify the lipid’s characteristic fragment ions. After the inspection, we concluded that the lipid was accurately annotated if the MS/MS spectrum contained the characteristic fragment ions. If not, we attempted to provide a composite annotation by examining if the spectrum contained at least the characteristic fragment ions of the lipid class (Supplementary Fig. 1). If previous information was not confirmed, it was considered a false positive, and we tried to accurately annotate it relying on structural elucidation rules, focusing on the specific fragmentation pattern of each lipid class. In this step, the adduct pattern that each lipid class could exhibit was also considered for those cases where a particular structural annotation appeared several times at the same RT but with different *m/z*.
3. Lipid species annotated by two out of four software programs: We employed a conservative approach when two entries with the same structural annotation, *m/z* and RT, were detected in our raw database and conducted a manual inspection of the MS/MS spectrum as previously detailed. Following this strategy, we could determine if the structural annotations were accurate. If the annotation was incorrect, we attempted to provide a more general annotation of the lipid class (sum composition) or determine if the software programs had provided a false positive annotation. In those cases where the annotation was incorrect and a false positive, we made every effort to manually annotate the lipid species accurately.
4. Lipid species annotated by one of four software programs: Maintaining a conservative approach, we manually inspected the MS and MS/MS data when a lipid species was only annotated by one software. Regarding the MS data, an initial search of the *m/z* using the online tool CEU Mass Mediator (CMM) (http://ceumass.eps.uspceu.es/mediator/)^28^ was performed to provide a tentative annotation of the lipid class. The tentative assignment was performed based on (i) mass accuracy (maximum mass error tolerance 10 ppm); (ii) adduct formation pattern; (iii) isotopic pattern distribution; (iv) RT and peak shape matching between ESI (+) and ESI (–); (v) possibility of cation and anion formation. The MS/MS data were then inspected to verify the structural annotation of the lipid species. After collecting all the information provided by the MS and MS/MS data, we had three different scenarios. First, the annotation of such lipid species was compatible with previous information obtained from the MS and MS/MS data; therefore, the annotation was accurate. If not, we checked if the lipid class could be confirmed and provided the sum composition annotation type. In the final scenario, where the MS and MS/MS data was not compatible with either the lipid species or class, it was catalogued as a false positive, and a manual annotation was attempted.
5. Sum composition *vs* complete compound name: When we encountered multiple entries with the same RT and *m/z* but differing structural annotations, we conducted a manual review of the MS/MS spectra to confirm the structural composition (e.g., PC(34:0) *vs* PC(18:0/16:0)).
6. Unknown features are prevalent in untargeted lipidomics data, and its annotation/identification remains an important bottleneck in the field. As demonstrated in this study, modern spectral libraries and user-friendly bioinformatic tools can help annotate hundreds to thousands of lipids of biological interest. However, even with the best available spectral search tools, more lipid features typically remain unidentified than identified^29^. Our strategy for the features classified as unknowns was the deep manual inspection of the MS and MS/MS spectra, cleaning the background noise, establishing the potential fragmentation patterns based on the neutral losses observed, and selecting the characteristic fragment ions for de novo structure elucidation, attempting to reach a final annotation or unveil the compound type that we were facing. As a result, we could accurately annotate seven 2-hydroxy fatty acids and seven 3-hydroxy fatty acids thanks to the work published by Jiangshuo Li and colleagues^30^. Some of the unknown features we obtained were adducts related to the mobile phase composition employed for this study, which is still seen as a pitfall in LC-MS^3^.

### The annotation workflow demonstrated its efficacy in precisely annotating hundreds of lipid species

**Fig. 4:**
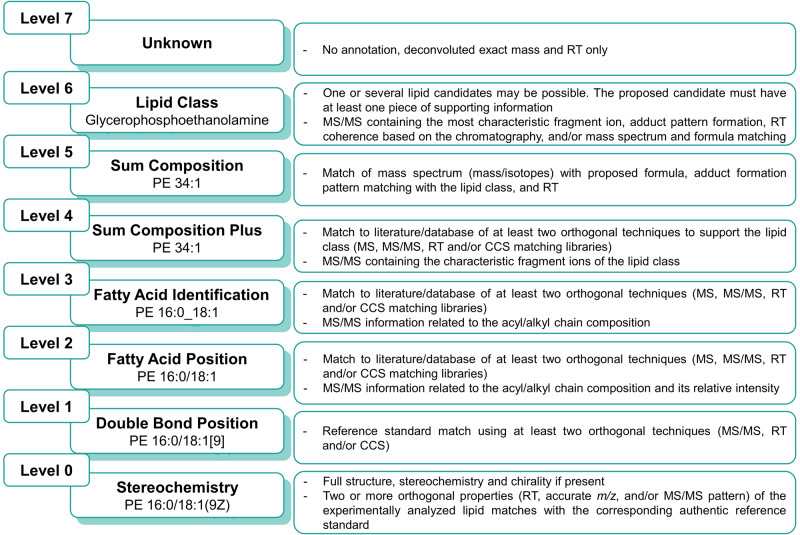
The confidence levels of lipid annotations used in this work were based on the degree of structural detail accurately assigned to the molecule. An example of the different annotations a PE lipid could obtain based on the structural details achieved is shown in the figure.

By optimizing the LC-MS settings and implementing our annotation workflow, we significantly improved the lipidome analysis in mice lung tissue. Initially, the four software tools suggested 1409 lipid annotations. After merging the results and eliminating redundant data but keeping the information of the number of programs that rendered such annotation, we obtained 1022 lipid annotations. After applying the annotation workflow, we finally obtained 866 accurately annotated lipids (Supplementary Table 1). As previously described, the lipid annotation/identification hierarchy is determined based on the level of structural information that can be accurately assigned to the molecule^31^. In this study, we established a seven-level classification system for lipid annotation (Fig. 4). Level ‘0’ indicates that the complete structure and stereochemistry of the lipids were confirmed with a reference standard and special experimental approaches, such as chemical derivatization before the analysis, hydrolyzation of FAs to their methyl esters, the use of high-energy collision-induced dissociation, silver ion chromatography, multistage mass spectrometry, or covalent adduct chemical ionization^32^, while Level ‘1’ signifies that the double bond position was specified. Level ‘2’ was assigned when the fatty acyl/alkyl composition could be described, Level ‘3’ when the exact position of the fatty acyl/alkyl chains could be identified, Level ‘4’ when only the sum composition was provided by the MS/MS data, and Level ‘5’ when the sum composition was obtained with the MS data, Level ‘6’ when only the lipid class was determined, and finally Level ‘7’ for unknown features. Of the 866 annotated lipid molecular species, only one reached confidence level ‘1’, 254 were assigned to confidence Level ‘2’, 514 to Level ‘3’, and 97 to Level ‘4’ (Supplementary Data 1).

**Fig. 5:**
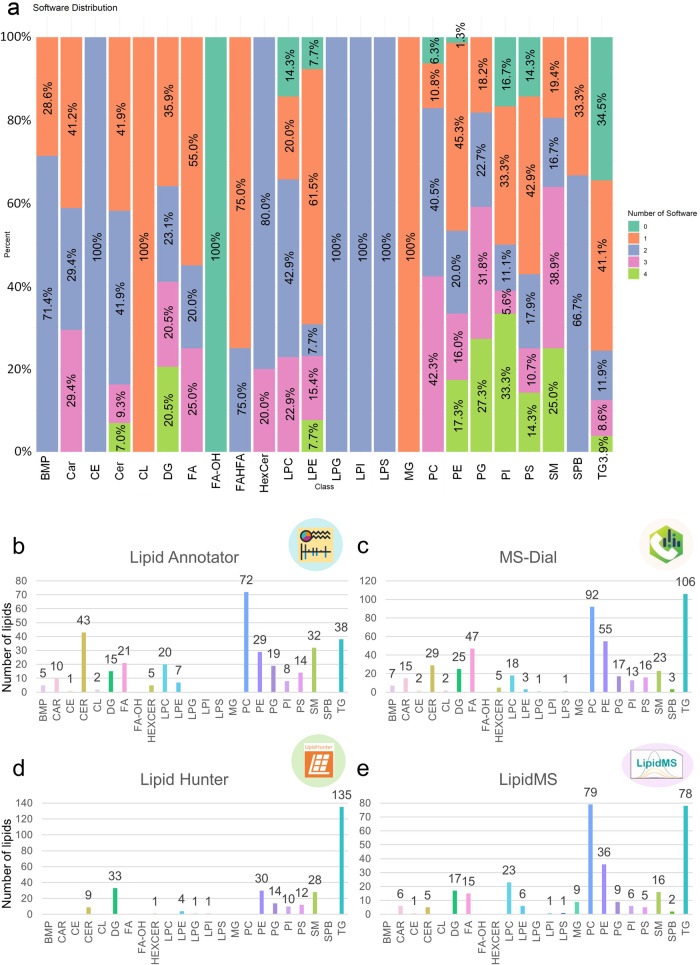
Schematic representation of the software tools performance. **a** Graphical representation of the software distribution in terms of the annotated lipid species. The bars display the percentage of lipid species within each lipid class annotated by four (light green), three (pink), two (blue), or one (orange) software. Finally, the dark green color corresponds to lipids annotated by manual chromatogram and MS/MS spectra inspection. The figures from (**b**–**e**) represent the lipid species annotated by each software tool (Lipid Annotator, (**b**); MS-Dial, (**c**); LipidHunter, (**d**); LipidMS, (**e**)).

Surprisingly, none of the four software tools could annotate lipid species from all the lipid classes included in our database. Most of the lipids were found by just one or two software tools (see Fig. 5a). For example, cholesterol esters (CE), monoacylglycerophosphoglycerols (LPG), monoacylglycerophosphoinositols (LPI) and monoacylglycerophosphoserines (LPS) were identified by two software. In the case of glycerophosphoglycerophosphoglycerols (CL) and monoradylglycerols (MG), they were found by just one software. However, some of them, such as the hydroxy-fatty acids (FA-OH), and some glycerophospholipids (GP), were found thanks to the manual inspection of the chromatograms and MS/MS spectra using the CEU Mass Mediator^33^ online tool and the MassHunter Qualitative software v10.0 (Agilent Technologies). Then, the results obtained from each tool were plotted independently to verify each software’s annotation capacity, letting us determine the strengths and weaknesses of each one of them (Fig. 5b–e). For instance, MS-Dial and Lipid Annotator offered a higher number of annotated lipids (480 and 341, respectively) compared to LipidMS and LipidHunter (315 and 278, respectively). In contrast, the number of false positive annotated compounds was also higher in MS-Dial than in the other three software detected thanks to the manual inspection of the MS and MS/MS spectra (Supplementary Table 2). Despite this limitation, MS-Dial has been used to develop Lipidome ATLAS in various tissue matrices^10^.

MS-Dial is the software that provides the highest number of annotated lipids, representing 55% of the 866 lipids collected in our library (Supplementary Table 2, Supplementary Data 2). However, this is too far from the complete lipidomic profile of the mice lung sample, making the information obtained from the other software and the manual inspection necessary to complete the picture. After analyzing the lipid families detected by each software, we found that Lipid Annotator was more effective in annotating sphingolipids (SP) (ceramides (Cer) and spingomyelins (SM)) and monoacylglycerophospholipids (LGP) (monoacylglycerophosphocholine, (LPC) and monoacylglycerophosphoethanolamine (LPE)). On the other hand, MS-Dial provided more comprehensive information about GP (glycerophosphocholines (PC), glycerophosphoethanolamines (PE), glycerophosphoinositols (PI), glycerophosphoserines (PS), LPG, and LPS) and fatty acids (FA). Both MS-Dial and LipidHunter detected most triradylglycerols (TG) and diradylglycerols (DG) in our samples, with LipidHunter providing complementary information about SM lipid species. Notably, LipidMS was the only software that identified MG and some LGP.

We observed that not all the software tools could annotate the same lipid species in the same proportion, sometimes giving different information. Despite the differences observed, by combining multiple annotation software and gathering all the information they provided, we could obtain a broader view of the lipid landscape of our biological matrix, allowing us to optimize the time and obtain more information. Furthermore, we emphasize that using only one annotation software tool without thoroughly inspecting the results might lead to inaccurate annotations and, consequently, a bias in the biological interpretation.

### The semi-quantification of the mice lung lipidome allowed us to generate LiLA: a Lipid Lung-based ATLAS

The lipidomics community has recently emphasized the importance of precise quantification to harmonize lipidomics data and promote data comparability. By combining lipid quantification with comprehensive resources detailing lipid-species profiles for various biological matrices, including murine tissues, standardization can significantly contribute to advancing our comprehension of the functional significance associated with specific lipid species^34^.

Achieving optimal quantitative accuracy necessitates the utilization of isotopically labeled ISs for every molecular species present in the sample. Regrettably, implementing this approach across the entire lipidome remains unfeasible at present. In this study, we conducted semi-quantification of the mice lung lipidome covering 866 previously annotated lipid species by employing lipid-subclass-specific internal standards (ISs). These ISs were calibrated at six different concentrations each closely resembling those of the endogenous analytes to design the calibration curves that were spiked with a pool of lung tissue samples (Supplementary Table 3 and Supplementary Fig. 2). The standards for the calibration curve and the pool of lung tissue samples were analyzed in triplicate following the same analytical workflow described above. Briefly, the pool of lung tissue samples was divided into six different Eppendorf tubes and treated for lipid extraction following the protocol described in the *Materials and Methods* section. Once we added the ISs at the six different concentrations, the vials were then introduced in the RP-UHPLC-ESI-QTOF MS for the analysis in both ion modes. The raw data obtained was then processed using the already elaborated list of 866 lipids using the Batch Targeted Feature Extraction mode of the Agilent MassHunter Profinder software to integrate the area of each lipid, including the ISs. The area obtained from each IS was plotted to obtain the calibration curves (Supplementary Fig. 2). Finally, the lipids present in the healthy mice lung samples were semi-quantified by interpolating the area in the corresponding IS calibration curve equation to obtain concentration. The lipids that did not have a class specific IS included in the mixture were semi-quantified by utilizing a standard compound with the slightest deviations in RT and mass compared to the corresponding biological compound^35^. The IS compound used to semi-quantify each lipid subclass is described in Supplementary Data 3. At the end of the process, 709 lipid molecular species were semi-quantified, providing an extensive and detailed characterization of the lung lipidome (Supplementary Data 3). A semi-quantification of hundreds of lipid species in lung tissue allows for comparative studies between experimental conditions, disease models, and patient cohorts. Moreover, this data can be integrated with other omics data (genomics, transcriptomics, proteomics) for better understanding of lung biology and identify potential connections between lipid metabolism and other cellular processes.

### LiLA potentiated to shed light on the mice lung lipidome landscape

**Fig. 6:**
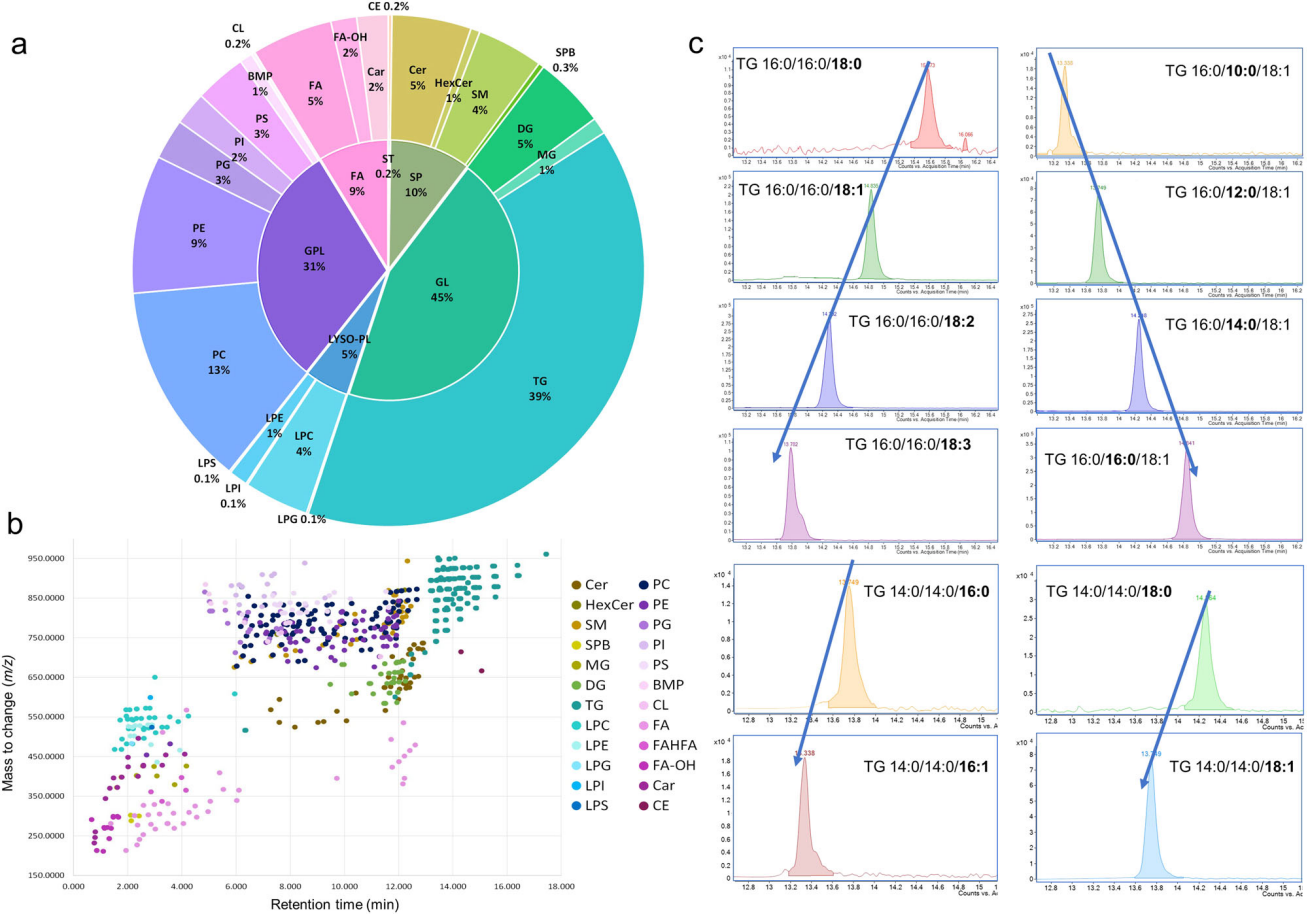
Healthy mice lung tissue lipidome landscape. **a** The circle diagram illustrates the relative proportions of lipid classes detected in our samples. The lipid class distribution was determined using a four-annotation software combined with manual inspection of the spectra. The inner circle of the diagram represents the lipid categories, while the outer circle denotes the specific lipid classes. The size of each section within the diagram corresponds to the abundance percentage of the respective lipid class. **b** RT mapping plot depicting each lipid species precursor’s *m/z* and RT across the 19 min chromatographic run. Lipid classes are denoted by color. **c** Example of the manual inspection process performed for several TG lipid species to check the elution order based on the carbon atom number and the degree of unsaturation.

Owing to the development of our LiLA workflow, we could accurately annotate lipids from twenty-three different lipid classes (see Fig. 6), including GP, glycerolipids (GL), SP, Fatty Acyls, among many other species. The lipid class proportion depends on several factors, including the analytical conditions, the complexity of the biological matrix and the resolving power to distinguish between the different isomeric lipid species and the co-elution of lipids. It is important to note that lipids are the end products of various biochemical pathways within the cells and are heavily influenced by the biological environment^36^. As a result, the abundance of different lipid species can fluctuate depending on the metabolic state of the organism under investigation and the specific sample composition. In our study, we focused on the lungs, a heterogeneous organ composed of up to 40 different cell types, each one with a specific lipid profile distribution^37^.

The lungs are a crucial part of the respiratory system, absorbing oxygen, and expelling carbon dioxide. In this regard, pulmonary surfactant is critical in maintaining normal lung function by reducing surface tension at the air-liquid interface in alveolar spaces and enhancing an efficient gas exchange. Equally important, the lungs are exposed to inhaled particles and microorganisms from birth. Therefore, cilia, mucus, and the cough reflex are the first-line lung defenses before immunity to prevent pathogen access to the lower airways and avoid an overt inflammatory response. This is one of the principal reasons why lungs are so sensitive to lipid metabolic equilibrium since pulmonary physiology relies on lipids for important extracellular activities ensured by surfactant and consisting of sphingolipid/glycerolipid network^38^. In our work, GL and GP constituted 45% and 31% of the total lipid content detected in healthy mice lung samples (Fig. 6a). In general terms, the pulmonary surfactant is composed of a mixture of 80% GP, 5–10% neutral lipids, and 8–10% proteins. Regarding the GP, PC represented 13% of the total GP lung content. It could be related to the pulmonary surfactant composition, in which PC represents 80-85% of its mass. More specifically, palmitoyl-containing PCs were detected as the most abundant lipid species in the lung tissue, including PC 16:0/16:0 (3156.72 ng/mg), PC 16:0/18:1 (1630.01 ng/mg), PC 16:0/18:2 (1344.03 ng/mg), PC 16:0/16:1 (1194.09 ng/mg), and PC 16:0/14:0 (555.36 ng/mg). PC 16:0/16:0 is the main lipid component of surfactant, which saturated acyl chains allow it to pack tightly at the air/liquid interface, producing maximum surface tension reduction at the end exhalation and lead to stabilization of open lungs^39,40^. In addition to PC, other phospholipids found in pulmonary surfactants, such as PE, play a crucial role in facilitating and promoting curvature in non-bilayer surfactant forms, which are essential intermediates during the transitions from bilayers to interfacial films and their interconversions throughout surfactant metabolism. Meanwhile, PI has been shown to enhance the rate of alveolar fluid clearance and stabilize the surfactant monolayer, making it an essential component of pulmonary surfactant. Additionally, the PG are responsible for the modulation of the macrophage’s function and lung maturity^41^.

In the lung tissue, TG accounted for 39% of the total GL content (Fig. 6a). As previously reported, TG have been implicated in lung surfactant metabolism as a potential source of fatty acids for phospholipid synthesis^42^. Additionally, since the lungs require substantial amounts of energy to perform their functions, it is reasonable to hypothesize that energy-storage lipids would be present in considerable proportions^43^. Paying attention to the SP, we can highlight their critical role in maintaining the structural integrity of the lungs, cell survival and stress response^38^. In addition, they are closely related to inflammatory reactions, and multiple studies have shown that sphingolipid levels are altered in various lung diseases, such as asthma and chronic obstructive pulmonary disease (COPD). It has been shown that increasing levels of Cer favor pathological hyperinflammation in the lungs^38^. Cer can promote the activation of immune cells, such as macrophages, and the production of pro-inflammatory cytokines, such as TNF-alpha and IL-6^44^. This can help to amplify the immune response to the infection, but it can also contribute to lung damage and inflammation. In addition to their roles in the immune response, SP have also been implicated in the pathogenesis of pulmonary infections. For instance, specific pathogens, such as *Neisseria gonorrhoeae* and *Pseudomonas aeruginosa*, have been shown to exploit the hydrolysis of cell surface SM to generate ceramide-rich microdomains that serve as portals for their entry into macrophages, promoting their growth and survival in the lungs^45,46^. These SP metabolites can also interfere with the host immune response, allowing the pathogen to evade detection and clearance^38^. Based on previous facts, it is not surprising that the proportion of SP was also high in the lung samples due to its crucial implications for the lung function.

To obtain a comprehensive understanding of the lipid composition in mice lung tissue, we utilized a variety of visualization approaches. The first approach involved plotting the different lipid classes based on their *m/z* and RT in minutes, as demonstrated in Fig. 6b. This visualization technique enabled us to rapidly categorize the lipid classes that varied in terms of the number of carbon atoms and/or degree of unsaturation. By examining the elution patterns of the lipid classes based on the specific chromatography employed in this study, we could identify any potential false-positive annotation suggested by the software by assessing their alignment with the expected RT values. Furthermore, we manually inspected the data to enhance the confidence level of our annotations, as illustrated in Fig. 6c. This involved examining the RT values of different lipid species within a specific lipid class, considering factors such as the number of carbon atoms in their acyl chains and the degree of unsaturation. To better classify the lung lipidome profile, we plotted the intensity and degree of unsaturation for each lipid, classified by lipid class (refer to Supplementary Fig. 3). This meticulous analysis allowed us to refine our annotations and increase their reliability.

### The hybrid lipidomics strategy unveiled the lipid dysregulation induced by Mtb and the TB progression

Before the onset of the COVID-19 pandemic, Tuberculosis (TB), caused by the pathogen *Mtb*, was ranked as the top cause of death from infectious disease worldwide, surpassing even HIV/AIDS. In 2020 alone, an estimated 1.5 million individuals died of TB, imposing an important health and socio-economic burden on low- and middle-income countries. Gaining a deeper understanding of the pathogenesis of TB hinges on investigating its epicenter: the granuloma. This biological matrix represents an ideal medium for biomarker discovery as it directly reflects the local changes induced by *Mtb* infection or TB drug therapy in the host. However, our knowledge of metabolic alterations in lung tissue samples remains limited. Unfortunately, the need for more studies based on lung tissue samples can be attributed to the highly invasive nature of sample collection and the limited amount of tissue that can be obtained. In this regard, an in-depth examination of the lipid alterations induced by the *Mtb* in the host lipidome during the infection might unveil potential new biomarkers for better disease characterization and provide new insights toward developing improved diagnostic and therapeutic approaches.

The *Mtb*-infected mice lung samples collected at two-time points, four- and twelve-weeks post-infection (*Mtb*^*+*^*4w*, *Mtb*^*+*^*12w*), were analyzed using the RP-UHPLC-ESI-QTOF MS-based approach to unveil the lipid dysregulation induced by TB. The already developed Lipid Lung-based Atlas (LiLA) was used to reprocess the untargeted data using the Batch Targeted Feature Extraction mode of the Agilent MassHunter Profinder software, from which 741 features for ESI (+) and 445 features for ESI (-) were obtained, being some of them commonly shared between the two ionization modes. The obtained data matrices were filtered based on the coefficient of variation (CV) in the QCs, keeping features with CVs lower than 30%. Data sets after filtering consisted of 738 features for ESI (+) and 440 for ESI(−). Subsequently, each feature’s percentage of change was calculated to observe the main alterations induced by the *Mtb* infection and the trend of such dysregulations due to the evolution of infection.

After performing multivariate (MVDA) data analysis, the initial PCA plots generated for both ESI(+) and ESI(−) modes showed the QCs as tightly clustered, ensuring the stability and reproducibility of the system (Supplementary Fig. 4a, b). After that, the supervised PLS-DA plots showed clear discrimination among the three groups, where the *Mtb*^*+*^*4w* post-infection group was located in between the *Mtb*^*+*^*12w* and the *Mtb*^−^ group (Supplementary Fig. 4c, d). Finally, we generated supervised OPLS-DA (Supplementary Fig. 4e, f for *Mtb*^*+*^*4w vs Mtb*^*-*^ and Supplementary Fig. 4g, h for *Mtb*^*+*^*12w vs Mtb*^*-*^) to shed light on the most affected lipids that can be used to determine the principal lipid networks altered depending on the progress of infection. All the PLS-DA and OPLS-DA models displayed suitable quality parameters (explained variance, *R*^2^ ≥ 0.6; and predicted variance, *Q*^2^ ≥ 0.4) with a difference among them lower than 0.3^47^. The quality parameters and the CV-ANOVA p-value found in the legend confirmed the reliability of these models. From the OPLS-DA models, we also extracted the VIP value, which refers to the contribution and relevance of each lipid in the model. Those lipids with a VIP value lower than one were not considered relevant for the model. In addition, we performed univariate (UVDA) statistical analysis using MATLAB (R2022a, MathWorks) to evaluate the significance of each lipid concerning the biological variability, obtaining a p-value of each lipid species within each studied biological condition (Mann Whitney U test), and a general p-value comparing the three groups (Kruskal Wallis test). After data normalization, filtration by CV in QC samples (<30%) and based on the VIP threshold (VIP > 1.0) and *p* ≤ 0.05 in Mann–Whitney *U* test, 66 and 68 lipids were found to be statistically significant when comparing *Mtb*^*+*^*4w* with the *Mtb*^*-*^ group in ESI(+) and ESI(−), respectively. Then, 119 and 79 lipids were found to be statistically significant when comparing *Mtb*^*+*^*12w* with the *Mtb*^*-*^ group in LC-MS ESI(+) and LC-MS ESI(−), respectively (Supplementary Data 4).

**Fig. 7:**
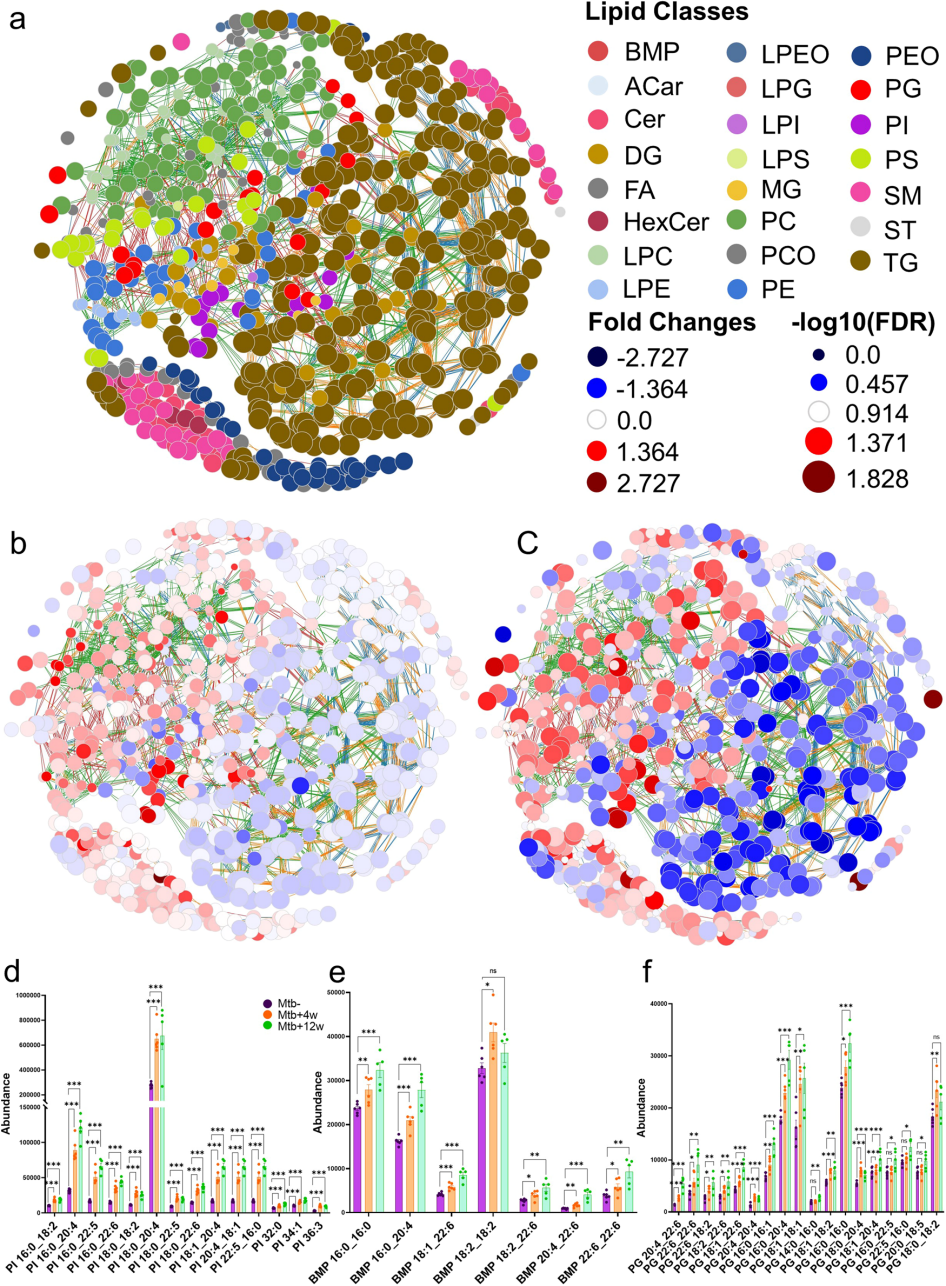
Lipid dysregulation induced by *Mtb* infection and the TB progression. **a** LINEX Lipid network of the mice lung tissue with node colored by lipid classes. Keeping the same lipid distribution within the networks, plots B and C represent node size scaled by −log10 of FDR and colored by fold change for the comparison between (**b**) *Mtb*^*+*^*4w* (n = 6) vs *Mtb*^−^ (*n* = 6), and (**c**) *Mtb*^*+*^*12w* (*n* = 5) vs *Mtb*^−^ (*n* = 6). Blue colors indicate lower concentrations of lipids. All edge connections are colored by reaction type as follows: chain length (blue), desaturation (orange), FA addition (green), head group modification (red). **d**–**f** Bar chart with the experimental values of PI (**d**), BMP (**e**), and PG (**f**) lipid species in each group. The error bars represent the standard error of the mean (SEM). *p ≤ 0.05; **p ≤ 0.01; ***p ≤ 0.001; ns, not significant in the comparison.

Once the final data matrix was obtained, the Lipid Network Explorer (LINEX) web tool (https://exbio.wzw.tum.de/linex/) was used to visualize and analyze functional associations of lipids on networks, enabling the study of the lung lipidome in the context of metabolic reactions^48^ (Fig. 7a–c). Figure 7a shows a general overview of the functional associations of lipids on networks in healthy mice lung samples, where a different colour was assigned to each lipid subclass. Figure 7B, C corresponds to the same functional lipid association but, in this case, we also compare the changes observed in the levels of each lipid species, *Mtb*^*+*^*4w vs Mtb*^−^*and Mtb*^*+*^*12w vs Mtb*^−^, respectively. At first glance, it can be observed that there is a general decrease on the levels of TG and DG when comparing the *Mtb*^*+*^*4w* group with the *Mtb*^−^ (Fig. 7b, c). The decrease on their levels was more profound after 12 weeks post-infection (*Mtb*^*+*^*12w vs Mtb*^−^). The decrease of TG and DG levels in response to *Mtb* infection indicates that the pathogen may utilize these lipid species as a carbon source during its intracellular survival within host cells^49^. On the other hand, most of the GPs were found to increase, showing a more considerable increment in their levels after 12 weeks post-infection. Notably, we observed a considerable increase in the levels of two specific PI species, namely PI 18:1_20:4 and PI 16:0_20:4, in the *Mtb*^*+*^*4w* group compared to the healthy control group (Fig. 7d). The levels of these PI species showed a remarkable increment of 214% and 170%, respectively. Furthermore, in the *Mtb*^*+*^*12w* post-infection group, the levels of PI 18:1_20:4 and PI 16:0_20:4 exhibited a more pronounced increase, reaching increments of 309% and 243%, respectively, compared to the healthy control group. These findings are of particular interest considering the unique lipid composition of the *Mtb* plasma membrane. In *Mtb*, PI and its metabolically derived products, such as mannosylated phosphatidylinositol lipids, account for more than 50% of the total lipid content^50^. In fact, mannosylated phosphatidylinositol specifically increases the homotypic fusion of endosomes and endosome-phagosome fusion, suggesting meaningful interferences with the endo-lysosomal network during *Mtb* infection. Bis(monoacylglycero)phosphates (BMP) are widely recognized as lysosomal markers that play a central role in determining the fate of lysosomal content by promoting the degradation and sorting of lipids. Consistent with previous reports, the lysosomal content and activity are globally elevated in *Mtb*-infected macrophages over time and defines an adaptive homeostasis in the infected macrophage^51^. Our results demonstrated a great increment in the level of BMP lipids (Fig. 7e) and their precursors, the PGs (Fig. 7f), especially after 12 weeks post-infection. Therefore, the relevant elevation observed in the levels of these BMP, PI, and PG lipid species highlight the global alteration of the host lysosomal system, which is a defining feature of *Mtb*-infected macrophages, suggesting their potential involvement in the host-pathogen interaction and the pathogenesis of TB infection. Although their distribution among the various biological organelles is different, SP are mainly enriched at the outer leaflet of the plasma membrane. Therefore, pathogens, including *Mtb*, inevitably interact with this class of lipids during phagocytosis. As shown in Fig. 7b, c, Cer and HexCer levels were also increased in response to *Mtb* infection. Ceramides, critical intermediates in the biosynthesis of complex SP, are highly involved in cellular signaling processes, especially in regulating apoptosis, and cell differentiation, transformation, and proliferation. The most remarkable increments detected when comparing both post-infection time points with the control group were displayed by the Cer 18:0;O2/16:0 (113% *Mtb*^*+*^*4w vs Mtb*^*-*^*;* 196% *Mtb*^*+*^*12w vs Mtb*^−^), Cer 18:0;O2/24:1 (102% *Mtb*^*+*^*4w vs Mtb*^*-*^*;* 152% *Mtb*^*+*^*12w vs Mtb*^*ESI(−)*^) and HexCer 18:1;O2/16:0 (325% *Mtb*^*+*^*4w vs Mtb*^*-*^*;* 290% *Mtb*^*+*^*12w vs Mtb*^*ESI(−)*^). These results align with the reported increment in the Ceramide levels in mycobacteria-induced necrotic lung granulomas^52^. All these findings add to our understanding of the metabolic adaptations *Mtb* employs to sustain its growth and persistence within the host environment. Further investigations into the mechanisms underlying these alterations, as well as the precise role of these lipid species in *Mtb* infection, would provide valuable insights into the interplay between host lipid metabolism and *Mtb* pathogenesis. Moreover, exploring potential therapeutic strategies to modulate these lipid pathways could offer new avenues for combating TB and limiting the intracellular survival of *Mtb*.

A well-established and specific annotation workflow plays a vital role in ensuring the accuracy and reliability of lipid annotation. Our workflow, integrating four software tools for lipid annotation and a decision-tree-based approach, has proven its efficacy in enhancing the reliability of lipid annotations obtained in untargeted lipidomics studies. Moreover, the synergistic combination of the Hybrid-lipidomics strategy and the semi-targeted approach has enabled a comprehensive and multi-dimensional analysis of the lung lipidome. Notably, LiLA has provided valuable insights into specific lipid signatures induced by *Mtb* infection in the lung. This exemplifies the significance of LiLA in the diagnosis, prognosis, and selection of therapeutic targets for lung-related disorders, including pulmonary diseases, infections, and lung cancer. The robustness and utility of our approach pave the way for advancing lipidomic research and its application in understanding complex biological systems and identifying potential biomarkers for lung-related diseases.

## Methods

### Animals

Animals were divided into three different groups: healthy controls (*Mtb*^−^, *n* = 6), infected mice four weeks post-infection (*Mtb*^*+*^*4w*, *n* = 6) and infected mice twelve weeks post-infection (*Mtb*^*+*^*12w*, *n* = 5). Seven- to eight-week-old female BALB/c mice were purchased from Jackson Laboratories. Mice were housed in a pathogen-free facility with ad libitum access to water and food. All procedures were conducted according to NIH guidelines and protocols were approved by the Institutional Animal Care and Use Committee of the University of Alabama at Birmingham.

Mice were aerosol infected with *Mtb H37Rv* using the aerosol inhalation exposure system (Glas-Col, USA) to deliver ~120–250 CFU/mouse lung. The infection dose was estimated by enumerating the lung CFU at 24 h post-infection. Mice were sacrificed using anesthesia with isoflurane followed by gentle cervical dislocation. Mice organs were aseptically harvested and homogenized in 2 ml of 1× PBS, pH 7.4. Serial dilutions of homogenates were prepared in 1× PBS and plated on 7H11 agar plates supplemented with 10% ADS (Albumin, Dextrose, and NaCl), Carbenicillin (25 mg/L) and Cycloheximide (25 mg/L). Plates were incubated at 37 °C for ~21 days before counting colonies. Finally, lung samples were transferred to an N_2_(l)-containing recipient to freeze the tissues and stored at −80 °C to avoid postmortem metabolic processes.

### Reagents

LC-MS grade methanol (MeOH), acetonitrile (ACN), and isopropanol (IPA) were obtained from Fisher Scientific (Pennsylvania, United States). Methyl-tert‑butyl ether (MTBE) and ammonium fluoride (NH_4_F) (ACS reagent, ≥ 98%) were purchased from Sigma‐Aldrich (Steinheim, Germany). Ammonia solution (28%, GPR RECTAPUR®) and acetic acid glacial (AnalaR®NORMAPUR®) were obtained from VWR Chemicals (Pennsylvania, United States). Reverse‐osmosed ultrapure water, used to prepare all the aqueous solutions, was obtained from a Milli‐Qplus185 system (Millipore, Billerica, MA, USA). 9,12,13-TriHOME commercial standard (Cayman Chemical, MI, USA).

### Sample Treatment for Lipid Extraction

The sample preparation and lipid extraction were performed at the Centers for AIDS Research and Free Radical Biology, University of Alabama at Birmingham (Birmingham, AL, United States), following a protocol initially described and optimized at CEMBIO (Madrid, Spain)^53^. Briefly, approximately 75 mg of lung tissue was mixed with a cold (–20 °C) mixture of MeOH:H_2_O (1:1, v/v) added in a ratio of 1 mg tissue:10 µL of extraction solvent. Next, the tissue samples were homogenized using Dounce homogenizer. After the homogenization, 200 µL of homogenate was mixed with 640 µL of MeOH and 160 µL of Methyl-Tert-Butyl ether (MTBE) to extract hydrophobic compounds^49,53^. Samples were then vortex-mixed for 1 h at room temperature (RT) and centrifuged at 4000 g for 20 min at 20 °C. The samples were then passed through spin X columns (0.22 µm filter), and 200 µL of the filtered sample was dried at RT in the vacuum concentrator. From here, the samples were sent to CEMBIO for the UHPLC-MS analysis. Before the analysis, dried samples were re-suspended with 200 µL of MeOH/MTBE/H_2_O (7.4:1.6:1, v/v/v), which contained the corresponding ISs (C_17_-sphingosine at 1 ppm for positive ion mode, and d_31_-palmitic acid at 3 ppm for negative ion mode). Samples were then centrifuged (16,100*g*, 5 min, 15°C) before transferring them into sample vials with glass inserts for LC-MS analysis.

### Quality Control Samples

Quality Control (QC) samples were prepared by pooling equal volumes, 20 µL in our case, of each prepared lung sample and were processed identically in parallel with the rest of the study samples. Then, 100 µL of the pooling mix was placed into a UHPLC-MS chromatography vial with an insert, which was analyzed throughout the run to provide information about the system’s stability and performance and the reproducibility of the sample treatment procedure^54^. Four blank samples were prepared along with the rest of the samples, following the same lipid extraction procedure. The blank samples were then analyzed at the beginning and at the end of the analytical sequence to identify common contaminations. Finally, the analysis of lung extracts was performed on an Agilent 1290 Infinity II UHPLC system coupled to an Agilent 6545 quadrupole time-of-flight (QTOF) mass spectrometer in both positive and negative ion modes using the analytical conditions previously described^3^ and also detailed below.

### Semi-quantification

Semi-quantification of lipids was performed using Agilent MassHunter Profinder software (B.10.0.2, Agilent Technologies, Santa Clara, CA, USA) and Microsoft Excel 2016. First, 6-point calibration curves were designed for each standard, covering the concentration range of the native lipids of the corresponding lipid class while still displaying a linear behavior of ISs in the concentration-response relationship. For this purpose, pooled healthy lung tissue samples were spiked with SPLASH® Lipidomix® Mass Spec Standard mixture (Avanti) amounts to reach the needed concentration. Next, the peak areas from the lipid species contained in the IS mixture were extracted using the Batch Targeted Feature Extraction mode. Afterwards, the calibration curves were generated for each standard and only calibration points resulted in a calibration curve with R > 0.990 were approved.

### Lipidomics analysis

#### Analytical conditions selected for the RP-UHPLC-ESI-QTOF MS lipidomics analysis

The analytical platform selected for data acquisition was an Agilent 1290 Infinity II Ultra-High Performance Liquid-Chromatography (UHPLC) system coupled to an Agilent 6545 quadrupole time-of-flight (QTOF) mass spectrometer. We used an Agilent InfinityLab Poroshell 120 EC –C18 (3.0 × 100 mm, 2.7 μm) (Agilent Technologies) column and a compatible guard column (Agilent InfinityLab Poroshell 120 EC –C18, 3.0 × 5 mm, 2.7 μm), both held at 50 °C. The Agilent 1290 Infinity II Multisampler system was used to uptake 1 μL of extracted samples, maintaining the temperature at 15 °C to preserve compounds and avoid lipid precipitation. The mobile phases used for both positive and negative ionization modes consisted of (A) 10 mM ammonium acetate, 0.2 mM ammonium fluoride in 9:1 water/MeOH and (B) 10 mM ammonium acetate, 0.2 mM ammonium fluoride in 2:3:5 acetonitrile/MeOH/isopropanol. The multi-wash strategy consisted of a mixture of methanol:isopropanol (50:50, v/v) with the wash time set at 15 s, and an aqueous phase:organic phase (30:70, v/v) mixture to assist in the starting conditions. The chromatography gradient started at 70% of B at 0–1 min, 86% at 3.5 –10 min, and 100% B at 11–17 min. The starting conditions were recovered at minute 17, followed by a 2 min re-equilibration time; for a total running time of 19 min. The flow rate during the analysis was kept constant at 0.6 mL/min.

The parameters of the Agilent 6545 QTOF mass spectrometer equipped with a dual AJS ESI ion source were: 150 V fragmentor, 65 V skimmer, 3500 V capillary voltage, 750 V octopole radio frequency voltage, 10 L/min nebulizer gas flow, 200 °C gas temperature, 50 psi nebulizer gas pressure, 12 L/min sheath gas flow, and 300 °C sheath gas temperature. Data were collected in positive and negative ESI modes in separate runs, operated in full scan mode from 40 to 1700 *m/z* with a scan rate of 3 spectra/s. A solution consisting of two reference mass compounds was infused throughout the whole analysis: purine (C_5_H_4_N_4_) at *m/z* 121.0509 for the positive and *m/z* 119.0363 for the negative ionization modes; and HP-0921 (C_18_H_18_O_6_N_3_P_3_F_24_) at *m/z* 922.0098 for the positive and *m/z* 980.0163 (HP-0921 + acetate) for the negative ionization modes. These masses were continuously infused into the system through an Agilent 1260 Iso Pump at a 1 mL/min (split ratio 1:100) to provide a constant mass correction.

Additionally, the iterative-MS/MS acquisition mode was performed for both positive and negative ion modes. We performed ten runs of a selected sample using two different collision energy, 20 eV and 40 eV (five measurements per voltage). For each run, the software selects the three more intense precursor ions, which were fragmented to obtain the spectrum for a specific time point. In the subsequent measurement of the same sample, for the same specific time point, the software excluded the previous three selected ions and selected the following three more intense precursor ions. By measuring the sample several times, we obtained thousands of MS/MS spectrums at the end, covering most of the broader lung lipidome spectrum^3^.

### Software programs parameters

- *Lipid Annotator v1.0* (Agilent): The method parameters used in the data processing with Lipid Annotator were as follows: Q-Score ≥ 20.0, adduct selection H^+^, Na^+^ and NH_4_^+^ for positive ion mode and H^-^ and C_2_H_3_O_2_^−^ for negative ion mode. As we were performing an untargeted analysis, we selected all lipid classes to perform the search against the database. For the ID parameters, the Mass Threshold was set at mass deviation ≤20.0 ppm, the “Report top candidate only” option was selected, the Fragment score was ≥30, the Total score was ≥60, and the Constituent Level was ≥ 10%.
- *MS-DIAL 4* (Riken): the raw vendor-format data files or the common mzML data need to be converted into “.ibf” files using the MS IBF file Converter software. For MS/MS data reprocessing, the following parameters were selected: soft ionization for LC-MS/MS, chromatography separation type, conventional LC-MS method type, and profile data as the data type. We selected the corresponding ionization mode (positive or negative) for each analysis and Lipidomics as the target omics. The MS1 and MS/MS *m/z* detection window was set at 40–1700 Da, and the retention time window was set at 0–19 min. The peak detection window, smoothing level was set as 1 scan. The Accurate mass tolerance was 0.01 Da for MS1 and 0.025 for MS2 for the Identification window, and the identification score cut off was set at 70%. Next, we selected specific adducts depending on the ionization mode that we were analyzing (H^+^, Na^+^ and NH_4_^+^ for positive and H^-^ and C_2_H_3_O_2_^-^ for negative ion mode). The rest of parameters were set as default.
- *LipidHunter*: The parameters used for the analysis were 0–19 min as the scan range, 40–1700 *m/z* range, ±0.75 *m/z* precursor window, DDA Top 6, ±20 ppm for MS tolerance level, the absolute intensity for the MS level threshold was set at 1000, ±20 ppm MS/MS tolerance level, the absolute intensity for the MS/MS level threshold was set at 10, 80% isotope score, 75% Rank score and 0.10% as the minimum relative intensity for the scoring.
- *LipidMS 3.0:* We reprocessed our data by selecting the Batch processing option in the corresponding ionization mode. The *m/z* tolerance for MS1 and MS/MS was set at 20 ppm. The tolerance for the RT window was set at 30 s. The rest of the parameters were set as default. Finally, the lipid classes selected for the annotation process were established according to the ionization mode.

### Hybrid lipidomics analysis

To illustrate the benefit of counting with an extensive lipid database of a particular biological matrix, we investigated the lipid alterations induced by the *Mtb* infection at two different time points (4 weeks, *Mtb*^*+*^*4w* and 12 weeks, *Mtb*^*+*^*12w*) post-infection. The previously obtained database, including molecular formulas, mass, and RT information, was imported into the Agilent MassHunter Profinder software (B.10.0.2, Agilent Technologies, Santa Clara, CA, USA) using the Batch Targeted Feature Extraction mode to perform the feature extraction and time alignment. Features were built as the sum of coeluting ions that are related by charge-state envelope, isotopologue pattern, and/or the presence of different adducts and dimers in the analyzed samples. To detect coeluting adducts of the same feature, the following adducts were selected: [M + H]^+^, [M+Na]^+^, [M + K]^+^, [M + NH_4_]^+^ and [M + C_2_H_6_N_2_ + H]^+^ in LC-ESI(+)-MS; [M-H]^−^, [M+Cl]^−^, [M + CH_3_COOH-H]^−^, and [M + CH_3_COONa-H]^−^ in LC-ESI(-)-MS.

### Statistical analysis

Before proceeding with statistical analysis, we implemented data normalization and filtration processes to ensure data quality. Features with mean blank values above 10% of the mean value in the samples were removed. The raw data matrices were then normalized by the IS to account for unwanted variance due to sample preparation and the analytical run. Then, the features were selected based on their CV in the QCs, with a cut-off threshold of 30%.

We employed both univariate (UVDA) and multivariate (MVDA) data analysis methods to investigate the differences between the two *Mtb* infection time points (*Mtb*^*+*^*4w* and *Mtb*^*+*^*12w*) compared to the control group (*Mtb*^−^). To evaluate the lipid alterations among the three stages, we used Matlab (R2022a, MathWorks) to perform the Kruskal–Wallis test (*p* ≤ 0.05) after normality testing using the Shapiro-Wilk test. Subsequently, we conducted pairwise analyses using the Mann–Whitney *U* test to determine whether a specific lipid was significant in a comparison (*Mtb*^*+*^*4w vs Mtb*^−^; *Mtb*^*+*^*12w vs Mtb*^−^). Finally, the Benjamini–Hochberg correction test inspected the false discovery rate at level α = 0.05. Regarding MVDA, Pareto scaling was applied before generating the unsupervised principal component analysis (PCA-X), partial least square-discriminant analysis (PLS-DA), and orthogonal partial least square-discriminant analysis (OPLS-DA) models (SIMCA P + 17.0, Umetrics). The tight clustering of the QCs in the PCA plots (positive and negative ion modes) ensured the reliability and robustness of the analytical procedure (Supplementary Fig. 4a, b). PLS-DA was then performed to expose the global lipidomic changes caused by *Mtb* infection, and the groups were compared using the OPLS-DA model to maximize class discrimination and identify the underlying driving factors among the variables. The variable influence on projection (VIP) values were computed using the OPLS-DA models, selecting lipids with a VIP ≥ 1 and a jackknife confidence interval value other than zero. Finally, the OPLS-DA models were validated with cross-validation and the CV-ANOVA tool provided by SIMCA-P+ software.

### Reporting summary

Further information on research design is available in the Nature Portfolio Reporting Summary linked to this article.

### Supplementary information

Supplementary Information

Description of Additional Supplementary Files

Supplementary Data 1

Supplementary Data 2

Supplementary Data 3

Supplementary Data 4

Reporting Summary

## Data Availability

Data described in the manuscript is available to readers in Metabolomics Workbench Repository^55^ (Study ID ST002911), Project 10.21228/M8MQ7C. Lipidomics minimum reporting checklist^56^ 10.5281/zenodo.8413676.
