# Supplementary material for: LiLA: lipid lung-based ATLAS built through a comprehensive workflow designed for an accurate lipid annotation
Source: Commun Biol. 2024 Jan 5;7:45. doi: 10.1038/s42003-023-05680-7 (PMC10770321; doi:10.1038/s42003-023-05680-7)
Supplement: Supplementary file 1 — Supplementary Information [file 42003_2023_5680_MOESM1_ESM.pdf]

LiLA: Lipid Lung-based ATLAS built Through a Comprehensive Workflow Design for an Accurate Lipid Annotation

SUPPLEMENTARY INFORMATION

Belén Fernández Requena[1], Sajid Nadeem[2], Vineel P Reddy[2], Vanessa Naidoo[3], Joel N Glasgow[2], Adrie J C Steyn[2,3,4], Coral Barbas[1*], Carolina Gonzalez-Riano[1*]

[1]Centro de Metabolómica y Bioanálisis (CEMBIO), Facultad de Farmacia, Universidad San Pablo-CEU, CEU Universities, Urbanización Montepríncipe, 28660 Boadilla del Monte. España

[2] Department of Microbiology, University of Alabama at Birmingham, Birmingham, AL, United States

[3] Africa Health Research Institute, Durban, South Africa.

[4] Centers for AIDS Research and Free Radical Biology, University of Alabama at Birmingham, Birmingham, AL, United States.

*****Corresponding authors' e-mail: cbarbas@ceu.es, carolina.gonzalezriano@ceu.es

Supplementary Table 1. Description of the number of lipid annotations obtained in each step of this work.

	Number of lipids provided by the 4 software tools	*Number of lipids provided by the 4 software tools after removing redundant annotations*	*Number of well-annotated lipids after manual inspection*	*% of correctly annotated lipids by the software tools*
Cer	86	50	43	86
HexCer	6	6	5	83
SM	99	70	36	51
SPB	5	3	3	100
MG	9	9	9	100
DG	90	69	39	57
TG	358	336	336	100
LPC	61	35	35	100
LPE	20	13	13	100
LPI	1	1	1	100
LPS	1	1	1	100
LPG	1	1	1	100
PC	243	187	111	59
PE	150	93	74	80
PS	47	32	28	88
PG	59	22	22	100
PI	37	18	18	100
BMP	12	7	7	100
CL	2	2	2	100
FA	83	43	40	93
FAHFA	4	4	4	100
FA-OH	0	0	14	0
CAR	31	18	17	94
CE	4	2	2	100
Oxylipins	0	0	5	0
TOTAL	**1409**	**1022**	**866**	**84**

Supplementary Table 2. Description of the information regarding the percentage of false positives obtained from each one of the software tools employed in this study. The information in the first table summarizes those found in the second one. The lipids were considered “True positives” after the manual inspection checking supported by published works of Dr. Robert C. Murphy and Dr. Paul H. Axelsen, Dr. Maria Fedorova et al., and Dr. Xianlin Han.

Software	*Total*	*True positives*	*True positives (%)*	*False positives*	*False positives (%)*	*Based on total annotated lipids (866)*
Lipid Annotator	361	341	94.46	20	5.54	39.37
MS-Dial	637	480	75.35	157	24.65	55.42
LipidHunter	317	278	87.70	39	12.30	32.10
LipidMS	335	315	94.03	20	5.97	36.37

Lipid Class	*Total True positives*	*Lipid Annotator Total*	*Lipid Annotator True positives*	*MSDial Total*	*MSDial True positives*	*LipidHunter Total*	*LipidHunter True positives*	*LipidMS Total*	*LipidMS True positives*
BMP	7	5	5	7	7	–	–	–	–
Car	16	10	10	17	16	–	–	6	6
CE	2	1	1	2	2	–	–	1	1
Cer	43	45	38	38	28	10	9	5	5
HexCer	5	6	5	5	5	1	1	–	–
SM	36	32	32	56	23	64	27	16	16
CL	2	4	2	8	2	–	–	–	–
DG	39	15	15	55	25	33	27	18	14
FA	44	21	21	40	40	–	–	15	15
FAOH	14	–	–	–	–	–	–	–	–
Oxylipin	5	–	–	–	–	–	–	–	–
MG	9	–	–	–	–	–	–	9	9
LPC	35	20	20	18	18	–	–	23	23
LPE	13	7	7	3	3	4	4	6	6
LPS	1	–	–	1	1	–	–	1	1
LPI	1	–	–	–	–	1	1	1	1
LPG	1	–	–	1	1	1	1	–	–
PC	92	77	72	104	83	–	–	61	60
PC O-	19	–	–	40	19	–	–	37	19
PE	39	39	32	38	26	17	16	19	19
PE O-	36	–	–	45	22	15	11	17	17
PG	22	19	19	17	17	14	14	9	8
PI	18	8	8	13	13	10	10	6	6
PS	28	14	14	20	16	12	12	5	5
SPB	3	–	–	3	3	–	–	2	2
TG	336	38	38	106	102	135	129	78	77
Total	**866**	**361**	**341**	**637**	**480**	**317**	**278**	**335**	**315**

Supplementary Table 3. Splash® Lipidomix (SL) Internal Standard mixture used for lipid-class-specific semi-quantification including the concentration range of each lipid species (C, concentration).

Lipid	***m/z***	***RT (min)***	***SL (ppm)***	***C1 (ppm)***	***C2 (ppm)***	***C3 (ppm)***	***C4 (ppm)***	***C5 (ppm)***	***C6 (ppm)***
15:0-18:1(d7) PC	753.613	7.917	150.6	0.015	0.034	0.10	5.8	20.8	75.3
18:1(d7) Lyso PC	529.399	2.628	23.8	0.0024	0.0051	0.016	0.92	3.3	11.9
15:0-18:1(d7) PE	711.566	9.098	5.3	0.00054	0.0011	0.0036	0.20	0.73	2.7
18:1(d7) Lyso PE	487.352	2.734	4.9	0.00050	0.0011	0.0034	0.19	0.68	2.5
15:0-18:1(d7) PG	742.561	6.434	26.7	0.0027	0.0058	0.018	1.0	3.7	13.4
15:0-18:1(d7) PI	830.577	6.303	8.5	0.00086	0.0018	0.0058	0.33	1.2	4.3
15:0-18:1(d7) PS	755.556	5.668	4.2	0.00043	0.0009	0.0029	0.16	0.58	2.1
15:0-18:1(d7)-15:0 TAG	812.772	14.101	52.8	0.0054	0.011	0.036	2.0	7.3	26.4
15:0-18:1(d7) DAG	588.558	11.733	8.8	0.00089	0.0019	0.0061	0.34	1.2	4.4
18:1(d7) MAG	364.344	3.617	1.8	0.00018	0.00039	0.0012	0.069	0.25	0.90
18:1(d7) Chol Ester	658.651	15.018	329.1	0.033	0.071	0.23	12.7	45.4	164.6
d18:1-18:1(d9) SM	738.647	7.117	29.6	0.0030	0.0064	0.020	1.1	4.1	14.8
15:0-18:1(d7) PA	668.525	7.236	6.9	0.00070	0.0015	0.0047	0.27	0.95	3.5
Cholesterol-d7	394.406	7.368	98.4	0.010	0.021	0.068	3.8	13.6	49.2

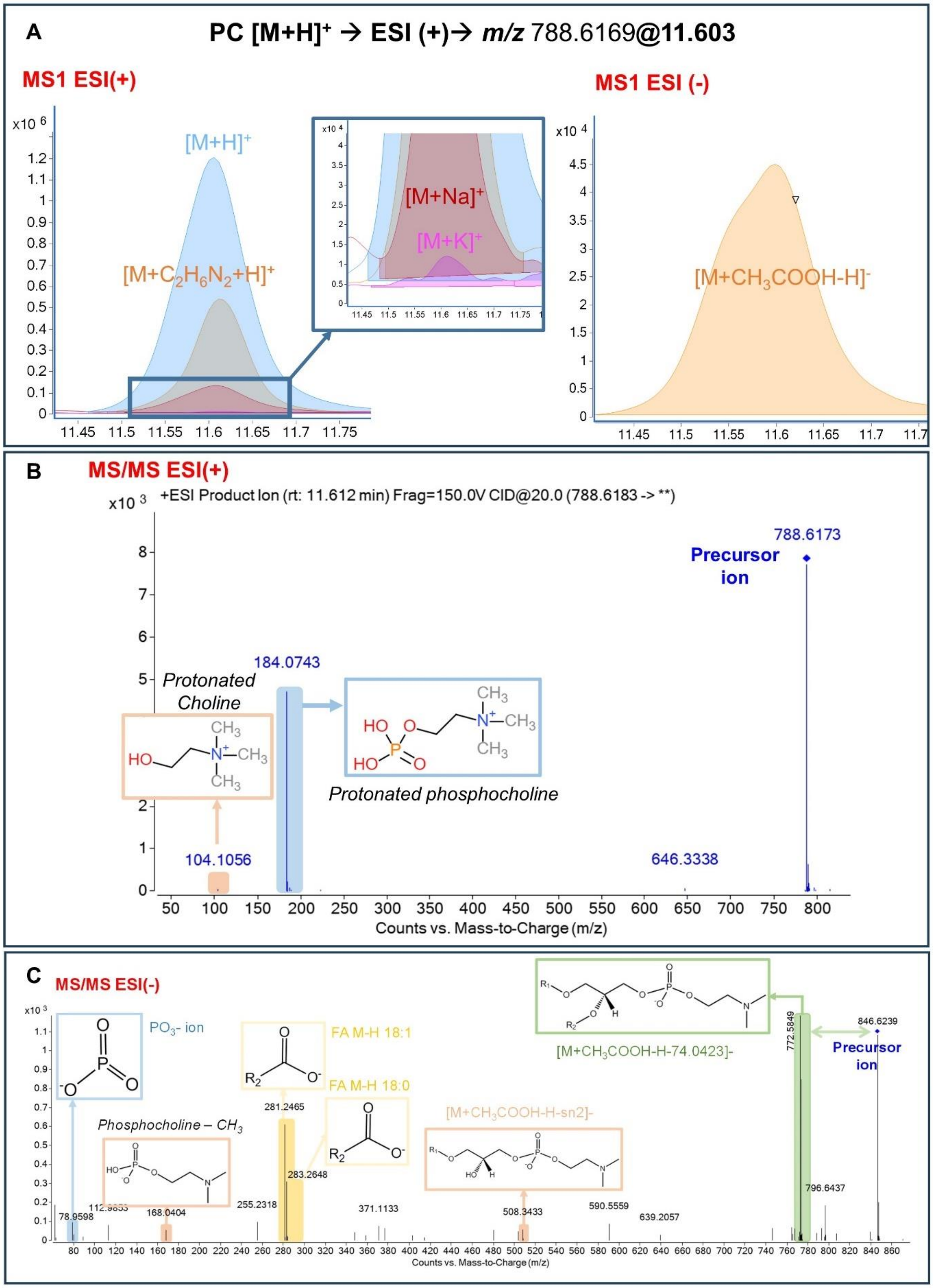

Supplementary Fig.1. Example of the PC (18:0/18:1) manual inspection contracted by the state of the art of lipidomics to date. **(A)** Extracted Ion Chromatogram (EIC) to obtain the adduct pattern formation of the

lipid species that we are interested in by using the *m/z* and the RT. **(B)** MS2 (MS/MS) ESI(+) data files were used to determine the lipid class and subclass. **(C)** MS2 (MS/MS) ESI(-) data files were used to determine the fatty acyl-chains, the number of unsaturations and, based on the intensity rules, the acyl-chain position.

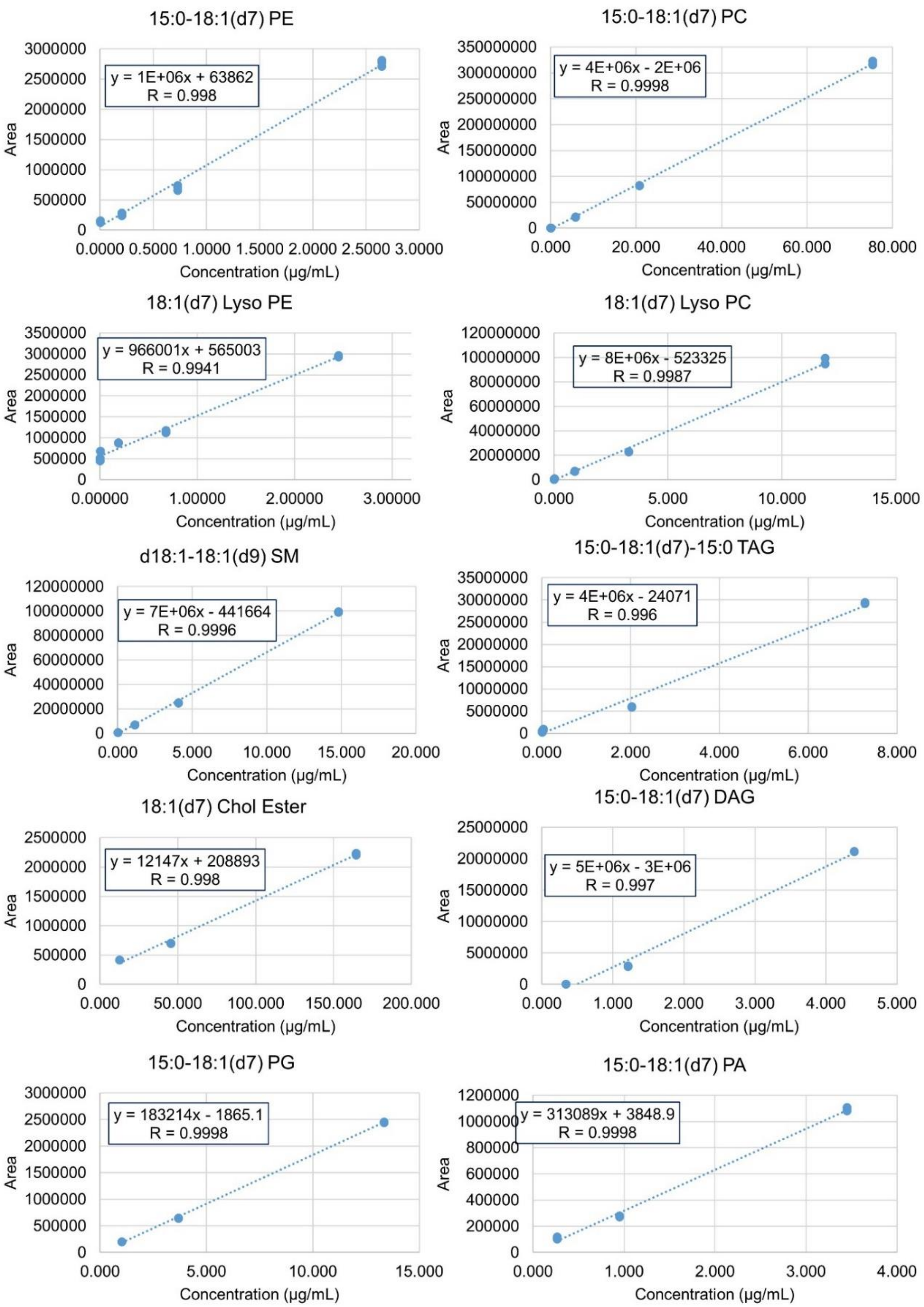

15:0-18:1(d7) PE
y = 1E+06x + 63862
R = 0.998
Area
Concentration (µg/mL)
15:0-18:1(d7) PC
y = 4E+06x - 2E+06
R = 0.9998
Area
Concentration (µg/mL)
18:1(d7) Lyso PE
y = 966001x + 565003
R = 0.9941
Area
Concentration (µg/mL)
18:1(d7) Lyso PC
y = 8E+06x - 523325
R = 0.9987
Area
Concentration (µg/mL)
d18:1-18:1(d9) SM
y = 7E+06x - 441664
R = 0.9996
Area
Concentration (µg/mL)
15:0-18:1(d7)-15:0 TAG
y = 4E+06x - 24071
R = 0.996
Area
Concentration (µg/mL)
18:1(d7) Chol Ester
y = 12147x + 208893
R = 0.998
Area
Concentration (µg/mL)
15:0-18:1(d7) DAG
y = 5E+06x - 3E+06
R = 0.997
Area
Concentration (µg/mL)
15:0-18:1(d7) PG
y = 183214x - 1865.1
R = 0.9998
Area
Concentration (µg/mL)
15:0-18:1(d7) PA
y = 313089x + 3848.9
R = 0.9998
Area
Concentration (µg/mL)

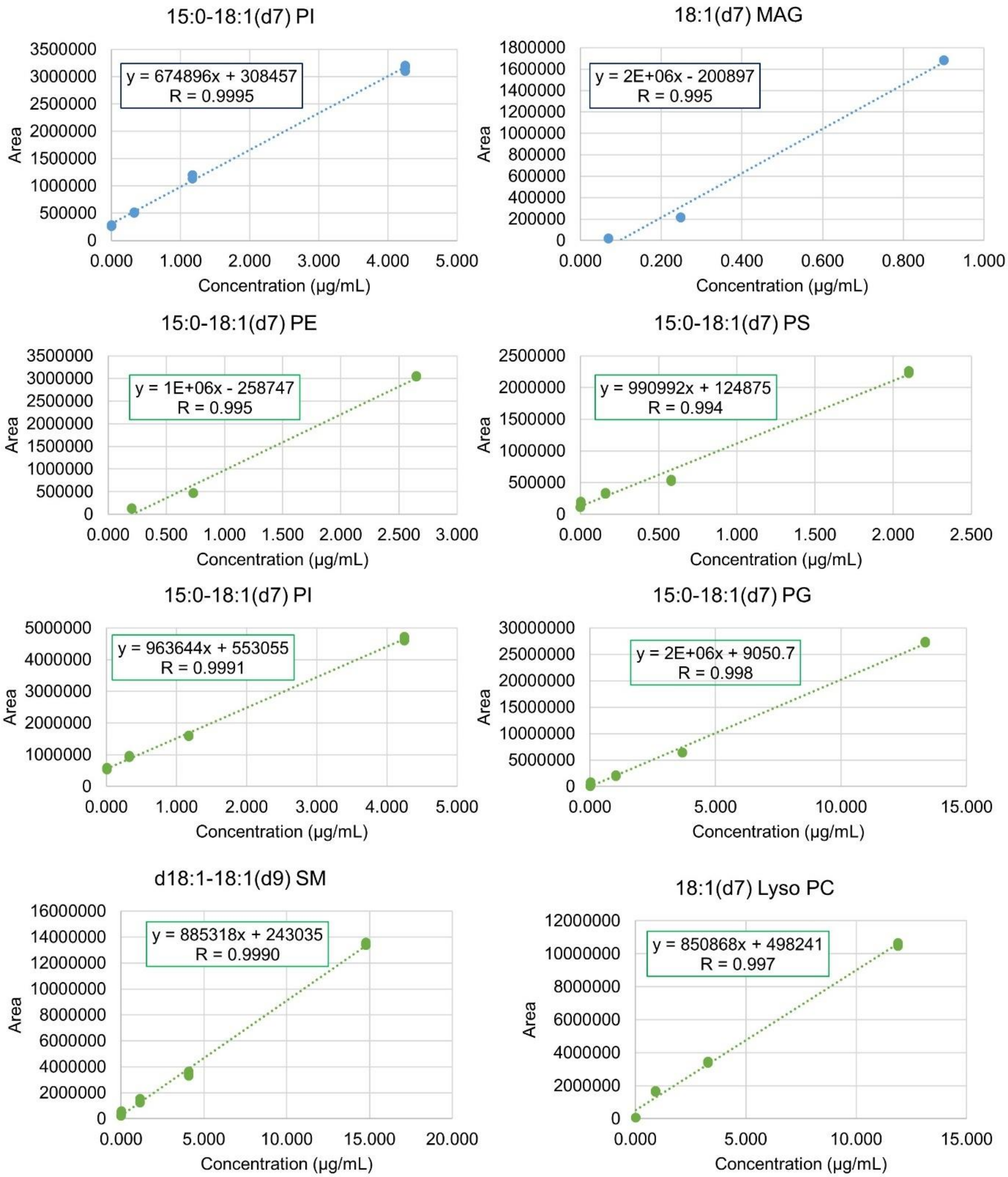

Supplementary Fig.2. Internal calibration curves of spiked internal standards into pooled mice lung sample (ESI(+) – blue; ESI(–) – green). Only calibration points resulting in a linear regression R > 0.990 were used for calibration curve generation.

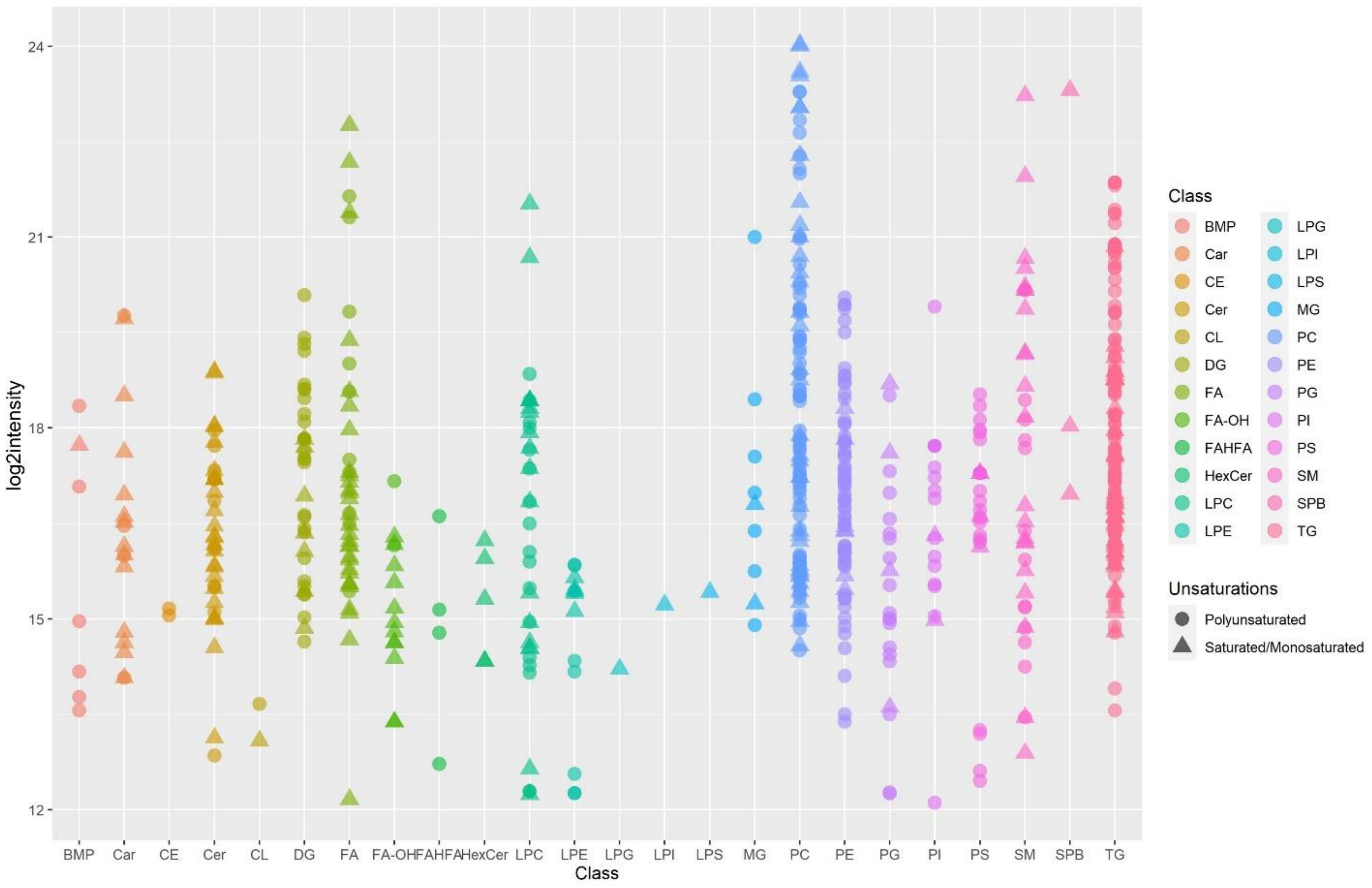

Supplementary Fig.3. Graphical representation of the lipid classes based on the lipid species intensity. Lipids are arranged alphabetically. The triangle represents saturated/monounsaturated lipids, whereas the circle indicates polyunsaturated lipids per class.

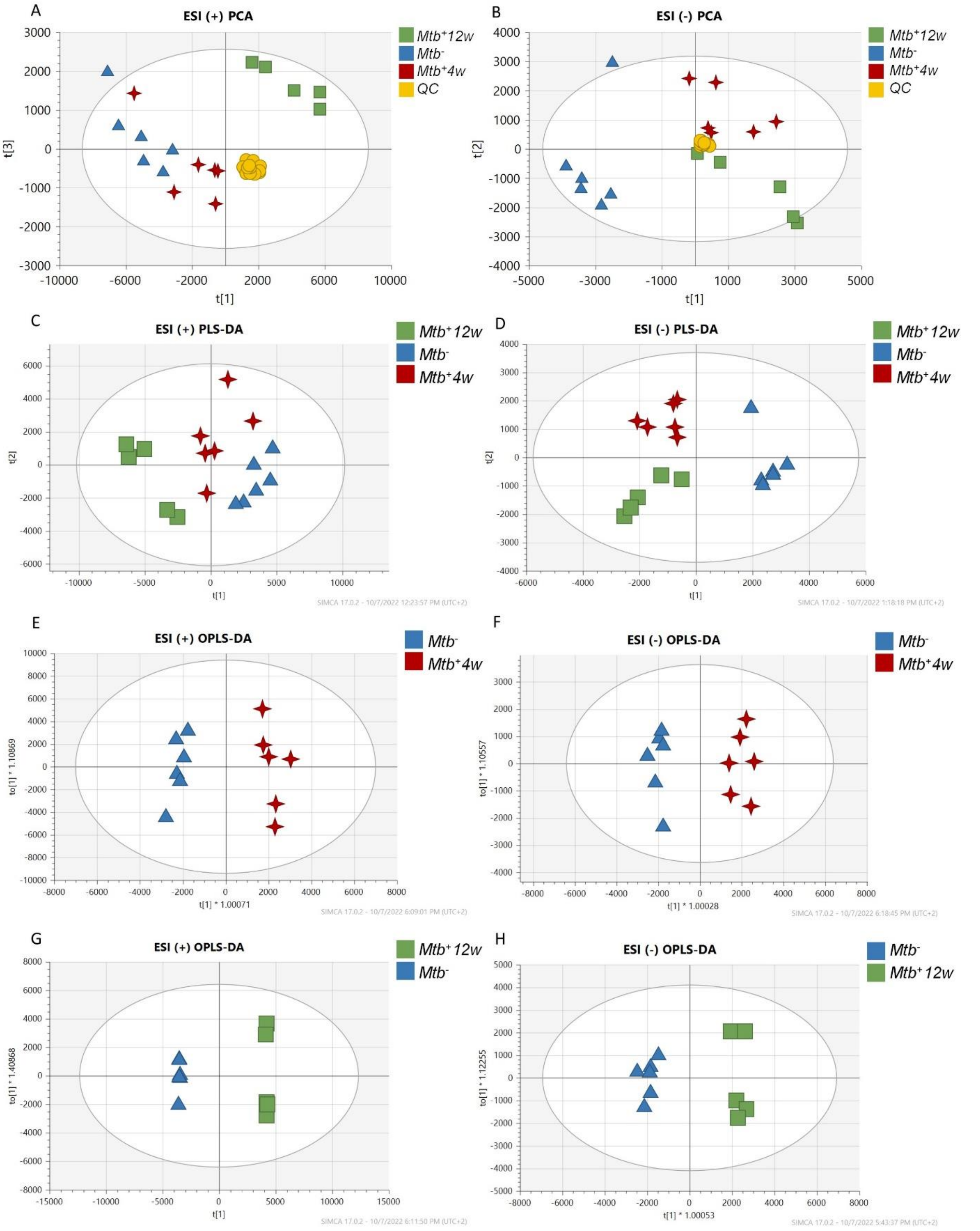

Supplementary Fig.4. Multivariate plots for statistical analysis. *Mtb*$^{-}$ (blue) corresponds to healthy mice group; *Mtb*$^{+}$*4w* (red) is the four weeks post infection group; and *Mtb*$^{+}$*12w* (green) is the twelve weeks post infection group. Plots **(A)** and **(B)** represent the PCA score plots obtained for ESI(+) and ESI(-) data,

respectively. As can be observed, the QCs were tightly clustered ensuring the stability and robustness of the analytical performance. **(C)** and **(D)** correspond to supervised PLS-DA plots analysis for both ionization modes with their corresponding p-value and quality parameters, indicating the power of the models. **(C)** has a p-value of 0.00021, an R^2 of 0.970 and Q^2 of 0.920. **(D)** has a p-value of 0.00021, an R^2 of 0.962 and Q^2 of 0.879. **(E)** and **(F)** are the supervised OPLS-DA plots, in both ionization modes, for the evaluation by pairs between healthy control mice group and four weeks post infection mice group. Plot **(E)** has a p-value of 0.033, an R^2 of 0.969 and Q^2 of 0.880. **(F)** has a p-value of 0.0024, an R^2 of 0.966 and Q^2 of 0.880. **(G)** and **(H)** represent the supervised OPLS-DA, in both ionization modes, for the evaluation of pairs between healthy control mice group and twelve weeks post infection group. **(G)** has a p-value of 0.054, an R^2 of 0.992 and Q^2 of 0.986. **(H)** has a p-value of 0.00058, an R^2 of 0.981 and Q^2 of 0.947.
