# Supplementary material for: LiLA: lipid lung-based ATLAS built through a comprehensive workflow designed for an accurate lipid annotation
Source: Commun Biol. 2024 Jan 5;7:45. doi: 10.1038/s42003-023-05680-7 (PMC10770321; doi:10.1038/s42003-023-05680-7)
Supplement: Supplementary file 2 — Description of Additional Supplementary Files [file 42003_2023_5680_MOESM2_ESM.pdf]

Description of Additional Supplementary Files

File name: Supplementary Data 1

Description: 866 lipid molecular species accurately annotated using the proposed workflow

File name: Supplementary Data 2

Description: Data employed for creating the graphic charts depicted in Figures 5 and 6.

File name: Supplementary Data 3

Description: Semi-quantification results of the semi-targeted experiment.

File name: Supplementary Data 4

Description: Lipid alterations detected after comparing Mtb+4w vs Mtb- and Mtb+12w vs Mtb-.
