# Supplementary material for: LiLA: lipid lung-based ATLAS built through a comprehensive workflow designed for an accurate lipid annotation
Source: Commun Biol. 2024 Jan 5;7:45. doi: 10.1038/s42003-023-05680-7 (PMC10770321; doi:10.1038/s42003-023-05680-7)
Supplement: Supplementary file 3 — Supplementary Data 1 [file 42003_2023_5680_MOESM3_ESM.pdf]

Supplementary Data 1. 866 lipid molecular species accurately annotated using the proposed workflow.

RT	m/z	Sum Composition	Name	Adduct	Formula	Neutral Mass	Confidence level
12.140	692.6187	Cer 41;2;O2	Cer 15:0;O2/26:2	[M+CH3COO]-	C41H79NO3	633.6058	2
9.024	536.5050	Cer 34:1;O2	Cer 16:0;O2/18:1	[M-H]-	C34H67NO3	537.5122	2
7.600	594.5108	Cer 34:2;O2	Cer 16:0;O2/18:2	[M+CH3COO]-	C34H65NO3	535.4948	2
12.130	666.6042	Cer 39:1;O2	Cer 16:0;O2/23:1	[M+CH3COO]-	C39H77NO3	607.5903	2
10.074	540.5342	Cer 34:0;O2	Cer 18:0;O2/16:0	[M+H]+	C34H69NO3	539.5270	2
11.704	626.5723	Cer 36:0;O2	Cer 18:0;O2/18:0	[M+CH3COO]-	C36H73NO3	567.5591	2
11.490	624.5564	Cer 36:1;O2	Cer 18:0;O2/18:1	[M+CH3COO]-	C36H71NO3	565.5432	2
12.087	654.6029	Cer 38:0;O2	Cer 18:0;O2/20:0	[M+CH3COO]-	C38H77NO3	595.5897	2
12.400	624.6218	Cer 40:0;O2	Cer 18:0;O2/22:0	[M+H]+	C40H81NO3	623.6146	2
12.536	696.6500	Cer 41:0;O2	Cer 18:0;O2/23:0	[M+CH3COO]-	C41H83NO3	637.6368	2
12.730	652.6602	Cer 42:0;O2	Cer 18:0;O2/24:0	[M+H]+	C42H85NO3	651.6530	2
12.369	708.6514	Cer 42:1;O2	Cer 18:0;O2/24:1	[M+CH3COO]-	C42H83NO3	649.6382	2
12.890	720.6502	Cer 43:0;O2	Cer 18:0;O2/25:0	[M+CH3COO]-	C43H87NO3	661.6373	2
12.740	722.6658	Cer 43:1;O2	Cer 18:0;O2/25:1	[M+CH3COO]-	C43H85NO3	663.6526	2
12.910	736.6802	Cer 44:1;O2	Cer 18:0;O2/26:1	[M+CH3COO]-	C44H87NO3	677.6673	2
7.280	568.4936	Cer 32:1;O2	Cer 18:1;O2/14:0	[M+CH3COO]-	C32H63NO3	509.4807	2
8.170	524.5016	Cer 33:1;O2	Cer 18:1;O2/15:0	[M+H]+	C33H65NO3	523.4944	2
9.232	538.5197	Cer 34:1;O2	Cer 18:1;O2/16:0	[M+H]+	C34H67NO3	537.5125	2
10.420	610.5404	Cer 35:1;O2	Cer 18:1;O2/17:0	[M+CH3COO]-	C35H69NO3	551.5272	2
11.509	566.5504	Cer 36:1;O2	Cer 18:1;O2/18:0	[M+H]+	C36H71NO3	565.5432	2
11.801	638.5732	Cer 37:1;O2	Cer 18:1;O2/19:0	[M+CH3COO]-	C37H73NO3	579.5600	2
11.987	594.5810	Cer 38:1;O2	Cer 18:1;O2/20:0	[M+H]+	C38H75NO3	593.5738	2
12.132	666.6036	Cer 39:1;O2	Cer 18:1;O2/21:0	[M+CH3COO]-	C39H75NO3	607.5904	2
12.273	622.6141	Cer 40:1;O2	Cer 18:1;O2/22:0	[M+H]+	C40H79NO3	621.6069	2
12.433	636.6304	Cer 41:1;O2	Cer 18:1;O2/23:0	[M+H]+	C41H81NO3	635.6232	2
12.586	650.6451	Cer 42:1;O2	Cer 18:1;O2/24:0	[M+H]+	C42H83NO3	649.6379	2
12.268	648.6295	Cer 42:2;O2	Cer 18:1;O2/24:1	[M+H]+	C42H81NO3	647.6223	2
12.043	646.6142	Cer 42:3;O2	Cer 18:1;O2/24:2	[M+H]+	C42H79NO3	645.6070	2
12.714	722.6670	Cer 43:1;O2	Cer 18:1;O2/25:0	[M+CH3COO]-	C43H85NO3	663.6510	2
12.426	720.6484	Cer 43:2;O2	Cer 18:1;O2/25:1	[M+CH3COO]-	C43H83NO3	661.6324	2
12.879	736.6827	Cer 44:1;O2	Cer 18:1;O2/26:0	[M+CH3COO]-	C44H87NO3	677.6667	2
7.595	536.5037	Cer 34:2;O2	Cer 18:2;O2/16:0	[M+H]+	C34H65NO3	535.4965	2
9.720	564.5339	Cer 36:2;O2	Cer 18:2;O2/18:0	[M+H]+	C36H69NO3	563.5267	2
11.625	650.5730	Cer 38:2;O2	Cer 18:2;O2/20:0	[M+CH3COO]-	C38H73NO3	591.5598	2
12.035	620.5976	Cer 40:2;O2	Cer 18:2;O2/22:0	[M+H]+	C40H77NO3	619.5904	2
12.184	634.6119	Cer 41:2;O2	Cer 18:2;O2/23:0	[M+H]+	C41H79NO3	633.6047	2
12.270	632.6346	Cer 42:2;O2	Cer 18:2;O2/24:0	[M+H]+	C42H81NO3	631.6274	2
12.042	646.6126	Cer 42:3;O2	Cer 18:2;O2/24:1	[M+H]+	C42H79NO3	645.6054	2

11.763	702.6044	Cer 42:4;O2	Cer 18:2;O2/24:2	[M+CH3COO]-	C42H77NO3	643.5912	2
12.414	720.6501	Cer 43:2;O2	Cer 18:2;O2/25:0	[M+CH3COO]-	C43H81NO3	661.6341	2
12.567	732.6613	Cer 44:3;O2	Cer 18:2;O2/26:1	[M+CH3COO]-	C44H83NO3	673.6453	2
12.420	720.6501	Cer 43:2;O2	Cer 19:0;O2/24:2	[M+CH3COO]-	C43H81NO3	661.6341	2
11.468	624.5582	Cer 36:1;O2	Cer 19:1;O2/17:0	[M+CH3COO]-	C36H69NO3	565.5510	2
7.515	758.5792	HexCer 34:1;O2	HexCer 18:0;O2/22:1	[M+CH3COO]-	C40H77NO8	699.5649	2
11.983	842.6724	HexCer 40:1;O2	HexCer 18:1;O2/16:0	[M+CH3COO]-	C46H89NO8	783.6588	2
12.012	842.6724	HexCer 40:1;O2	HexCer 18:1;O2/22:0	[M+CH3COO]-	C46H89NO8	783.6588	2
12.224	870.7040	HexCer 42:1;O2	HexCer 18:1;O2/24:0	[M+CH3COO]-	C48H93NO8	811.6901	2
11.977	868.6875	HexCer 42:2;O2	HexCer 18:1;O2/24:1	[M+CH3COO]-	C48H91NO8	809.6745	2
7.845	705.5903	SM 34:0;O2	SM 18:0;O2/16:0	[M+H]+	C39H81O6PN2	704.5831	2
10.236	733.6227	SM 36:0;O2	SM 18:0;O2/18:0	[M+H]+	C41H85O6PN2	732.6155	2
11.781	761.6536	SM 38:0;O2	SM 18:0;O2/20:0	[M+H]+	C43H89O6PN2	760.6464	2
12.128	789.6831	SM 40:0;O2	SM 18:0;O2/22:0	[M+H]+	C45H93O6PN2	788.6759	2
11.470	783.6375	SM 40:3;O2	SM 18:0;O2/22:3	[M+H]+	C45H87O6PN2	782.6303	2
12.047	839.7054	SM 42:0;O2	SM 18:0;O2/24:0	[M+Na]+	C47H97O6PN2	816.7084	2
12.100	815.6997	SM 42:1;O2	SM 18:0;O2/24:1	[M+H]+	C47H95O6PN2	814.6925	2
5.939	675.5413	SM 32:1;O2	SM 18:1;O2/14:0	[M+H]+	C37H75O6PN2	674.5341	2
6.560	689.5585	SM 33:1;O2	SM 18:1;O2/15:0	[M+H]+	C38H77O6PN2	688.5513	2
7.305	703.5764	SM 34:1;O2	SM 18:1;O2/16:0	[M+H]+	C39H79O6PN2	702.5692	2
8.189	717.5872	SM 35:1;O2	SM 18:1;O2/17:0	[M+H]+	C40H81O6PN2	716.5800	2
9.209	731.6066	SM 36:1;O2	SM 18:1;O2/18:0	[M+H]+	C41H83O6PN2	730.5994	2
10.450	745.6224	SM 37:1;O2	SM 18:1;O2/19:0	[M+H]+	C42H85O6PN2	744.6152	2
11.480	759.6376	SM 38:1;O2	SM 18:1;O2/20:0	[M+H]+	C43H87O6PN2	758.6304	2
11.819	773.6535	SM 39:1;O2	SM 18:1;O2/21:0	[M+H]+	C44H89O6PN2	772.6463	2
11.978	787.6682	SM 40:1;O2	SM 18:1;O2/22:0	[M+H]+	C45H91O6PN2	786.6610	2
12.139	801.6847	SM 41:1;O2	SM 18:1;O2/23:0	[M+H]+	C46H93O6PN2	800.6775	2
12.307	815.6995	SM 42:1;O2	SM 18:1;O2/24:0	[M+H]+	C47H95O6PN2	814.6923	2
11.977	813.6842	SM 42:2;O2	SM 18:1;O2/24:1	[M+H]+	C47H93O6PN2	812.6770	2
11.625	869.6754	SM 42:3;O2	SM 18:1;O2/24:2	[M+CH3COO]-	C47H91O6PN2	810.6615	2
12.450	829.7162	SM 43:1;O2	SM 18:1;O2/25:0	[M+H]+	C48H97O6PN2	828.7090	2
12.278	841.7229	SM 44:2;O2	SM 18:1;O2/26:1	[M+H]+	C49H97O6PN2	840.7157	2
6.198	701.5595	SM 34:2;O2	SM 18:2;O2/16:0	[M+H]+	C39H77O6PN2	700.5523	2
7.610	729.5884	SM 36:2;O2	SM 18:2;O2/18:0	[M+H]+	C41H81O6PN2	728.5812	2
9.726	757.6226	SM 38:2;O2	SM 18:2;O2/20:0	[M+H]+	C43H85O6PN2	756.6154	2
11.630	785.6541	SM 40:2;O2	SM 18:2;O2/22:0	[M+H]+	C45H89O6PN2	784.6469	2
9.860	783.6375	SM 40:3;O2	SM 18:2;O2/22:1	[M+H]+	C45H87O6PN2	782.6303	2
11.821	857.6741	SM 41:2;O2	SM 18:2;O2/23:0	[M+CH3COO]-	C46H91O6PN2	798.6614	2
11.850	813.6832	SM 42:2;O2	SM 18:2;O2/24:0	[M+H]+	C47H93O6PN2	812.6760	2
12.147	827.6992	SM 43:2;O2	SM 18:2;O2/25:0	[M+H]+	C48H95O6PN2	826.6920	2
12.150	801.6865	SM 41:1;O2	SM 19:1;O2/22:0	[M+H]+	C46H93O6PN2	800.6793	2

11.882	857.6749	SM 41:2;O2	SM 24:1;O2/17:1	[M+CH3COO]-	C46H91O6PN2	798.6614	2
12.270	803.7000	SM 41:0;O2	SM 41:0;O2	[M+H]+	C47H89O6PN2	802.6928	4
10.385	809.6487	SM 42:4;O2	SM 42:4;O2	[M+H]+	C49H91O6PN2	808.6415	4
11.942	835.6693	SM 44:5;O2	SM 44:5;O2	[M+H]+	C56H115O6PN2	834.6621	4
12.324	943.8571	SM 51:0;O2	SM 51:0;O2	[M+H]+	C46H95O6PN2	942.8499	4
2.111	302.3059	SPB 18:0;O2	SPB 18:0;O2	[M+H]+	C18H39NO2	301.2981	2
2.420	300.2903	SPB 18:1;O2	SPB 18:1;O2	[M+H]+	C18H37NO2	299.2824	2
3.733	379.2824	MG 18:1	MG 18:1	[M+Na]+	C21H40O4	356.3024	2
1.604	372.3109	MG 18:2	MG 18:2	[M+NH4]+	C21H38O4	355.2844	2
2.331	402.3563	MG 20:1	MG 20:1	[M+NH4]+	C23H44O4	385.3298	2
2.375	400.3441	MG 20:2	MG 20:2	[M+NH4]+	C23H4O24	383.3176	2
1.766	398.3272	MG 20:3	MG 20:3	[M+NH4]+	C23H40O4	381.3007	2
3.079	401.2673	MG 20:4	MG 20:4	[M+Na]+	C23H38O4	378.2873	2
2.562	426.3591	MG 22:3	MG 22:3	[M+NH4]+	C25H44O4	409.3326	2
4.167	426.3593	MG 22:3	MG 22:3	[M+NH4]+	C25H44O4	409.3328	2
2.973	425.2662	MG 22:6	MG 22:6	[M+Na]+	C25H38O4	402.2862	2
11.456	584.5254	DG 32:1	DG 14:0_18:1	[M+NH4]+	C35H66O5	566.4910	3
11.935	586.5404	DG 32:0	DG 16:0/16:0	[M+NH4]+	C35H68O5	568.5067	2
11.498	584.5248	DG 32:1	DG 16:0_16:1	[M+NH4]+	C35H66O5	566.4910	3
11.965	612.5571	DG 34:1	DG 16:0_18:1	[M+NH4]+	C37H70O5	594.5223	3
11.674	610.5413	DG 34:2	DG 16:0_18:2	[M+NH4]+	C37H68O5	592.5067	3
11.060	608.5253	DG 34:3	DG 16:0_18:3	[M+NH4]+	C37H66O5	590.4910	3
11.819	636.5567	DG 36:3	DG 16:0_20:3	[M+NH4]+	C39H70O5	618.5223	3
11.569	634.5409	DG 36:4	DG 16:0_20:4	[M+NH4]+	C39H68O5	616.5067	3
11.862	662.5724	DG 38:4	DG 16:0_22:4	[M+NH4]+	C41H7O25	644.5380	3
11.649	660.5572	DG 38:5	DG 16:0_22:5	[M+NH4]+	C41H70O5	642.5223	3
11.345	658.5380	DG 38:6	DG 16:0_22:6	[M+NH4]+	C41H68O5	640.5067	3
11.513	584.5240	DG 32:1	DG 16:1_16:0	[M+NH4]+	C35H66O5	566.4910	3
11.655	610.5410	DG 34:2	DG 16:1_18:1	[M+NH4]+	C37H68O5	592.5067	3
10.265	608.5247	DG 34:3	DG 16:1_18:2	[M+NH4]+	C37H66O5	590.4910	3
11.830	648.5571	DG 37:4	DG 17:0_20:4	[M+NH4]+	C40H70O5	630.5223	3
12.248	640.5892	DG 36:1	DG 18:0_18:1	[M+NH4]+	C39H74O5	620.5380	3
12.010	638.5728	DG 36:2	DG 18:0_18:2	[M+NH4]+	C39H7O25	620.5380	3
12.105	664.5894	DG 38:3	DG 18:0_20:3	[M+NH4]+	C41H74O5	646.5536	3
11.984	662.5728	DG 38:4	DG 18:0_20:4	[M+NH4]+	C41H7O25	644.5380	3
12.179	690.6050	DG 40:4	DG 18:0_22:4	[M+NH4]+	C43H76O5	672.5693	3
12.006	688.5921	DG 40:5	DG 18:0_22:5	[M+NH4]+	C43H74O5	672.5693	3
11.914	686.5738	DG 40:6	DG 18:0_22:6	[M+NH4]+	C43H7O25	668.5380	3
11.973	612.5580	DG 34:1	DG 18:1_16:0	[M+NH4]+	C37H70O5	594.5223	3
12.021	638.5726	DG 36:2	DG 18:1/18:1	[M+NH4]+	C39H7O25	620.5380	2
11.737	636.5569	DG 36:3	DG 18:1_18:2	[M+NH4]+	C39H70O5	618.5223	3

10.915	634.5410	DG 36:4	DG 18:1_18:3	[M+NH4]+	C39H68O5	616.5067	3
11.850	662.5742	DG 38:4	DG 18:1_20:3	[M+NH4]+	C41H7O25	644.5380	3
11.641	660.5542	DG 38:5	DG 18:1_20:4	[M+NH4]+	C41H70O5	642.5223	3
11.937	688.5880	DG 40:5	DG 18:1_22:4	[M+NH4]+	C43H74O5	672.5693	3
11.488	684.5570	DG 40:7	DG 18:1_22:6	[M+NH4]+	C43H70O5	666.5223	3
11.674	610.5430	DG 34:2	DG 18:2_16:0	[M+NH4]+	C37H68O5	592.5067	3
12.013	638.5739	DG 36:2	DG 18:2_18:0	[M+NH4]+	C39H7O25	620.5380	3
11.750	636.5577	DG 36:3	DG 18:2_18:1	[M+NH4]+	C39H70O5	619.5223	3
10.963	634.5407	DG 36:4	DG 18:2/18:2	[M+NH4]+	C39H68O5	616.5067	2
9.475	632.5281	DG 36:5	DG 18:2_18:3	[M+NH4]+	C39H66O5	614.4910	3
9.912	682.5398	DG 40:8	DG 18:2_22:6	[M+NH4]+	C43H68O5	664.5067	3
12.028	688.5880	DG 40:5	DG 20:1_20:4	[M+NH4]+	C43H74O5	672.5693	3
9.982	682.5410	DG 40:8	DG 20:4_20:4	[M+NH4]+	C43H68O5	664.5067	3
11.972	688.5866	DG 40:5	DG 22:4_18:1	[M+NH4]+	C43H74O5	672.5693	3
6.353	516.4264	TG 26:0	TG 10:0_8:0_8:0	[M+NH4]+	C29H54O6	498.3920	3
6.324	516.4258	TG 26:0	TG 8:0_8:0_10:0	[M+NH4]+	C29H54O6	498.3920	3
11.864	600.5203	TG 32:0	TG 12:0_14:0_6:0	[M+NH4]+	C35H66O6	582.4859	3
12.428	656.5845	TG 36:0	TG 10:0_18:0_8:0	[M+NH4]+	C39H74O6	638.5485	3
12.428	656.5845	TG 36:0	TG 12:0_10:0_14:0	[M+NH4]+	C39H74O6	638.5485	3
12.442	656.5829	TG 36:0	TG 12:0/12:0/12:0	[M+NH4]+	C39H74O6	638.5485	2
12.428	656.5829	TG 36:0	TG 16:0_10:0_10:0	[M+NH4]+	C39H74O6	638.5485	3
12.428	656.5829	TG 36:0	TG 16:0_16:0_4:0	[M+NH4]+	C39H74O6	638.5485	3
12.428	656.5829	TG 36:0	TG 16:0_4:0_16:0	[M+NH4]+	C39H74O6	638.5485	3
12.428	656.5845	TG 36:0	TG 16:0_8:0_12:0	[M+NH4]+	C39H74O6	638.5485	3
12.471	682.5986	TG 38:1	TG 16:0_4:0_18:1	[M+NH4]+	C41H76O6	664.5642	3
12.471	682.5986	TG 38:1	TG 18:1_16:0_4:0	[M+NH4]+	C41H76O6	664.5642	3
12.471	682.5986	TG 38:1	TG 18:1_4:0_16:0	[M+NH4]+	C41H76O6	664.5642	3
12.471	682.5986	TG 38:1	TG 4:0_18:0_16:1	[M+NH4]+	C41H76O6	664.5642	3
12.964	712.6454	TG 40:0	TG 10:0_14:0_16:0	[M+NH4]+	C43H8O26	694.6189	3
12.471	708.6143	TG 40:2	TG 16:1_8:0_16:1	[M+NH4]+	C43H78O6	690.5798	3
12.471	708.6142	TG 40:2	TG 18:1_16:1_6:0	[M+NH4]+	C43H78O6	690.5798	3
12.471	708.6142	TG 40:2	TG 18:1_18:1_4:0	[M+NH4]+	C43H78O6	690.5798	3
12.471	708.6142	TG 40:2	TG 6:0_18:2_16:0	[M+NH4]+	C43H78O6	690.5798	3
13.359	740.6761	TG 42:0	TG 10:0_14:0_18:0	[M+NH4]+	C45H86O6	722.6424	3
13.393	740.6768	TG 42:0	TG 10:0_16:0_16:0	[M+NH4]+	C45H86O6	722.6424	3
13.393	740.6768	TG 42:0	TG 12:0_12:0_18:0	[M+NH4]+	C45H86O6	722.6424	3
13.393	740.6768	TG 42:0	TG 12:0_14:0_16:0	[M+NH4]+	C45H86O6	722.6424	3
13.393	740.6768	TG 42:0	TG 14:0/14:0/14:0	[M+NH4]+	C45H86O6	722.6424	2
13.771	768.7075	TG 44:0	TG 14:0_16:0_14:0	[M+NH4]+	C47H90O6	750.6737	3
13.780	768.7087	TG 44:0	TG 14:0_12:0_18:0	[M+NH4]+	C47H90O6	750.6737	3
13.727	768.7082	TG 44:0	TG 14:0_14:0_16:0	[M+NH4]+	C47H90O6	750.6737	3

13.809	768.7087	TG 44:0	TG 14:0_18:0_12:0	[M+NH4]+	C47H90O6	750.6737	3
13.777	768.7088	TG 44:0	TG 16:0_12:0_16:0	[M+NH4]+	C47H90O6	750.6737	3
13.780	768.7082	TG 44:0	TG 16:0_14:0_14:0	[M+NH4]+	C47H90O6	750.6737	3
13.355	766.6926	TG 44:1	TG 14:0_12:0_18:1	[M+NH4]+	C47H88O6	748.6581	3
13.355	766.6925	TG 44:1	TG 14:0_14:0_16:1	[M+NH4]+	C47H88O6	748.6581	3
13.355	766.6879	TG 44:1	TG 14:0_14:1_16:0	[M+NH4]+	C47H88O6	748.6581	3
13.355	766.6925	TG 44:1	TG 14:0_16:1_14:0	[M+NH4]+	C47H88O6	748.6581	3
13.355	766.6926	TG 44:1	TG 16:0_10:0_18:1	[M+NH4]+	C47H88O6	748.6581	3
13.355	766.6906	TG 44:1	TG 16:0_12:0_16:1	[M+NH4]+	C47H88O6	748.6581	3
13.355	766.6879	TG 44:1	TG 16:0_14:1_14:0	[M+NH4]+	C47H88O6	748.6581	3
13.403	766.6929	TG 44:1	TG 18:0_16:1_10:0	[M+NH4]+	C47H88O6	748.6581	3
14.277	796.7394	TG 46:0	TG 14:0_14:0_18:0	[M+NH4]+	C49H94O6	778.7050	3
14.260	796.7390	TG 46:0	TG 14:0_16:0_16:0	[M+NH4]+	C49H94O6	778.7050	3
14.273	796.7378	TG 46:0	TG 16:0_16:0_14:0	[M+NH4]+	C49H94O6	778.7050	3
14.282	796.7401	TG 46:0	TG 16:0_14:0_16:0	[M+NH4]+	C49H94O6	778.7050	3
14.284	796.7384	TG 46:0	TG 16:0_15:0_15:0	[M+NH4]+	C49H94O6	778.7050	3
14.285	796.7394	TG 46:0	TG 16:0_17:0_13:0	[M+NH4]+	C49H94O6	778.7050	3
13.752	794.7238	TG 46:1	TG 14:0_14:0_18:1	[M+NH4]+	C49H9O26	776.6894	3
13.752	794.7233	TG 46:1	TG 14:0_16:0_16:1	[M+NH4]+	C49H9O26	776.6894	3
13.732	794.7241	TG 46:1	TG 14:0_18:1_14:0	[M+NH4]+	C49H9O26	776.6894	3
13.740	794.7238	TG 46:1	TG 14:1_16:0_16:0	[M+NH4]+	C49H9O26	776.6894	3
13.732	794.7241	TG 46:1	TG 16:0_12:0_18:1	[M+NH4]+	C49H9O26	776.6894	3
13.748	794.7244	TG 46:1	TG 16:0_18:1_12:0	[M+NH4]+	C49H9O26	776.6894	3
13.748	794.7244	TG 46:1	TG 16:1_16:0_14:0	[M+NH4]+	C49H9O26	776.6894	3
13.362	792.7088	TG 46:2	TG 12:0_18:2_16:0	[M+NH4]+	C49H90O6	774.6737	3
13.398	792.7081	TG 46:2	TG 14:0_14:0_18:2	[M+NH4]+	C49H90O6	774.6737	3
13.346	792.7083	TG 46:2	TG 14:0_14:1_18:1	[M+NH4]+	C49H90O6	774.6737	3
13.367	792.7081	TG 46:2	TG 14:0_16:1_16:1	[M+NH4]+	C49H90O6	774.6737	3
13.398	792.7081	TG 46:2	TG 14:0_18:2_14:0	[M+NH4]+	C49H90O6	774.6737	3
13.372	792.7081	TG 46:2	TG 15:1_15:1_16:0	[M+NH4]+	C49H90O6	774.6737	3
13.367	792.7095	TG 46:2	TG 16:0_12:0_18:2	[M+NH4]+	C49H90O6	774.6737	3
13.362	792.7088	TG 46:2	TG 18:1_16:1_12:0	[M+NH4]+	C49H90O6	774.6737	3
14.868	824.7726	TG 48:0	TG 15:0_15:0_18:0	[M+NH4]+	C51H98O6	806.7363	3
14.859	824.7707	TG 48:0	TG 15:0_16:0_17:0	[M+NH4]+	C51H98O6	806.7363	3
14.849	824.7678	TG 48:0	TG 16:0_14:0_18:0	[M+NH4]+	C51H98O6	806.7363	3
14.857	824.7701	TG 48:0	TG 16:0/16:0/16:0	[M+NH4]+	C51H98O6	806.7363	2
14.856	824.7693	TG 48:0	TG 17:0_14:0_17:0	[M+NH4]+	C51H98O6	806.7363	3
14.856	824.7707	TG 48:0	TG 18:0_12:0_18:0	[M+NH4]+	C51H98O6	806.7363	3
14.252	822.7553	TG 48:1	TG 14:0_16:0_18:1	[M+NH4]+	C51H96O6	804.7207	3
14.184	822.7560	TG 48:1	TG 16:0_14:0_18:1	[M+NH4]+	C51H96O6	804.7207	3
14.259	822.7549	TG 48:1	TG 16:0_16:0_16:1	[M+NH4]+	C51H96O6	804.7207	3

14.184	822.7560	TG 48:1	TG 16:0_16:1_16:0	[M+NH4]+	C51H96O6	804.7207	3
14.290	822.7560	TG 48:1	TG 16:0_18:1_14:0	[M+NH4]+	C51H96O6	804.7207	3
14.184	822.7560	TG 48:1	TG 16:1_18:0_14:0	[M+NH4]+	C51H96O6	804.7207	3
13.776	820.7393	TG 48:2	TG 14:0_16:0_18:2	[M+NH4]+	C51H94O6	802.7050	3
13.775	820.7390	TG 48:2	TG 14:0_18:1_16:1	[M+NH4]+	C51H94O6	802.7050	3
13.821	820.7400	TG 48:2	TG 14:0_18:2_16:0	[M+NH4]+	C51H94O6	802.7050	3
13.777	825.6946	TG 48:2	TG 16:0_16:0_16:2	[M+Na]+	C51H94O6	802.7050	3
13.775	820.7394	TG 48:2	TG 16:0_16:1_16:1	[M+NH4]+	C51H94O6	802.7050	3
13.710	820.7400	TG 48:2	TG 16:0_18:1_14:1	[M+NH4]+	C51H94O6	802.7050	3
13.710	820.7400	TG 48:2	TG 16:0_18:2_14:0	[M+NH4]+	C51H94O6	802.7050	3
13.710	820.7400	TG 48:2	TG 16:1_16:0_16:1	[M+NH4]+	C51H94O6	802.7050	3
13.710	820.7400	TG 48:2	TG 16:1_18:1_14:0	[M+NH4]+	C51H94O6	802.7050	3
13.387	818.7249	TG 48:3	TG 14:0_16:0_18:3	[M+NH4]+	C51H9O26	800.6894	3
13.370	818.7234	TG 48:3	TG 14:0_16:1_18:2	[M+NH4]+	C51H9O26	800.6894	3
13.375	818.7242	TG 48:3	TG 14:0_16:2_18:1	[M+NH4]+	C51H9O26	800.6894	3
13.401	818.7259	TG 48:3	TG 14:0_18:2_16:1	[M+NH4]+	C51H9O26	800.6894	3
13.401	818.7259	TG 48:3	TG 14:0_18:3_16:0	[M+NH4]+	C51H9O26	800.6894	3
13.384	818.7231	TG 48:3	TG 16:1/16:1/16:1	[M+NH4]+	C51H9O26	800.6894	2
13.375	818.7242	TG 48:3	TG 16:1_16:2_16:0	[M+NH4]+	C51H9O26	800.6894	3
13.375	818.7242	TG 48:3	TG 16:1_18:1_14:1	[M+NH4]+	C51H9O26	800.6894	3
13.375	818.7242	TG 48:3	TG 16:1_18:2_14:0	[M+NH4]+	C51H9O26	800.6894	3
13.355	818.7213	TG 48:3	TG 18:2_16:1_14:0	[M+NH4]+	C51H9O26	800.6894	3
13.371	818.7228	TG 48:3	TG 18:2_14:0_16:1	[M+NH4]+	C51H9O26	800.6894	3
13.375	818.7242	TG 48:3	TG 18:2_14:1_16:0	[M+NH4]+	C51H9O26	800.6894	3
14.529	836.7690	TG 49:1	TG 16:0_15:0_18:1	[M+NH4]+	C52H98O6	818.7363	3
14.544	836.7690	TG 49:1	TG 16:0_16:0_17:1	[M+NH4]+	C52H98O6	818.7363	3
14.549	836.7714	TG 49:1	TG 16:0_17:0_16:1	[M+NH4]+	C52H98O6	818.7363	3
14.529	836.7690	TG 49:1	TG 16:0_17:1_16:0	[M+NH4]+	C52H98O6	818.7363	3
14.017	834.7548	TG 49:2	TG 16:0_16:1_17:1	[M+NH4]+	C52H96O6	816.7207	3
15.575	852.8022	TG 50:0	TG 16:0_16:0_18:0	[M+NH4]+	C53H10O26	834.7676	3
15.581	852.8020	TG 50:0	TG 16:0_17:0_17:0	[M+NH4]+	C53H10O26	834.7676	3
15.581	852.8032	TG 50:0	TG 16:0_18:0_16:0	[M+NH4]+	C53H10O26	834.7676	3
14.846	850.7863	TG 50:1	TG 16:0_18:1_16:0	[M+NH4]+	C53H100O6	832.7520	3
14.829	850.7866	TG 50:1	TG 16:0_16:0_18:1	[M+NH4]+	C53H100O6	832.7520	3
14.895	850.7883	TG 50:1	TG 16:0_16:1_18:0	[M+NH4]+	C53H100O6	832.7520	3
14.888	850.7877	TG 50:1	TG 16:0_18:0_16:1	[M+NH4]+	C53H100O6	832.7520	3
14.895	850.7883	TG 50:1	TG 18:1_14:0_18:0	[M+NH4]+	C53H100O6	832.7520	3
14.790	850.7883	TG 50:1	TG 18:1_18:0_14:0	[M+NH4]+	C53H100O6	832.7520	3
14.281	848.7715	TG 50:2	TG 16:0_16:0_18:2	[M+NH4]+	C53H98O6	830.7363	3
14.281	848.7727	TG 50:2	TG 16:0_16:1_18:1	[M+NH4]+	C53H98O6	830.7363	3
14.225	848.7726	TG 50:2	TG 16:0_18:1_16:1	[M+NH4]+	C53H98O6	830.7363	3

14.271	848.7723	TG 50:2	TG 16:0_18:2_16:0	[M+NH4]+	C53H98O6	830.7363	3
14.257	848.7712	TG 50:2	TG 18:1_18:1_14:0	[M+NH4]+	C53H98O6	830.7363	3
14.271	848.7723	TG 50:2	TG 18:1_14:0_18:1	[M+NH4]+	C53H98O6	830.7363	3
14.255	848.7726	TG 50:2	TG 18:1_16:1_16:0	[M+NH4]+	C53H98O6	830.7363	3
14.225	848.7726	TG 50:2	TG 18:2_16:0_16:0	[M+NH4]+	C53H98O6	830.7363	3
14.255	848.7719	TG 50:2	TG 18:2_18:0_14:0	[M+NH4]+	C53H98O6	830.7363	3
13.962	846.7551	TG 50:3	TG 16:0_16:0_18:3	[M+NH4]+	C53H96O6	828.7207	3
13.791	846.7551	TG 50:3	TG 16:0_16:1_18:2	[M+NH4]+	C53H96O6	828.7207	3
13.843	846.7559	TG 50:3	TG 16:0_18:3_16:0	[M+NH4]+	C53H96O6	828.7207	3
13.737	846.7559	TG 50:3	TG 16:1_16:0_18:2	[M+NH4]+	C53H96O6	828.7207	3
13.798	846.7550	TG 50:3	TG 16:1_16:1_18:1	[M+NH4]+	C53H96O6	828.7207	3
13.751	846.7562	TG 50:3	TG 16:1_18:1_16:1	[M+NH4]+	C53H96O6	828.7207	3
13.843	846.7559	TG 50:3	TG 18:1_14:0_18:2	[M+NH4]+	C53H96O6	828.7207	3
13.849	846.7569	TG 50:3	TG 18:1_16:2_16:0	[M+NH4]+	C53H96O6	828.7207	3
13.751	846.7562	TG 50:3	TG 18:2_14:0_18:1	[M+NH4]+	C53H96O6	828.7207	3
13.751	846.7562	TG 50:3	TG 18:2_16:0_16:1	[M+NH4]+	C53H96O6	828.7207	3
13.849	846.7569	TG 50:3	TG 18:2_18:1_14:0	[M+NH4]+	C53H96O6	828.7207	3
13.420	844.7374	TG 50:4	TG 14:0_18:1_18:3	[M+NH4]+	C53H94O6	826.7050	3
13.450	844.7361	TG 50:4	TG 14:0_18:2_18:2	[M+NH4]+	C53H94O6	826.7050	3
13.439	844.7369	TG 50:4	TG 16:1_16:0_18:3	[M+NH4]+	C53H94O6	826.7050	3
13.378	844.7392	TG 50:4	TG 16:1_16:2_18:1	[M+NH4]+	C53H94O6	826.7050	3
13.441	844.7406	TG 50:4	TG 18:1_16:2_16:1	[M+NH4]+	C53H94O6	826.7050	3
13.378	844.7392	TG 50:4	TG 18:2_14:0_18:2	[M+NH4]+	C53H94O6	826.7050	3
13.378	844.7392	TG 50:4	TG 18:2_14:1_18:1	[M+NH4]+	C53H94O6	826.7050	3
13.439	844.7369	TG 50:4	TG 18:2_16:0_16:2	[M+NH4]+	C53H94O6	826.7050	3
13.378	844.7392	TG 50:4	TG 18:2_16:1_16:1	[M+NH4]+	C53H94O6	826.7050	3
13.441	844.7406	TG 50:4	TG 18:3_16:0_16:1	[M+NH4]+	C53H94O6	826.7050	3
14.564	862.7885	TG 51:2	TG 16:0_18:2_17:0	[M+NH4]+	C54H100O6	844.7520	3
14.564	862.7885	TG 51:2	TG 17:1_18:1_16:0	[M+NH4]+	C54H100O6	844.7520	3
14.520	862.7887	TG 51:2	TG 18:1_16:1_17:0	[M+NH4]+	C54H100O6	844.7520	3
14.520	862.7887	TG 51:2	TG 18:1_17:1_16:0	[M+NH4]+	C54H100O6	844.7520	3
14.061	860.7711	TG 51:3	TG 16:1_18:1_17:1	[M+NH4]+	C54H98O6	842.7363	3
14.043	860.7715	TG 51:3	TG 17:1_16:1_18:1	[M+NH4]+	C54H98O6	842.7363	3
14.045	860.7682	TG 51:3	TG 17:1/17:1/17:1	[M+NH4]+	C54H98O6	842.7363	2
14.043	860.7715	TG 51:3	TG 18:1_17:0_16:2	[M+NH4]+	C54H98O6	842.7363	3
14.061	860.7711	TG 51:3	TG 18:2_15:0_18:1	[M+NH4]+	C54H98O6	842.7363	3
14.043	860.7715	TG 51:3	TG 18:2_16:0_17:1	[M+NH4]+	C54H98O6	842.7363	3
14.043	860.7715	TG 51:3	TG 18:2_16:1_17:0	[M+NH4]+	C54H98O6	842.7363	3
14.061	860.7711	TG 51:3	TG 18:2_18:1_15:0	[M+NH4]+	C54H98O6	842.7363	3
15.594	878.8177	TG 52:1	TG 16:0_16:0_20:1	[M+NH4]+	C55H104O6	860.7833	3
15.539	878.8181	TG 52:1	TG 16:0_18:0_18:1	[M+NH4]+	C55H104O6	860.7833	3

15.520	878.8192	TG 52:1	TG 16:0_18:1_18:0	[M+NH4]+	C55H104O6	860.7833	3
15.656	878.8196	TG 52:1	TG 18:0_18:1_16:0	[M+NH4]+	C55H104O6	860.7833	3
14.883	876.8020	TG 52:2	TG 14:0_18:1_20:1	[M+NH4]+	C55H10O26	858.7676	3
14.845	876.8020	TG 52:2	TG 16:0_18:0_18:2	[M+NH4]+	C55H10O26	858.7676	3
14.826	876.8025	TG 52:2	TG 16:0_18:1_18:1	[M+NH4]+	C55H10O26	858.7676	3
14.867	876.8043	TG 52:2	TG 16:0_18:2_18:0	[M+NH4]+	C55H10O26	858.7676	3
14.836	876.8020	TG 52:2	TG 18:1_18:1_16:0	[M+NH4]+	C55H10O26	858.7676	3
14.760	876.8043	TG 52:2	TG 18:1_16:0_18:1	[M+NH4]+	C55H10O26	858.7676	3
14.979	876.8043	TG 52:2	TG 18:1_16:1_18:0	[M+NH4]+	C55H10O26	858.7676	3
14.318	874.7864	TG 52:3	TG 16:0_16:0_20:3	[M+NH4]+	C55H100O6	856.7520	3
14.279	874.7867	TG 52:3	TG 16:0_18:1_18:2	[M+NH4]+	C55H100O6	856.7520	3
14.431	874.7876	TG 52:3	TG 16:0_18:2_18:1	[M+NH4]+	C55H100O6	856.7520	3
14.431	874.7876	TG 52:3	TG 16:0_18:3_18:0	[M+NH4]+	C55H100O6	856.7520	3
14.237	874.7878	TG 52:3	TG 16:0_20:2_16:1	[M+NH4]+	C55H100O6	856.7520	3
14.318	874.7864	TG 52:3	TG 16:0_20:3_16:0	[M+NH4]+	C55H100O6	856.7520	3
14.431	874.7876	TG 52:3	TG 18:0_16:1_18:2	[M+NH4]+	C55H100O6	856.7520	3
14.293	874.7865	TG 52:3	TG 18:1_18:1_16:1	[M+NH4]+	C55H100O6	856.7520	3
14.214	874.7881	TG 52:3	TG 18:1_16:0_18:2	[M+NH4]+	C55H100O6	856.7520	3
14.208	874.7876	TG 52:3	TG 18:1_16:1_18:1	[M+NH4]+	C55H100O6	856.7520	3
14.208	874.7876	TG 52:3	TG 18:1_18:2_16:0	[M+NH4]+	C55H100O6	856.7520	3
14.311	874.7876	TG 52:3	TG 18:2_16:0_18:1	[M+NH4]+	C55H100O6	856.7520	3
14.103	872.7702	TG 52:4	TG 16:0_16:0_20:4	[M+NH4]+	C55H98O6	854.7363	3
13.873	872.7728	TG 52:4	TG 16:0_16:1_20:3	[M+NH4]+	C55H98O6	854.7363	3
13.819	872.7715	TG 52:4	TG 16:0_18:1_18:3	[M+NH4]+	C55H98O6	854.7363	3
13.830	872.7710	TG 52:4	TG 16:0_18:2_18:2	[M+NH4]+	C55H98O6	854.7363	3
13.873	872.7728	TG 52:4	TG 16:0_18:3_18:1	[M+NH4]+	C55H98O6	854.7363	3
13.786	872.7718	TG 52:4	TG 16:0_20:3_16:1	[M+NH4]+	C55H98O6	854.7363	3
13.836	872.7697	TG 52:4	TG 16:1_18:1_18:2	[M+NH4]+	C55H98O6	854.7363	3
13.786	872.7718	TG 52:4	TG 18:1_16:2_18:1	[M+NH4]+	C55H98O6	854.7363	3
13.826	872.7697	TG 52:4	TG 18:2_18:2_16:0	[M+NH4]+	C55H98O6	854.7363	3
13.770	872.7726	TG 52:4	TG 18:2_16:0_18:2	[M+NH4]+	C55H98O6	854.7363	3
13.786	872.7718	TG 52:4	TG 18:2_16:1_18:1	[M+NH4]+	C55H98O6	854.7363	3
13.770	872.7726	TG 52:4	TG 18:2_18:1_16:1	[M+NH4]+	C55H98O6	854.7363	3
13.557	870.7557	TG 52:5	TG 16:0_16:1_20:4	[M+NH4]+	C55H96O6	852.7207	3
13.482	870.7549	TG 52:5	TG 16:0_18:2_18:3	[M+NH4]+	C55H96O6	852.7207	3
13.530	870.7566	TG 52:5	TG 16:0_18:4_18:1	[M+NH4]+	C55H96O6	852.7207	3
13.503	870.7553	TG 52:5	TG 16:1_16:1_20:3	[M+NH4]+	C55H96O6	852.7207	3
13.516	875.7107	TG 52:5	TG 16:1_18:1_18:3	[M+NH4]+	C55H96O6	852.7207	3
13.497	870.7541	TG 52:5	TG 16:1_18:2_18:2	[M+NH4]+	C55H96O6	852.7207	3
13.530	870.7566	TG 52:5	TG 18:2_16:0_18:3	[M+NH4]+	C55H96O6	852.7207	3
13.530	870.7566	TG 52:5	TG 18:2_16:1_18:2	[M+NH4]+	C55H96O6	852.7207	3

13.530	870.7566	TG 52:5	TG 18:2_18:1_16:2	[M+NH4]+	C55H96O6	852.7207	3
13.521	870.7560	TG 52:5	TG 18:3_16:0_18:2	[M+NH4]+	C55H96O6	852.7207	3
13.530	870.7566	TG 52:5	TG 18:3_18:1_16:1	[M+NH4]+	C55H96O6	852.7207	3
13.575	870.7578	TG 52:5	TG 20:4_16:0_16:1	[M+NH4]+	C55H96O6	852.7207	3
13.235	868.7398	TG 52:6	TG 18:2_16:0_18:4	[M+NH4]+	C55H94O6	850.7050	3
13.212	868.7366	TG 52:6	TG 16:1_18:3_18:2	[M+NH4]+	C55H94O6	850.7050	3
13.237	868.7390	TG 52:6	TG 16:1_18:4_18:1	[M+NH4]+	C55H94O6	850.7050	3
13.237	868.7390	TG 52:6	TG 18:2_18:1_16:3	[M+NH4]+	C55H94O6	850.7050	3
13.237	868.7390	TG 52:6	TG 18:2_18:3_16:1	[M+NH4]+	C55H94O6	850.7050	3
13.185	868.7412	TG 52:6	TG 18:2_18:4_16:0	[M+NH4]+	C55H94O6	850.7050	3
13.237	868.7390	TG 52:6	TG 20:4_16:1_16:1	[M+NH4]+	C55H94O6	850.7050	3
14.555	888.8026	TG 53:3	TG 17:1_18:0_18:2	[M+NH4]+	C56H10O26	870.7676	3
14.555	888.8026	TG 53:3	TG 17:1_18:1_18:1	[M+NH4]+	C56H10O26	870.7676	3
14.562	888.8030	TG 53:3	TG 18:1_16:1_19:1	[M+NH4]+	C56H10O26	870.7676	3
14.562	888.8030	TG 53:3	TG 18:1_17:0_18:2	[M+NH4]+	C56H10O26	870.7676	3
14.562	888.8030	TG 53:3	TG 18:1_17:1_18:1	[M+NH4]+	C56H10O26	870.7676	3
14.555	888.8026	TG 53:3	TG 18:2_17:0_18:1	[M+NH4]+	C56H10O26	870.7676	3
14.562	888.8030	TG 53:3	TG 18:2_17:1_18:0	[M+NH4]+	C56H10O26	870.7676	3
14.562	888.8030	TG 53:3	TG 18:2_19:1_16:0	[M+NH4]+	C56H10O26	870.7676	3
16.394	906.8499	TG 54:1	TG 18:0_18:0_18:1	[M+NH4]+	C57H108O6	888.8146	3
16.446	906.8507	TG 54:1	TG 18:0_18:1_18:0	[M+NH4]+	C57H108O6	888.8146	3
16.397	906.8501	TG 54:1	TG 18:1_16:0_20:0	[M+NH4]+	C57H108O6	888.8146	3
16.396	906.8495	TG 54:1	TG 18:1_18:0_18:0	[M+NH4]+	C57H108O6	888.8146	3
16.396	906.8495	TG 54:1	TG 18:1_20:0_16:0	[M+NH4]+	C57H108O6	888.8146	3
15.586	904.8333	TG 54:2	TG 18:0_18:0_18:2	[M+NH4]+	C57H106O6	886.7989	3
15.524	904.8335	TG 54:2	TG 18:0_18:1_18:1	[M+NH4]+	C57H106O6	886.7989	3
15.551	904.8346	TG 54:2	TG 18:0_18:2_18:0	[M+NH4]+	C57H106O6	886.7989	3
15.586	904.8344	TG 54:2	TG 18:1_16:0_20:1	[M+NH4]+	C57H106O6	886.7989	3
15.542	904.8344	TG 54:2	TG 18:1_18:0_18:1	[M+NH4]+	C57H106O6	886.7989	3
15.542	904.8344	TG 54:2	TG 18:1_20:1_16:0	[M+NH4]+	C57H106O6	886.7989	3
14.950	902.8188	TG 54:3	TG 18:1_16:0_20:2	[M+NH4]+	C57H104O6	884.7833	3
14.750	902.8193	TG 54:3	TG 18:1_18:0_18:2	[M+NH4]+	C57H104O6	884.7833	3
14.831	902.8183	TG 54:3	TG 18:1/18:1/18:1	[M+NH4]+	C57H104O6	884.7833	2
14.732	902.8188	TG 54:3	TG 18:1_18:2_18:0	[M+NH4]+	C57H104O6	884.7833	3
14.950	902.8188	TG 54:3	TG 18:2_16:0_20:1	[M+NH4]+	C57H104O6	884.7833	3
14.357	900.8037	TG 54:4	TG 18:1_20:3_16:0	[M+NH4]+	C57H10O26	882.7676	3
14.375	900.8032	TG 54:4	TG 18:1_16:0_20:3	[M+NH4]+	C57H10O26	882.7676	3
14.348	900.8043	TG 54:4	TG 18:1_18:0_18:3	[M+NH4]+	C57H10O26	882.7676	3
14.291	900.8025	TG 54:4	TG 18:1_18:1_18:2	[M+NH4]+	C57H10O26	882.7676	3
14.246	900.8043	TG 54:4	TG 18:1_18:2_18:1	[M+NH4]+	C57H10O26	882.7676	3
14.306	900.8021	TG 54:4	TG 18:2_18:2_18:0	[M+NH4]+	C57H10O26	882.7676	3

14.348	900.8043	TG 54:4	TG 18:2_18:0_18:2	[M+NH4]+	C57H10O26	882.7676	3
14.246	900.8043	TG 54:4	TG 18:2_20:2_16:0	[M+NH4]+	C57H10O26	882.7676	3
14.043	898.7872	TG 54:5	TG 14:0_22:5_18:0	[M+NH4]+	C57H100O6	880.7520	3
14.053	898.7864	TG 54:5	TG 16:0_16:0_22:5	[M+NH4]+	C57H100O6	880.7520	3
13.875	898.7862	TG 54:5	TG 18:1_18:1_18:3	[M+H4N]+	C57H100O6	880.7520	3
13.848	898.7866	TG 54:5	TG 18:1_18:2_18:2	[M+NH4]+	C57H100O6	880.7520	3
13.918	898.7880	TG 54:5	TG 18:1_18:3_18:1	[M+NH4]+	C61H100O6	880.7520	3
13.865	898.7860	TG 54:5	TG 18:2_18:2_18:1	[M+NH4]+	C57H100O6	880.7520	3
13.918	898.7880	TG 54:5	TG 18:2_18:0_18:3	[M+NH4]+	C57H100O6	880.7520	3
13.820	898.7880	TG 54:5	TG 18:2_18:1_18:2	[M+NH4]+	C57H100O6	880.7520	3
13.815	898.7875	TG 54:5	TG 18:2_18:3_18:0	[M+NH4]+	C57H100O6	880.7520	3
14.076	898.7863	TG 54:5	TG 20:4_16:0_18:1	[M+NH4]+	C57H100O6	880.7520	3
13.856	896.7699	TG 54:6	TG 16:0_16:0_22:6	[M+NH4]+	C57H98O6	878.7363	3
13.687	896.7727	TG 54:6	TG 16:0_18:1_20:5	[M+NH4]+	C57H98O6	878.7363	3
13.571	896.7720	TG 54:6	TG 18:1_14:0_22:5	[M+NH4]+	C57H98O6	878.7363	3
13.566	896.7707	TG 54:6	TG 18:1_18:1_18:4	[M+NH4]+	C57H98O6	878.7363	3
13.488	896.7705	TG 54:6	TG 18:1_18:2_18:3	[M+NH4]+	C57H98O6	878.7363	3
13.463	896.7720	TG 54:6	TG 18:1_18:4_18:1	[M+NH4]+	C57H98O6	878.7363	3
13.506	896.7703	TG 54:6	TG 18:2/18:2/18:2	[M+NH4]+	C57H98O6	878.7363	2
13.460	896.7727	TG 54:6	TG 18:2_18:3_18:1	[M+NH4]+	C57H98O6	878.7363	3
13.663	896.7708	TG 54:6	TG 18:2_20:4_16:0	[M+NH4]+	C57H98O6	878.7363	3
13.556	901.7263	TG 54:6	TG 18:3_16:0_20:3	[M+Na]+	C57H98O6	878.7363	3
13.571	896.7720	TG 54:6	TG 18:3_18:1_18:2	[M+NH4]+	C57H98O6	878.7363	3
13.556	896.7727	TG 54:6	TG 18:3_22:3_14:0	[M+NH4]+	C57H98O6	878.7363	3
13.687	896.7720	TG 54:6	TG 20:4_16:0_18:2	[M+NH4]+	C57H98O6	878.7363	3
13.687	896.7727	TG 54:6	TG 20:4_18:2_16:0	[M+NH4]+	C57H98O6	878.7363	3
13.420	894.7551	TG 54:7	TG 16:1_16:1_22:5	[M+NH4]+	C57H96O6	876.7207	3
13.420	894.7545	TG 54:7	TG 16:1_22:5_16:1	[M+NH4]+	C57H96O6	876.7207	3
13.200	894.7525	TG 54:7	TG 18:2_18:3_18:2	[M+NH4]+	C57H96O6	876.7207	3
13.214	894.7567	TG 54:7	TG 18:2_18:1_18:4	[M+NH4]+	C57H96O6	876.7207	3
13.194	894.7548	TG 54:7	TG 18:2_18:2_18:3	[M+NH4]+	C57H96O6	876.7207	3
13.200	894.7525	TG 54:7	TG 18:3_18:3_18:1	[M+NH4]+	C57H96O6	876.7207	3
13.191	894.7567	TG 54:7	TG 18:3_18:1_18:3	[M+NH4]+	C57H96O7	876.7207	3
13.191	894.7567	TG 54:7	TG 18:3_18:2_18:2	[M+NH4]+	C57H96O6	876.7207	3
13.394	894.7567	TG 54:7	TG 20:4_16:1_18:2	[M+NH4]+	C57H96O6	876.7207	3
13.387	894.7560	TG 54:7	TG 20:5_18:2_16:0	[M+NH4]+	C57H96O6	876.7207	3
13.420	894.7520	TG 54:7	TG 22:6_16:1_16:0	[M+NH4]+	C57H96O6	876.7207	3
16.392	932.8646	TG 56:2	TG 18:1_18:1_20:0	[M+NH4]+	C59H110O6	914.8302	3
16.392	932.8663	TG 56:2	TG 18:1_20:0_18:1	[M+NH4]+	C59H110O6	914.8302	3
15.475	930.8472	TG 56:3	TG 18:1_18:1_20:1	[M+NH4]+	C59H108O6	912.8146	3
15.484	930.8495	TG 56:3	TG 18:1_18:2_20:0	[M+NH4]+	C59H108O6	912.8146	3

15.541	930.8518	TG 56:3	TG 18:1_20:1_18:1	[M+NH4]+	C59H108O6	912.8146	3
15.484	930.8495	TG 56:3	TG 18:1_22:2_16:0	[M+NH4]+	C59H108O6	912.8146	3
14.888	928.8350	TG 56:4	TG 16:0_20:1_20:3	[M+NH4]+	C59H106O6	910.7989	3
14.883	928.8353	TG 56:4	TG 16:0_20:2_20:2	[M+NH4]+	C59H106O6	910.7989	3
14.888	928.8350	TG 56:4	TG 16:0_22:2_18:2	[M+NH4]+	C59H106O6	910.7989	3
14.888	928.8333	TG 56:4	TG 18:1_18:1_20:2	[M+NH4]+	C59H106O6	910.7989	3
14.856	928.8334	TG 56:4	TG 18:1_20:1_18:2	[M+H4N]+	C59H106O6	910.7989	3
14.883	928.8353	TG 56:4	TG 18:1_20:2_18:1	[M+NH4]+	C59H106O6	910.7989	3
14.888	928.8350	TG 56:4	TG 18:1_20:3_18:0	[M+NH4]+	C59H106O6	910.7989	3
14.888	928.8350	TG 56:4	TG 18:2_20:0_18:2	[M+NH4]+	C59H106O6	910.7989	3
14.888	928.8350	TG 56:4	TG 20:2_16:0_20:2	[M+NH4]+	C59H106O6	910.7989	3
14.888	928.8350	TG 56:4	TG 20:2_18:2_18:0	[M+NH4]+	C59H106O6	910.7989	3
14.883	928.8353	TG 56:4	TG 20:3_20:1_16:0	[M+NH4]+	C59H106O6	910.7989	3
14.386	926.8178	TG 56:5	TG 18:0_20:2_18:3	[M+NH4]+	C59H104O6	908.7833	3
14.627	926.8177	TG 56:5	TG 18:1_18:1_20:3	[M+NH4]+	C59H104O6	908.7833	3
14.349	926.8196	TG 56:5	TG 18:1_20:3_18:1	[M+NH4]+	C59H104O6	908.7833	3
14.349	926.8196	TG 56:5	TG 18:2_20:1_18:2	[M+NH4]+	C59H104O6	908.7833	3
14.619	926.8198	TG 56:5	TG 20:4_18:0_18:1	[M+NH4]+	C59H104O6	908.7833	3
14.645	926.8196	TG 56:5	TG 20:4_18:1_18:0	[M+NH4]+	C59H104O6	908.7833	3
14.645	926.8196	TG 56:5	TG 22:5_18:0_16:0	[M+NH4]+	C59H104O6	908.7833	3
14.046	924.8009	TG 56:6	TG 16:0_18:1_22:5	[M+NH4]+	C59H10O26	906.7676	3
14.053	924.8035	TG 56:6	TG 16:0_18:2_22:4	[M+NH4]+	C59H10O26	906.7676	3
13.910	924.7999	TG 56:6	TG 16:0_20:3_20:3	[M+NH4]+	C59H10O26	906.7676	3
14.053	924.8035	TG 56:6	TG 16:0_22:5_18:1	[M+NH4]+	C59H10O26	906.7676	3
14.053	924.8035	TG 56:6	TG 16:1_18:1_22:4	[M+NH4]+	C59H10O26	906.7676	3
14.005	924.8038	TG 56:6	TG 18:1_16:0_22:5	[M+NH4]+	C59H10O26	906.7676	3
14.044	924.8038	TG 56:6	TG 18:1_18:1_20:4	[M+NH4]+	C59H10O26	906.7676	3
14.106	924.8038	TG 56:6	TG 18:1_20:3_18:2	[M+NH4]+	C59H10O26	906.7676	3
14.046	924.8027	TG 56:6	TG 18:1_20:4_18:1	[M+NH4]+	C59H10O26	906.7676	3
13.927	924.8027	TG 56:6	TG 18:2_18:3_20:1	[M+NH4]+	C59H10O26	906.7676	3
13.927	924.8027	TG 56:6	TG 20:3_16:0_20:3	[M+NH4]+	C59H10O26	906.7676	3
14.106	924.8038	TG 56:6	TG 20:4_18:0_18:2	[M+NH4]+	C59H10O26	906.7676	3
14.106	924.8038	TG 56:6	TG 20:4_18:1_18:1	[M+NH4]+	C59H10O26	906.7676	3
14.005	924.8038	TG 56:6	TG 22:4_18:2_16:0	[M+NH4]+	C59H10O26	906.7676	3
14.046	924.8027	TG 56:6	TG 22:5_16:0_18:1	[M+NH4]+	C59H10O26	906.7676	3
13.842	922.7867	TG 56:7	TG 16:0_18:1_22:6	[M+NH4]+	C59H100O6	904.7520	3
13.645	922.7865	TG 56:7	TG 16:0_18:2_22:5	[M+NH4]+	C59H100O6	904.7520	3
13.651	927.7424	TG 56:7	TG 18:1_18:1_20:5	[M+Na]+	C59H100O6	904.7520	3
13.647	922.7893	TG 56:7	TG 18:1_18:2_20:4	[M+NH4]+	C59H100O6	904.7520	3
13.632	922.7875	TG 56:7	TG 20:4_18:1_18:2	[M+NH4]+	C59H100O6	904.7520	3
13.632	922.7875	TG 56:7	TG 22:5_18:2_16:0	[M+NH4]+	C59H100O6	904.7520	3

13.810	922.7875	TG 56:7	TG 22:6_18.1_16:0	[M+NH4]+	C59H100O6	904.7520	3
13.459	920.7709	TG 56:8	TG 16:0_18:2_22:6	[M+NH4]+	C59H98O6	902.7363	3
13.438	920.7726	TG 56:8	TG 18:2_20:5_18:1	[M+NH4]+	C59H98O6	902.7363	3
13.438	920.7726	TG 56:8	TG 18:2_22:5_16:1	[M+NH4]+	C59H98O6	902.7363	3
13.435	920.7725	TG 56:8	TG 20:4_18:2_18:2	[M+NH4]+	C59H98O6	902.7363	3
13.438	920.7726	TG 56:8	TG 22:6_16:1_18:1	[M+NH4]+	C59H98O6	902.7363	3
13.435	920.7725	TG 56:8	TG 22:6_18:2_16:0	[M+NH4]+	C59H98O6	902.7363	3
17.447	960.8940	TG 58:2	TG 18:1_18:1_22:0	[M+NH4]+	C61H114O6	942.8615	3
17.447	960.8940	TG 58:2	TG 18:1_22:0_18:1	[M+NH4]+	C61H114O6	942.8615	3
14.409	926.8163	TG 58:5	TG 18:2_20:3_20:0	[M+NH4]+	C61H108O6	936.8146	3
14.084	950.8175	TG 58:7	TG 22:5_18:1_18:1	[M+NH4]+	C61H104O6	938.7833	3
13.879	948.8036	TG 58:8	TG 18:0_20:4_20:4	[M+NH4]+	C61H10O26	930.7676	3
13.879	948.8036	TG 58:8	TG 18:1_20:4_20:3	[M+NH4]+	C61H10O26	930.7676	3
13.879	948.8036	TG 58:8	TG 20:3_18:2_20:3	[M+NH4]+	C61H10O26	930.7676	3
13.879	948.8036	TG 58:8	TG 22:6_18:1_18:1	[M+NH4]+	C61H10O26	930.7676	3
13.879	948.8036	TG 58:8	TG 22:6_18:2_18:0	[M+NH4]+	C61H10O26	930.7676	3
13.485	946.7864	TG 58:9	TG 16:0_20:3_22:6	[M+NH4]+	C61H100O6	928.7520	3
13.471	946.7901	TG 58:9	TG 16:0_20:4_22:5	[M+NH4]+	C61H100O6	928.7520	3
13.453	951.7413	TG 58:9	TG 18:1_20:4_20:4	[M+Na]+	C61H100O6	928.7520	3
13.471	946.7901	TG 58:9	TG 18:2_22:5_18:2	[M+NH4]+	C61H100O6	928.7520	3
13.521	946.7896	TG 58:9	TG 22:5_16:0_20:4	[M+NH4]+	C61H100O6	928.7520	3
13.471	946.7901	TG 58:9	TG 22:6_18:2_18:1	[M+NH4]+	C61H100O6	928.7520	3
1.520	468.3085	LPC 14:0	LPC 0:0/14:0	[M+H]+	C22H46NO7P	467.3012	2
1.771	468.3085	LPC 14:0	LPC 14:0/0:0	[M+H]+	C22H46NO7P	467.3012	2
1.961	482.3240	LPC 15:0	LPC 0:0/15:0	[M+H]+	C23H46NO7P	481.3167	2
2.178	482.3240	LPC 15:1	LPC 15:0/0:0	[M+H]+	C23H46NO7P	481.3167	2
2.399	496.3405	LPC 16:0	LPC 0:0/16:0	[M+H]+	C24H50NO7P	495.3325	2
2.600	496.3409	LPC 16:0	LPC 16:0/0:0	[M+H]+	C24H50NO7P	495.3325	2
1.791	494.3243	LPC 16:1	LPC 0:0/16:1	[M+H]+	C24H48NO7P	493.3168	2
1.953	552.3307	LPC 16:1	LPC 16:1/0:0	[M+CH3COO]-	C24H48NO7P	493.3168	2
3.033	510.3560	LPC 17:0	LPC 17:0/0:0	[M+H]+	C25H52NO7P	509.3481	2
3.269	524.3714	LPC 18:0	LPC 0:0/18:0	[M+H]+	C26H54NO7P	523.3638	2
3.452	524.3719	LPC 18:0	LPC 18:0/0:0	[M+H]+	C26H54NO7P	523.3638	2
2.583	522.3558	LPC 18:1	LPC 0:0/18:1	[M+H]+	C26H52NO7P	521.3481	2
2.776	522.3562	LPC 18:1	LPC 18:1/0:0	[M+H]+	C26H52NO7P	521.3481	2
2.037	520.3401	LPC 18:2	LPC 0:0/18:2	[M+H]+	C26H50NO7P	519.3325	2
2.203	520.3393	LPC 18:2	LPC 18:2/0:0	[M+H]+	C26H50NO7P	519.3325	2
2.595	518.3223	LPC 18:3	LPC 18:3/0:0	[M+H]+	C26H48NO7P	517.3168	2
3.866	538.3874	LPC 19:0	LPC 19:0/0:0	[M+H]+	C27H56NO7P	537.3794	2
4.242	552.4033	LPC 20:0	LPC 20:0/0:0	[M+H]+	C28H58NO7P	551.3951	2
3.554	550.3868	LPC 20:1	LPC 20:1/0:0	[M+H]+	C28H56NO7P	549.3794	2

2.800	548.3703	LPC 20:2	LPC 0:0/20:2	[M+H]+	C28H54NO7P	547.3638	2
2.997	548.3703	LPC 20:2	LPC 20:2/0:0	[M+H]+	C28H54NO7P	547.3638	2
2.322	546.3562	LPC 20:3	LPC 0:0/20:3	[M+H]+	C28H52NO7P	545.3481	2
2.502	546.3562	LPC 20:3	LPC 20:3/0:0	[M+H]+	C28H52NO7P	545.3481	2
1.999	544.3400	LPC 20:4	LPC 0:0/20:4	[M+H]+	C28H50NO7P	543.3325	2
2.146	544.3407	LPC 20:4	LPC 20:4/0:0	[M+H]+	C28H50NO7P	543.3325	2
1.723	542.3246	LPC 20:5	LPC 0:0/20:5	[M+H]+	C28H48NO7P	541.3168	2
2.200	542.3246	LPC 20:5	LPC 20:5/0:0	[M+H]+	C28H48NO7P	541.3168	2
2.667	572.3716	LPC 22:4	LPC 0:0/22:4	[M+H]+	C30H54NO7P	571.3638	2
2.817	572.3716	LPC 22:4	LPC 22:4/0:0	[M+H]+	C30H54NO7P	571.3638	2
2.328	570.3559	LPC 22:5	LPC 22:5/0:0	[M+H]+	C30H52NO7P	569.3481	2
2.339	570.3557	LPC 22:5	LPC 0:0/22:5	[M+H]+	C30H52NO7P	569.3481	2
1.943	568.3408	LPC 22:6	LPC 0:0/22:6	[M+H]+	C30H50NO7P	567.3325	2
2.075	568.3402	LPC 22:6	LPC 22:6/0:0	[M+H]+	C30H50NO7P	567.3325	2
3.005	650.4390	LPC 24:0	LPC 0:0/24:0	[M+H]+	C36H66NO7P	649.4317	2
3.121	608.4653	LPC 24:0	LPC 24:0/0:0	[M+H]+	C32H66NO7P	607.4577	2
2.704	454.2934	LPE 16:0	LPE 16:0/0:0	[M+H]+	C21H44NO7P	453.2855	2
3.555	482.3247	LPE 18:0	LPE 18:0/0:0	[M+H]+	C23H48NO7P	481.3168	2
2.861	480.3107	LPE 18:1	LPE 18:1	[M+H]+	C23H46NO7P	479.3012	3
2.099	502.2930	LPE 20:4	LPE 0:0/20:4	[M+H]+	C25H44NO7P	501.2855	2
2.242	502.2924	LPE 20:4	LPE 20:4/0:0	[M+H]+	C25H44NO7P	501.2855	2
2.755	530.3249	LPE 22:4	LPE 0:0/22:4	[M+H]+	C27H48NO7P	529.3168	2
2.900	530.3249	LPE 22:4	LPE 22:4/0:0	[M+H]+	C27H48NO7P	529.3168	2
2.274	528.3098	LPE 22:5	LPE 0:0/22:5	[M+H]+	C27H46NO7P	527.3012	2
2.450	528.3098	LPE 22:5	LPE 22:5/0:0	[M+H]+	C27H46NO7P	527.3012	2
2.033	526.2928	LPE 22:6	LPE 0:0/22:6	[M+H]+	C27H44NO7P	525.2855	2
2.168	524.2786	LPE 22:6	LPE 22:6/0:0	[M-H]-	C27H44NO7P	525.2855	2
3.085	436.2838	LPE O-16:1	LPE O-16:1; P-16:0	[M-H]-	C21H44NO6P	437.2906	3
3.925	466.3309	LPE O-18:1	LPE O-18:1: P-18:0	[M+H]+	C23H48NO6P	465.3219	3
2.146	483.2731	LPG 16:0	LPG 16:0/0:0	[M-H]-	C22H45O9P	484.2801	2
2.837	599.3203	LPI 18:0	LPI 18:0/0:0	[M-H]-	C27H53O12P	600.3275	2
2.892	524.2994	LPS 180	LPS 18:0	[M-H]-	C24H48NO9P	525.3067	3
6.025	678.5071	PC 28:0	PC 28:0	[M+H]+	C36H72NO8P	677.4996	4
6.635	692.5240	PC 29:0	PC 29:0	[M+H]+	C37H74NO8P	691.5152	4
7.343	764.5466	PC 30:0	PC 16:0/14:0	M+CH3COO	C38H76NO8P	705.5309	2
7.359	706.5389	PC 30:0	PC 14:0/16:0	[M+H]+	C38H76NO8P	705.5309	2
6.256	704.5227	PC 30:1	PC 30:1	[M+H]+	C38H74NO8P	703.5152	4
8.263	778.5603	PC 31:0	PC 15:0_16:0	[M+C2H3O2]-	C39H78NO8P	719.5465	3
8.120	778.5603	PC 31:0	PC 16:0_15:0	[M+C2H3O2]-	C39H78NO8P	719.5465	3
9.303	734.5699	PC 32:0	PC 16:0/16:0	[M+H]+	C40H80NO8P	733.5622	2
7.682	732.5543	PC 32:1	PC 16:0/16:1	[M+H]+	C40H78NO8P	731.5465	2

7.644	732.5565	PC 32:1	PC 16:1/16:0	[M+H]+	C40H78NO8P	731.5465	2
6.538	730.5383	PC 32:2	PC 18:2_14:0	[M+H]+	C40H76NO8P	729.5309	3
6.319	728.5226	PC 32:3	PC 32:3	[M+H]+	C40H74NO8P	727.5152	4
10.027	748.5848	PC 33:0	PC 33:0	[M+H]+	C41H82NO8P	747.5778	4
10.605	748.5848	PC 33:0	PC 33:0	[M+H]+	C41H82NO8P	747.5778	4
8.534	746.5694	PC 33:1	PC 33:1	[M+H]+	C41H80NO8P	745.5622	4
10.743	744.5569	PC 33:2	PC 15:0_18:2	[M+H]+	C41H78NO8P	743.5465	3
8.121	762.5068	PC 33:4	PC 33:4	[M+Na]+	C41H74NO8P	739.5177	4
11.513	762.5998	PC 34:0	PC 16:0/18:0	[M+H]+	C42H84NO8P	761.5935	2
11.525	762.6014	PC 34:0	PC 18:0_16:0	[M+H]+	C42H84NO8P	761.5935	3
9.250	760.5858	PC 34:1	PC 16:0/18:1	[M+H]+	C42H82NO8P	759.5778	2
9.616	760.5858	PC 34:1	PC 18:1/16:0	[M+H]+	C42H82NO8P	759.5778	2
8.078	758.5700	PC 34:2	PC 18:2/16:0	[M+H]+	C42H80NO8P	757.5622	2
7.849	758.5700	PC 34:2	PC 16:0/18:2	[M+H]+	C42H80NO8P	757.5622	2
6.760	756.5541	PC 34:3	PC 34:3	[M+H]+	C42H78NO8P	755.5465	4
7.269	756.5536	PC 34:3	PC 34:3	[M+H]+	C42H78NO8P	755.5465	4
6.385	754.5380	PC 34:4	PC 34:4	[M+H]+	C42H76NO8P	753.5309	4
11.813	776.6149	PC 35:0	PC 35:0	[M+H]+	C43H86NO8P	775.6091	4
10.977	774.5988	PC 35:1	PC 35:1	[M+H]+	C43H84NO8P	773.5935	4
9.139	772.5843	PC 35:2	PC 35:2	[M+H]+	C43H82NO8P	771.5778	4
7.768	766.5393	PC 35:5	PC 35:5	[M+H]+	C43H76NO8P	765.5321	4
7.794	764.5233	PC 35:6	PC 13:0_22:6	[M+H]+	C43H74NO8P	763.5152	3
12.003	790.6345	PC 36:0	PC 20:0_16:0	[M+H]+	C44H88NO8P	789.6248	3
11.580	790.6345	PC 36:0	PC 36:0	[M+H]+	C44H88NO8P	789.6248	4
11.603	788.6169	PC 36:1	PC 18:0_18:1	[M+H]+	C44H86NO8P	787.6091	3
10.377	786.6013	PC 36:2	PC 18:0_18:2	[M+H]+	C44H84NO8P	785.5935	3
9.952	786.6012	PC 36:2	PC 16:0_20:2	[M+H]+	C44H84NO8P	785.5935	3
9.972	786.6002	PC 36:2	PC 18:1/18:1	[M+H]+	C44H84NO8P	785.5935	2
10.383	786.6012	PC 36:2	PC 18:2/18:0	[M+H]+	C44H84NO8P	785.5935	2
8.316	784.5857	PC 36:3	PC 18:1_18:2	[M+H]+	C44H82NO8P	783.5778	3
8.682	784.5857	PC 36:3	PC 18:2_18:1	[M+H]+	C44H82NO8P	783.5778	3
7.769	784.5857	PC 36:3	PC 36:3	[M+H]+	C44H82NO8P	784.5778	4
7.884	782.5700	PC 36:4	PC 16:0_20:4	[M+H]+	C44H80NO8P	781.5622	3
7.111	782.5694	PC 36:4	PC 18:2/18:2	[M+H]+	C44H80NO8P	781.5622	2
6.914	780.5544	PC 36:5	PC 16:0_20:5	[M+H]+	C44H78NO8P	779.5465	3
6.589	780.5540	PC 36:5	PC 36:5	[M+H]+	C44H78NO8P	779.5465	4
6.173	778.5393	PC 36:6	PC 36:6	[M+H]+	C44H76NO8P	777.5309	4
8.887	796.5832	PC 37:4	PC 37:4	[M+H]+	C45H82NO8P	795.5778	4
7.352	794.5685	PC 37:5	PC 19:2_18:3	[M+H]+	C45H80NO8P	793.5622	3
9.889	814.5376	PC 37:6	PC 37:6	[M+Na]+	C45H78NO8P	791.5478	4
7.995	790.5404	PC 37:7	PC 37:7	[M+H]+	C45H76NO8P	789.5309	4

11.692	790.5379	PC 37:7	PC 37:7	[M+H]+	C45H76NO8P	789.5309	4
12.348	818.6609	PC 38:0	PC 22:0_16:0	[M+H]+	C46H92NO8P	817.6548	3
12.017	816.6488	PC 38:1	PC 38:1	[M+H]+	C46H90NO8P	815.6404	4
11.708	814.6331	PC 38:2	PC 18:0_20:2	[M+H]+	C46H88NO8P	813.6248	3
11.708	814.6331	PC 38:2	PC 20:0_18:2	[M+H]+	C46H88NO8P	813.6248	3
11.708	814.6331	PC 38:2	PC 20:1_18:1	[M+H]+	C46H88NO8P	813.6248	3
11.169	812.6166	PC 38:3	PC 38:3	[M+H]+	C46H86NO8P	811.6091	4
10.069	810.6011	PC 38:4	PC 18:0_20:4	[M+H]+	C46H84NO8P	809.5935	3
8.773	810.5998	PC 38:4	PC 38:4	[M+H]+	C46H84NO8P	809.5935	4
9.371	810.6017	PC 38:4	PC 16:0_22:4	[M+H]+	C46H84NO8P	809.5935	3
8.060	808.5862	PC 38:5	PC 16:0_22:5	[M+H]+	C46H82NO8P	807.5778	3
8.668	808.5850	PC 38:5	PC 18:0_20:5	[M+H]+	C46H82NO8P	807.5778	3
8.060	808.5862	PC 38:5	PC 18:1_20:4	[M+H]+	C46H82NO8P	807.5778	3
8.060	808.5862	PC 38:5	PC 20:4_18:1	[M+H]+	C46H82NO8P	807.5778	3
7.542	806.5703	PC 38:6	PC 16:0_22:6	[M+H]+	C46H80NO8P	805.5622	3
7.373	806.5703	PC 38:6	PC 22:6_16:0	[M+H]+	C46H80NO8P	805.5622	3
6.924	806.5701	PC 38:6	PC 38:6	[M+H]+	C46H80NO8P	805.5622	4
6.385	804.5541	PC 38:7	PC 38:7	[M+H]+	C46H78NO8P	803.5478	4
11.986	832.6786	PC 39:0	PC 39:0	[M+H]+	C47H94NO8P	831.6667	4
11.299	824.6172	PC 39:4	PC 39:4	[M+H]+	C47H86NO8P	823.6091	4
8.463	820.5893	PC 39:6	PC 39:6	[M+H]+	C47H82NO8P	819.5778	4
12.106	868.6747	PC 40:0	PC 40:0	[M+Na]+	C48H96NO8P	845.6874	4
12.668	846.6968	PC 40:0	PC 40:0	[M+H]+	C48H96NO8P	845.6874	4
12.338	844.6798	PC 40:1	PC 40:1	[M+H]+	C48H94NO8P	843.6717	4
12.058	842.6649	PC 40:2	PC 40:2	[M+H]+	C48H92NO8P	841.6561	4
11.795	840.6488	PC 40:3	PC 40:3	[M+H]+	C48H90NO8P	839.6404	4
11.517	838.6321	PC 40:4	PC 40:4	[M+H]+	C48H88NO8P	837.6248	4
10.266	836.6160	PC 40:5	PC 40:5	[M+H]+	C48H86NO8P	835.6091	4
8.314	834.6021	PC 40:6	PC 40:6	[M+H]+	C48H84NO8P	833.5935	4
9.538	834.6013	PC 40:6	PC 40:6	[M+H]+	C48H84NO8P	833.5935	4
7.063	832.5853	PC 40:7	PC 40:7	[M+H]+	C48H82NO8P	831.5778	4
7.742	832.5852	PC 40:7	PC 18:1/22:6	[M+H]+	C48H82NO8P	831.5778	2
9.361	832.5819	PC 40:7	PC 40:7	[M+H]+	C48H82NO8P	831.5778	4
6.674	830.5692	PC 40:8	PC 40:8	[M+H]+	C48H80NO8P	829.5622	4
12.669	872.7121	PC 42:1	PC 42:1	[M+H]+	C50H98NO8P	871.7030	4
12.385	870.6964	PC 42:2	PC 42:2	[M+H]+	C50H96NO8P	869.6874	4
12.106	868.6804	PC 42:3	PC 42:3	[M+H]+	C50H94NO8P	867.6717	4
11.919	866.6639	PC 42:4	PC 42:4	[M+H]+	C50H92NO8P	865.6561	4
11.695	864.6449	PC 42:5	PC 42:5	[M+H]+	C50H90NO8P	863.6404	4
6.447	854.5705	PC 42:10	PC 42:10	[M+H]+	C50H80NO8P	853.5622	4
6.215	878.5692	PC 44:12	PC 22:6/22:6	[M+H]+	C52H80NO8P	877.5622	2

8.536	692.5569	PC O-30:0	PC O-30:0	[M+H]+	C38H78NO7P	691.5497	4
8.293	690.5450	PC O-30:1	PC O-30:1	[M+H]+	C38H76NO7P	689.5378	4
10.961	720.5907	PC O-32:0	PC O-32:0	[M+H]+	C40H82NO7P	719.5835	4
10.635	718.5746	PC O-32:1	PC O-32:1	[M+H]+	C40H80NO7P	717.5674	4
11.901	748.6232	PC O-34:0	PC O-34:0	[M+H]+	C42H86NO7P	747.6160	4
11.180	804.6122	PC O-34:1	PC O-18:1_16:0	[M+CH3COO]-	C42H84NO7P	745.5985	3
9.391	744.5894	PC O-34:2	PC O-34:2	[M+H]+	C42H82NO7P	743.5841	4
11.920	774.6397	PC O-36:1	PC O-36:1	[M+H]+	C44H88NO7P	773.6298	4
11.411	772.6223	PC O-36:2	PC O-36:2	[M+H]+	C44H86NO7P	771.6154	4
9.632	770.6022	PC O-36:3	PC O-36:3	[M+H]+	C44H84NO7P	769.6010	4
9.162	768.5908	PC O-36:4	PC O-36:4	[M+H]+	C44H82NO7P	767.5866	4
8.814	766.5746	PC O-36:5	PC O-16:1_20:4	[M+H]+	C44H80NO7P	765.5672	2
11.215	778.5710	PC O-37:6	PC O-37:6	[M+H]+	C45H80NO7P	777.5672	4
11.027	796.6207	PC O-38:4	PC O-38:4	[M+H]+	C46H86NO7P	795.6142	4
8.710	792.5910	PC O-38:6	PC O-38:6	[M+H]+	C46H82NO7P	791.5854	4
8.331	790.5754	PC O-38:7	PC O-38:7	[M+H]+	C46H80NO7P	789.5710	4
11.805	824.6540	PC O-40:4	PC O-40:4	[M+H]+	C48H90NO7P	823.6455	4
8.911	818.6044	PC O-40:7	PC O-40:7	[M+H]+	C48H84NO7P	817.6023	4
9.385	816.5874	PC O-40:8	PC O-40:8	[M+H]+	C48H82NO7P	815.5879	4
9.647	692.5230	PE 32:0	PE 32:0	[M+H]+	C37H74NO8P	691.5152	4
7.908	690.5077	PE 32:1	PE 16:0_16:1	[M+H]+	C37H72NO8P	689.4996	3
9.996	718.5382	PE 34:1	PE 16:0_18:1	[M+H]+	C39H76NO8P	717.5309	3
8.376	716.5239	PE 34:2	PE 16:0_18:2	[M+H]+	C39H74NO8P	715.5152	3
11.690	746.5700	PE 36:1	PE 18:0_18:1	[M+H]+	C41H80NO8P	745.5622	3
11.688	746.5697	PE 36:1	PE 23:0_13:1	[M+H]+	C41H80NO8P	745.5622	3
10.704	744.5562	PE 36:2	PE 18:0_18:2	[M+H]+	C41H78NO8P	743.5465	3
10.291	744.5544	PE 36:2	PE 18:1/18:1	[M+H]+	C41H78NO8P	743.5465	2
8.634	742.5379	PE 36:3	PE 18:1_18:2	[M+H]+	C41H76NO8P	741.5309	3
8.604	742.5379	PE 36:3	PE 18:2_18:1	[M+H]+	C41H76NO8P	741.5309	3
8.005	738.5080	PE 36:4	PE 16:0_20:4	[M-H]-	C41H74NO8P	739.5152	3
8.200	738.5074	PE 36:4	PE 18:2/18:2	[M-H]-	C41H74NO8P	739.5152	2
10.006	740.5204	PE 36:4	PE 36:4	[M+H]+	C41H74NO8P	739.5152	4
7.114	738.5073	PE 36:5	PE 16:0_20:5	[M+H]+	C41H72NO8P	737.4996	3
11.359	770.5718	PE 38:3	PE 18:0_20:3	[M+H]+	C43H80NO8P	769.5622	3
10.417	768.5543	PE 38:4	PE 18:0_20:4	[M+H]+	C43H78NO8P	767.5465	3
8.236	766.5385	PE 38:5	PE 16:0_22:5	[M+H]+	C43H76NO8P	765.5309	3
8.377	766.5385	PE 38:5	PE 18:1_20:4	[M+H]+	C43H76NO8P	765.5309	3
10.738	766.5371	PE 38:5	PE 38:5	[M+H]+	C43H76NO8P	765.5309	4
7.797	764.5231	PE 38:6	PE 16:0_22:6	[M+H]+	C43H74NO8P	763.5152	3
7.603	764.5231	PE 38:6	PE 22:6_16:0	[M+H]+	C43H74NO8P	763.5152	3
7.127	764.5228	PE 38:6	PE 38:6	[M+H]+	C43H74NO8P	764.5152	4

7.099	760.4899	PE 38:8	PE 38:8	[M+H]+	C43H70NO8P	760.4864	4
11.615	798.5908	PE 40:3	PE 40:3	[M+H]+	C45H84NO8P	797.5922	4
11.618	796.5863	PE 40:4	PE 18:0_22:4	[M+H]+	C45H82NO8P	795.5778	3
10.666	794.5720	PE 40:5	PE 18:0_22:5	[M+H]+	C45H80NO8P	793.5622	3
9.906	792.5550	PE 40:6	PE 18:0_22:6	[M+H]+	C45H78NO8P	791.5465	3
9.835	790.5397	PE 40:6	PE 22:6_18:0	[M-H]-	C45H78NO8P	791.5465	3
10.404	792.5481	PE 40:6	PE 40:6	[M+H]+	C45H78NO8P	792.5465	4
7.900	788.5246	PE 40:7	PE 18:1_22:6	[M-H]-	C45H76NO8P	789.5309	3
8.080	788.5246	PE 40:7	PE 22:6_18:1	[M-H]-	C45H76NO8P	789.5309	3
10.424	790.5378	PE 40:7	PE 40:7	[M+H]+	C45H76NO8P	789.5309	4
6.882	788.5234	PE 40:8	PE 40:8	[M+H]+	C45H74NO8P	787.5152	4
7.794	786.5052	PE 40:9	PE 40:9	[M+H]+	C45H72NO8P	785.4996	4
11.622	818.5690	PE 42:7	PE 42:7	[M+H]+	C47H80NO8P	817.5622	4
9.923	816.5519	PE 42:8	PE 42:8	[M+H]+	C47H78NO8P	815.5478	4
10.713	816.5519	PE 42:8	PE 42:9	[M+H]+	C47H78NO8P	815.5478	4
9.895	814.5369	PE 42:9	PE 19:1_24:0	[M+H]+	C47H76NO8P	813.5309	3
12.000	842.6639	PE 43:1	PE 20:4_20:3;O	[M-H]-	C48H94O8PN	843.6717	3
2.696	454.2932	PE O-16:0	PE O-16:0	[M+H]+	C21H44O7PN	453.2860	3
3.568	482.3248	PE O-18:0	PE O-18:0	[M+H]+	C23H48O7PN	481.3176	3
8.956	674.5096	PE O-32:2	PE O-16:1/16:1	[M+H]+	C37H7O27PN	673.5024	2
11.295	702.5432	PE O-34:2	PE 18:1_O-16:1	[M+H]+	C39H76O7PN	701.5359	3
11.295	702.5432	PE O-34:2	PE O-16:1_18:1	[M+H]+	C39H76O7PN	701.5359	3
9.507	700.5271	PE O-34:3	PE O-16:1_18:2	[M+H]+	C39H74O7PN	699.5203	3
11.898	730.5769	PE O-36:2	PE O-16:1_20:1	[M+H]+	C41H80O7PN	729.5672	3
11.898	730.5769	PE O-36:2	PE O-18:1/18:1	[M+H]+	C41H80O7PN	729.5672	2
11.567	728.5594	PE O-36:3	PE O-18:1_18:2	[M+H]+	C41H78O7PN	727.5516	3
11.443	728.561	PE O-36:3	PE O-18:2_18:1	[M+H]+	C41H78O7PN	727.5538	3
9.551	726.5411	PE O-36:4	PE O-18:2/18:2	[M+H]+	C41H76O7PN	725.5339	2
9.068	722.5139	PE O-36:5	PE O-16:1_20:4	[M-H]-	C41H74O7PN	723.5203	3
7.826	720.498	PE O-36:6	PE O-16:1_20:5	[M-H]-	C41H7O27PN	721.5046	3
11.915	756.5902	PE O-38:3	PE O-20:2_18:1	[M+H]+	C43H8O27PN	755.5828	3
11.425	750.5503	PE O-38:4	PE O-18:0_20:4	[M-H]-	C43H80O7PN	755.5828	3
11.605	752.5594	PE O-38:4	PE O-18:1_20:3	[M-H]-	C43H80O7PN	753.5666	3
11.092	752.5593	PE O-38:5	PE O-16:1_22:4	[M+H]+	C43H78O7PN	751.5516	3
11.392	750.5449	PE O-38:5	PE O-18:1_20:4	[M-H]-	C43H78O7PN	751.5516	3
9.389	750.5436	PE O-38:6	PE O-16:1_22:5	[M+H]+	C43H76O7PN	749.5364	3
9.448	750.5438	PE O-38:6	PE O-18:2_20:4	[M+H]+	C43H76O7PN	749.5359	3
7.985	748.5313	PE O-38:7	PE O-18:2_20:5	[M+H]+	C43H74O7PN	747.5215	3
7.962	748.5303	PE O-38:7	PE O-18:3_20:4	[M+H]+	C43H74O7PN	747.5215	3
11.91	782.6044	PE O-40:4	PE O-18:0_22:4	[M+H]+	C45H84O7PN	781.5985	3
11.911	782.6037	PE O-40:4	PE O-18:1_22:3	[M+H]+	C45H84O7PN	781.5985	3

11.816	778.579	PE O-40:5	PE O-16:1_24:4	[M-H]-	C45H8O27PN	779.5862	3
11.82	778.5789	PE O-40:5	PE O-18:1_22:4	[M-H]-	C45H8O27PN	779.5829	3
11.912	778.5751	PE O-40:5	PE O-20:1_20:4	[M-H]-	C45H8O27PN	779.5823	3
11.507	776.5596	PE O-40:6	PE O-18:0_22:6	[M-H]-	C45H80O7PN	777.5668	3
11.468	776.5601	PE O-40:6	PE O-18:1_22:5	[M-H]-	C45H80O7PN	777.5672	3
9.715	776.5612	PE O-40:7	PE O-18:2_22:5	[M+H]+	C45H78O7PN	775.554	3
8.831	772.5286	PE O-40:8	PE O-18:2_22:6	[M-H]-	C45H76O7PN	773.5359	3
11.11	774.5407	PE O-40:8	PE O-18:4_22:4	[M+H]+	C45H76O7PN	773.5359	3
11.925	806.6022	PE O-42:6	PE O-20:1_22:5	[M+H]+	C47H84O7PN	805.595	3
11.818	802.5751	PE O-42:7	PE O-20:1_22:6	[M-H]-	C47H8O27PN	803.5823	3
5.972	712.5121	PG 30:0	PG 14:0_16:0	[M+H4N]+	C36H71O10P	694.4785	3
7.198	740.5439	PG 32:0	PG 16:0/16:0	[M+H4N]+	C38H75O10P	722.5098	2
6.158	738.5285	PG 32:1	PG 16:0_16:1	[M+H4N]+	C38H73O10P	720.4941	3
7.234	747.5189	PG 34:1	PG 16:0_18:1	[M-H]-	C40H77O10P	748.5254	3
6.445	764.5438	PG 34:2	PG 16:0_18:2	[M+H4N]+	C40H75O10P	746.5098	3
7.491	773.5336	PG 36:2	PG 18:1/18:1	[M-H]-	C42H79O10P	774.5411	2
7.662	773.5335	PG 36:2	PG 18:0_18:2	[M-H]-	C42H79O10P	774.5411	3
6.518	771.5179	PG 36:3	PG 18:1_18:2	[M-H]-	C42H77O10P	772.5254	3
6.317	788.5439	PG 36:4	PG 16:0_20:4	[M+H4N]+	C42H75O10P	770.5098	3
5.691	786.5305	PG 36:5	PG 16:0_20:5	[M+H4N]+	C42H73O10P	768.4941	3
7.640	816.5781	PG 38:4	PG 18:0_20:4	[M+H4N]+	C44H79O10P	798.5411	3
6.439	814.5588	PG 38:5	PG 18:1_20:4	[M+H4N]+	C44H77O10P	796.5254	3
6.445	814.5587	PG 38:5	PG 16:0_22:5	[M+H4N]+	C44H77O10P	796.5254	3
6.449	814.5598	PG 38:5	PG 20:0_18:5	[M+H4N]+	C44H77O10P	796.5254	3
6.340	795.5178	PG 38:5	PG 22:5_16:0	[M-H]-	C44H77O10P	796.5254	3
6.116	812.5438	PG 38:6	PG 16:0_22:6	[M+H4N]+	C44H75O10P	794.5098	3
5.604	819.5178	PG 40:7	PG 18:1_22:6	[M-H]-	C46H77O10P	820.5254	3
5.033	817.5021	PG 40:8	PG 18:2_22:6	[M-H]-	C46H75O10P	818.5098	3
5.122	817.5019	PG 40:8	PG 20:4/20:4	[M-H]-	C46H75O10P	818.5098	2
5.056	817.5024	PG 40:8	PG 22:6_18:2	[M-H]-	C46H75O10P	818.5098	3
5.022	841.5022	PG 42:10	PG 20:4_22:6	[M-H]-	C48H75O10P	842.5098	3
4.846	865.5022	PG 44:12	PG 22:6/22:6	[M-H]-	C50H75O10P	866.5098	2
6.957	828.5517	PI 32:0	PI 32:0	[M+NH4]+	C41H79O13P	810.5258	4
7.039	854.5764	PI 34:1	PI 34:1	[M+NH4]+	C43H81O13P	836.8272	4
6.116	833.5182	PI 34:2	PI 16:0_18:2	[M-H]-	C43H79O13P	834.5258	3
8.574	831.4913	PI 34:3	PI 16:1_18:2	[M-H]-	C43H77O13P	832.5114	3
9.282	850.5275	PI 34:3	PI 34:3	[M+NH4]+	C43H77O13P	832.5114	4
7.402	861.5495	PI 36:2	PI 18:0_18:2	[M-H]-	C45H83O13P	862.5571	3
6.224	878.5673	PI 36:3	PI 36:3	[M+NH4]+	C45H81O13P	860.5427	4
5.997	857.5190	PI 36:4	PI 16:0_20:4	[M-H]-	C45H79O13P	858.5258	3
7.316	906.5959	PI 38:3	PI 38:3	[M+NH4]+	C47H85O13P	888.5715	4

7.156	885.5506	PI 38:4	PI 18:0_20:4	[M-H]-	C47H83O13P	886.5571	3
6.117	883.5337	PI 38:5	PI 16:0_22:5	[M-H]-	C47H81O13P	884.5427	3
6.117	883.5427	PI 38:5	PI 22:5_16:0	[M-H]-	C47H81O13P	882.5283	3
6.126	883.5342	PI 38:5	PI 18:1_20:4	[M-H]-	C47H81O13P	884.5415	3
6.117	883.5427	PI 38:5	PI 20:4_18:1	[M-H]-	C47H81O13P	884.5415	3
5.828	881.5182	PI 38:6	PI 16:0_22:6	[M-H]-	C47H79O13P	882.5258	3
7.410	911.5639	PI 40:5	PI 18:0_22:5	[M-H]-	C49H85O13P	912.5728	3
6.965	909.5496	PI 40:6	PI 18:0_22:6	[M-H]-	C49H83O13P	910.5571	3
8.534	938.5884	PI 42:6	PI 20:0_22:6	[M-H]-	C51H87O13P	939.5956	3
7.576	760.5129	PS 34:1	PS 16:0_18:1	[M-H]-	C40H76NO10P	761.5207	3
7.610	760.5129	PS 34:1	PS 16:1_18:0	[M-H]-	C40H76NO10P	761.5207	3
9.459	790.5607	PS 36:1	PS 18:0_18:1	[M+H]+	C42H80NO10P	789.5520	3
8.383	812.5476	PS 36:1	PS 36:1	[M+Na]+	C42H80NO10P	789.5520	4
7.982	788.5434	PS 36:2	PS 18:0_18:2	[M+H]+	C42H78NO10P	787.5363	3
7.844	786.5285	PS 36:2	PS 18:1/18:1	[M-H]-	C42H78NO10P	787.5363	2
8.076	820.5283	PS 36:4	PS 16:0_20:4	[M-H]-	C45H76NO10P	821.5207	3
9.370	816.5872	PS 38:2	PS 38:2	[M+H]+	C44H82NO10P	815.5664	4
8.606	814.5610	PS 38:3	PS 18:0_20:3	[M+H]+	C44H80NO10P	813.5520	3
8.309	812.5442	PS 38:3	PS 18:2_20:1	[M-H]-	C44H80NO10P	813.5520	3
7.703	812.5429	PS 38:4	PS 18:0_20:4	[M+H]+	C44H78NO10P	811.5363	3
7.803	812.5429	PS 38:4	PS 20:4_18:0	[M+H]+	C44H78NO10P	811.5363	3
6.232	808.5150	PS 38:6	PS 38:6	[M+H]+	C44H74NO10P	807.5050	4
11.631	826.5579	PS 39:3	PS 21:2_18:1	[M-H]-	C45H82NO10P	827.5676	3
11.530	866.5891	PS 40:2	PS 40:2	[M+Na]+	C46H86NO10P	843.5964	4
8.998	838.5598	PS 40:4	PS 18:0_22:4	[M-H]-	C46H82NO10P	839.5676	3
8.126	862.5544	PS 40:4	PS 40:4	[M+Na]+	C46H82NO10P	839.5676	4
7.991	838.5578	PS 40:5	PS 18:0_22:5	[M+H]+	C46H80NO10P	837.5520	3
7.632	836.5422	PS 40:6	PS 18:0_22:6	[M+H]+	C46H78NO10P	835.5363	3
7.426	834.5285	PS 40:6	PS 22:6_18:0	[M-H]-	C46H78NO10P	835.5363	3
8.546	836.5418	PS 40:6	PS 40:6	[M+H]+	C46H78NO10P	835.5363	4
7.591	832.5107	PS 40:7	PS 20:3_20:4	[M-H]-	C46H76NO10P	833.5207	3
7.556	832.5167	PS 40:7	PS 22:6_18:1	[M-H]-	C46H76NO10P	833.5207	3
7.502	832.5178	PS 40:7	PS 22:7_18:0	[M-H]-	C46H76NO10P	833.5207	3
10.217	848.5421	PS 41:6	PS 21:2_20:4	[M-H]-	C47H80NO10P	849.5520	3
9.624	864.5701	PS 42:6	PS 42:6	[M+H]+	C48H82NO10P	863.5639	4
7.357	856.5108	PS 42:9	PS 20:3_22:6	[M-H]-	C48H76NO10P	857.5207	3
9.442	856.5108	PS 42:9	PS 42:9	[M-H]-	C48H76NO10P	857.5207	4
7.166	740.5434	BMP 32:0	BMP 16:0/16:0	[M+NH4]+	C38H75O10P	722.5094	2
6.302	788.5438	BMP 36:4	BMP 16:0_20:4	[M+H4N]+	C42H75O10P	770.5098	3
5.600	838.5594	BMP 40:7	BMP 18:1_22:6	[M+H4N]+	C46H77O10P	820.5254	3
7.949	788.5468	BMP 36:4	BMP 18:2/18:2	[M+NH4]+	C42H75O10P	770.5098	2

5.069	836.5450	BMP 40:8	BMP 18:2_22:6	[M+H4N]+	C46H75O10P	818.5098	3
4.999	860.5439	BMP 42:10	BMP 20:4_22:6	[M+H4N]+	C48H75O10P	842.5098	3
4.871	884.5435	BMP 44:12	BMP 22:6/22:6	[M+H4N]+	C50H75O10P	866.5098	2
11.325	702.4959	CL 68:1	CL 16:0_16:0_18:0_18:1	[M-2H]2-	C77H148O17P2	1407.0192	3
7.668	763.5119	CL 78:10	CL 16:0_18:0_22:4_22:6	[M-2H]2-	C87H150O17P2	1468.0352	3
1.939	213.1867	FA 13:0	FA 13:0	[M-H]-	C13H26O2	214.1939	2
2.347	227.2028	FA 14:0	FA 14:0	[M-H]-	C14H28O2	228.2100	2
2.437	241.2181	FA 15:0	FA 15:0	[M-H]-	C15H30O2	242.2253	2
3.289	511.4737	FA 16:0	FA 16:0	[2M-H]-	C16H3O22	256.2402	2
2.576	253.2177	FA 16:1	FA 16:1	[M-H]-	C16H30O2	254.2246	2
3.719	269.2491	FA 17:0	FA 17:0	[M-H]-	C17H34O2	270.2559	2
3.020	267.2333	FA 17:1	FA 17:1	[M-H]-	C17H3O22	268.2402	2
4.172	567.5360	FA 18:0	FA 18:0	[2M-H]-	C18H36O2	284.2715	2
3.459	281.2497	FA 18:1	FA 18:1	[M-H]-	C18H34O2	282.2559	2
1.376	281.2494	FA 18:1	FA 18:1	[M-H]-	C18H34O3	282.2566	2
4.057	281.2484	FA 18:1	FA 18:1	[M-H]-	C18H34O4	282.2556	2
3.482	281.2493	FA 18:1	FA 18:1	[M-H]-	C18H34O5	282.2565	2
2.860	279.2339	FA 18:2	FA 18:2	[M-H]-	C18H3O22	280.2402	2
2.365	277.2175	FA 18:3	FA 18:3	[M-H]-	C18H30O2	278.2246	2
4.547	297.2800	FA 19:0	FA 19:0	[M-H]-	C19H38O2	298.2872	2
4.965	311.2961	FA 20:0	FA 20:0	[M-H]-	C20H40O2	312.3028	2
4.266	309.2801	FA 20:1	FA 20:1	[M-H]-	C20H38O2	310.2872	2
3.691	307.2645	FA 20:2	FA 20:2	[M-H]-	C20H36O2	308.2715	2
3.176	305.2490	FA 20:3	FA 20:3	[M-H]-	C20H34O2	306.2559	2
2.762	303.2340	FA 20:4	FA 20:4	[M-H]-	C20H3O22	304.2402	2
2.254	301.2177	FA 20:5	FA 20:5	[M-H]-	C20H30O2	302.2249	2
4.490	325.3114	FA 21:0	FA 21:0	[M-H]-	C21H4O22	326.3185	2
6.026	339.3271	FA 22:0	FA 22:0	[M-H]-	C22H44O2	340.3341	2
5.049	337.3116	FA 22:1	FA 22:1	[M-H]-	C22H4O22	338.3185	2
3.491	331.2647	FA 22:4	FA 22:4	[M-H]-	C22H36O2	332.2715	2
2.985	329.2493	FA 22:5	FA 22:5	[M-H]-	C22H34O2	330.2559	2
2.585	327.2341	FA 22:6	FA 22:6	[M-H]-	C22H3O22	328.2413	2
5.530	367.3580	FA 24:0	FA 24:0	[M-H]-	C24H48O2	368.3652	2
6.105	365.3425	FA 24:1	FA 24:1	[M-H]-	C24H46O2	366.3497	2
12.155	381.3737	FA 25:0	FA 25:0	[M-H]-	C25H50O2	382.3809	2
9.717	395.3900	FA 26:0	FA 26:0	[M-H]-	C26H5O22	396.3972	2
12.213	395.3906	FA 26:0	FA 26:0	[M-H]-	C26H5O22	396.3978	2
11.761	423.4204	FA 28:0	FA 28:0	[M-H]-	C28H56O2	424.4276	2
5.392	409.3113	FA 28:7	FA 28:7	[M-H]-	C28H4O22	410.3185	2
12.010	437.4365	FA 29:0	FA 29:0	[M-H]-	C29H58O2	438.4437	2
12.212	451.4525	FA 30:0	FA 30:0	[M-H]-	C30H60O2	452.4597	2

12.412	465.4684	FA 31:0	FA 31:0	[M-H]-	C31H6O22	466.4756	2
12.612	479.4840	FA 32:0	FA 32:0	[M-H]-	C32H64O2	480.4912	2
11.721	493.4987	FA 33:0	FA 33:0	[M-H]-	C33H66O2	494.5059	2
12.177	535.5457	FA 36:0	FA 36:0	[M-H]-	C36H70O2	536.5529	2
3.267	337.2365	FAHFA 20:2	FAHFA 2:0_18:2	[M-H]-	C20H34O4	338.2457	3
4.151	365.2678	FAHFA 22:2	FAHFA 2:0_20:2	[M-H]-	C22H38O4	366.2770	3
2.861	361.2362	FAHFA 22:4	FAHFA 2:0_20:4	[M-H]-	C22H34O4	362.2457	3
3.988	397.3327	FAHFA 24:0	FAHFA 2:0_22:0	[M-H]-	C24H46O4	398.3396	3
1.320	243.1971	FA(14:0)-O2Ha	FA(14:0)-O2Ha	[M-H]-	C14H28O3	244.2039	2
1.021	329.2355	FA 18:1;O3	9,12,13-TriHOME	[M-H]-	C18H34O5	330.2406	1
2.429	269.2499	FA16:0;O	FA16:0<9O>	[M-H]-	C16H30O3	270.2195	2
2.430	319.2373	FA 18:1;OOH	FA 18:1<9OOH>	[M-H]-	C18H34O4	320.2445	2
2.957	297.2431	FA 18:1;OH	FA 18:1<9OH>	[M-H]-	C18H34O3	298.2503	2
1.304	291.1997	FA 18:4;OH	FA 18:4<12OH>	[M-H]-	C18H28O3	292.2069	2
1.130	271.2282	FA(16:0)-O2Ha	FA(16:0)-O2Ha	[M-H]-	C16 H32 O3	272.2351	2
1.470	299.2598	FA(18:0)-O2Ha	FA(18:0)-O2Ha	[M-H]-	C18 H36 O3	300.2666	2
0.860	213.1496	FA(12:1)-O2Hb	FA(12:1)-O2Hb	[M-H]-	C12 H22 O3	214.1568	2
1.150	269.2127	FA(16:1)-O2Hb	FA(16:1)-O2Hb	[M-H]-	C16 H30 O3	270.2195	2
1.470	297.2444	FA(18:1)-O2Ha	FA(18:1)-O2Ha	[M-H]-	C18 H34 O3	298.2509	2
1.100	211.1343	FA(12:2)-O2Hb	FA(12:2)-O2Hb	[M-H]-	C12 H20 O3	212.1415	2
1.320	243.1967	FA(14:0)-3OHa	FA(14:0)-3OHa	[M-H]-	C14 H28 O3	244.2039	2
1.250	271.2282	FA(16:0)-3OHa	FA(16:0)-3OHa	[M-H]-	C16 H32 O3	272.2350	2
1.620	299.2598	FA(18:0)-3OHa	FA(18:0)-3OHa	[M-H]-	C18 H36 O3	300.2665	2
0.860	213.1499	FA(12:1)-3OHb	FA(12:1)-3OHb	[M-H]-	C12 H22 O3	214.1568	2
1.390	269.2126	FA(16:1)-3OHb	FA(16:1)-3OHb	[M-H]-	C16 H30 O3	270.2195	2
1.660	297.2440	FA(18:1)-3OHb	FA(18:1)-3OHb	[M-H]-	C18 H34 O3	298.2509	2
0.670	291.1961	FA(18:4)-3OHb	FA(18:4)-3OHb	[M-H]-	C18 H28 O3	292.2073	2
1.181	344.2814	Car 12:0	Car 12:0	[M+H]+	C19H37NO4	343.2723	2
1.621	372.3112	Car 14:0	Car 14:0	[M+H]+	C21H41NO4	371.3036	2
1.341	370.2956	Car 14:1	Car 14:1	[M+H]+	C21H39NO4	369.2892	2
2.382	400.3429	Car 16:0	Car 16:0	[M+H]+	C23H45NO4	399.3349	2
1.784	398.3269	Car 16:1	Car 16:1	[M+H]+	C23H43NO4	397.3192	2
1.480	396.3120	Car 16:2	Car 16:2	[M+H]+	C23H43NO4	395.3048	2
3.276	428.3739	Car 18:0	Car 18:0	[M+H]+	C25H49NO4	427.3662	2
2.579	426.3585	Car 18:1	Car 18:1	[M+H]+	C25H47NO4	425.3505	2
2.017	424.3446	Car 18:2	Car 18:2	[M+H]+	C25H45NO4	423.3349	2
4.107	456.4052	Car 20:0	Car 20:0	[M+H]+	C27H53NO4	455.3925	2
3.406	454.3889	Car 20:1	Car 20:1	[M+H]+	C27H51NO4	453.3781	2
2.012	448.3424	Car 20:4	Car 20:4	[M+H]+	C27H45NO4	447.3349	2
2.392	496.3402	Car 22:5	Car 22:5	[M+Na]+	C29H47NO4	473.3505	2
0.769	232.1547	Car 4:0	Car 4:0	[M+H]+	C11H21NO4	231.1471	2

0.796	246.1704	Car 5:0	Car 5:0	[M+H]+	C12H23NO4	245.1627	2
0.806	260.1855	Car 6:0	Car 6:0	[M+H]+	C13H25NO4	259.1784	2
15.063	666.6172	CE 18:2	CE 18:2	[M+NH4]+	C45H76O2	648.5845	2
14.301	714.6186	CE 22:6	CE 22:6	[M+H4N]+	C49H76O2	696.5845	2
