# Supplementary material for: LiLA: lipid lung-based ATLAS built through a comprehensive workflow designed for an accurate lipid annotation
Source: Commun Biol. 2024 Jan 5;7:45. doi: 10.1038/s42003-023-05680-7 (PMC10770321; doi:10.1038/s42003-023-05680-7)
Supplement: Supplementary file 5 — Supplementary Data 3 [file 42003_2023_5680_MOESM5_ESM.pdf]

Supplementary Data 3. Semi-quantification results of the semi-targeted experiment. The amount of each of the 709 lipid species semi-quantified are expressed in ng of lipid per mg of lung tissue. The PC 15:0-18:1(d7) was used to semi-quantified PC and PC O-; Lyso PC 18:1(d7) for LPC, LPI, LPG and LPS lipid subclasses; PE 15:0-18:1(d7) for PE and PE O-; Lyso PE 18:1(d7) for semi-quantified LPE and LPE O-; PG 15:0-18:1(d7), PI 15:0-18:1(d7) and PS 15:0-18:1(d7) were used for PG, PI and PS respectively; TAG 15:0-18:1(d7)-15:0, DAG 15:0-18:1(d7) and MAG 18:1(d7) were used for semi-quantified TG, DG and MG respectively; SM d18:1-18:1(d9) was used for SM, SPB, Cer and HexCer; C17 sphinganine was used for ACar semi-quantification; and PG 15:0-18:1(d7) was used for BMP semi-quantification. The semi-quantification corresponding to the lipids marked with (*) corresponds to more than one lipid species (coelution).

Name	Formula	Mass	RT (min)	ESI Mode	[Area] Sample 1	[Area] Sample 2	[Area] Sample 3	Average	ng/µL	ng Lipid/mg lung tissue
BMP 16:0/16:0	C38H75O10P	722.5083	7.26	ESI(+)	961475	879606	1029925	957002	5.23	219.81
BMP 16:0_20:4	C42H75O10P	770.5082	6.26	ESI(+)	1468438	1474627	1475627	1472897	8.05	338.07
BMP 18:2/18:2	C42H75O10P	770.5091	7.8	ESI(+)	2230686	2527328	2494282	2417432	13.20	554.60
BMP 22:6/22:6	C50H75O10P	866.5095	4.81	ESI(+)	466848	473308	442708	460955	2.53	106.10
Car 12:0	C19H37NO4	343.2722	1.11	ESI(+)	155503	156178	163185	158289	0.08	3.56
Car 14:0	C21H41NO4	371.3033	1.52	ESI(+)	633078	667781	681471	660777	0.15	6.18
Car 14:1	C21H39NO4	369.2878	1.26	ESI(+)	197596	185992	194380	192656	0.09	3.74
Car 16:0	C23H45NO4	399.3348	2.27	ESI(+)	2403555	2377678	2410151	2397128	0.36	15.25
Car 16:1	C23H43NO4	397.3185	1.71	ESI(+)	520451	576932	567453	554945	0.13	5.63
Car 16:2	C23H43NO4	395.3048	1.48	ESI(+)	429505	466416	465204	429504	0.12	5.10
Car 18:0	C25H49NO4	427.3661	3.17	ESI(+)	1400304	1400896	1397725	1399642	0.24	10.04
Car 18:1	C25H47NO4	425.3504	2.46	ESI(+)	1873089	1850059	1881328	1868159	0.30	12.48
Car 18:2	C25H45NO4	423.3340	1.91	ESI(+)	931561	922449	912857	922289	0.18	7.55
Car 20:0	C27H53NO4	455.3958	4.01	ESI(+)	545728	576819	570232	564260	0.14	5.68
Car 20:1	C27H51NO4	453.3847	3.32	ESI(+)	887641	969577	1012318	956512	0.18	7.73
Car 20:4	C27H45NO4	447.3339	1.91	ESI(+)	1352544	1288363	1282199	1307702	0.23	9.56
Car 22:5	C29H47NO4	473.3505	2.392	ESI(+)	25509170	24644100	24160758	24160757	3.14	132.05
Car 4:0	C11H21NO4	231.1482	0.71	ESI(+)	377372	366271	370029	371224	0.11	4.67
Car 5:0	C12H23NO4	245.1627	0.796	ESI(+)	110079	97827	104077	97827	0.08	3.24
Car 6:0	C13H25NO4	259.1805	0.74	ESI(+)	150762	137876	132512	140383	0.08	3.46
Cer 15:0;O2/26:2	C41H79NO3	633.6115	12.19	ESI(+)	5542885	5634174	5570562	5582540	0.90	37.84
Cer 16:0;O2/18:1	C34H67NO3	537.5116	9.35	ESI(+)	2606998	2276626	2657735	2513786	0.44	18.56
Cer 16:0;O2/18:2	C34H65NO3	535.4913	7.6	ESI(+)	3521572	3548772	3496365	3522236	0.59	24.90
Cer 16:0;O2/23:1	C39H77NO3	607.5928	11.94	ESI(+)	8054072	6732424	7836967	7541154	1.19	50.14
Cer 18:0;O2/16:0	C34H69NO3	539.5270	10.25	ESI(+)	629022	604881	590717	608207	0.16	6.59
Cer 18:0;O2/18:0	C36H73NO3	567.5589	11.82	ESI(+)	6151912	1024605	5477719	4218079	0.70	29.27
Cer 18:0;O2/18:1	C36H71NO3	565.5400	11.83	ESI(+)	7640946	7794406	10137765	8524372	1.34	56.32
Cer 18:0;O2/20:0	C38H77NO3	595.5898	11.99	ESI(+)	2484296	2710067	3921472	3038612	0.52	21.86
Cer 18:0;O2/22:0	C40H81NO3	623.6154	11.94	ESI(+)	8398019	8617043	8472320	8495794	1.34	56.14
Cer 18:0;O2/23:0	C41H83NO3	637.6393	12.08	ESI(+)	4022509	3992726	5334777	4450004	0.73	30.73
Cer 18:0;O2/24:0	C42H85NO3	651.6574	12.66	ESI(+)	3223172	3215255	3225492	3221306	0.55	23.01
Cer 18:0;O2/24:1	C42H83NO3	649.6374	12.68	ESI(+)	3400969	3022722	3386110	3269934	0.56	23.31

Cer 18:0;O2/25:0	C43H87NO3	665.6680	12.91	ESI(-)	1513689	1328304	918520	1253504	1.14	47.94
Cer 18:0;O2/25:1	C43H85NO3	663.6550	12.32	ESI(+)	1897450	2026293	2023542	1982428	0.36	15.23
Cer 18:0;O2/26:1	C44H87NO3	677.6689	13.01	ESI(+)	2428446	2440696	2501489	2456877	0.43	18.21
Cer 18:1;O2/14:0	C32H63NO3	509.4833	7.24	ESI(-)	288497	207223	307148	267623	0.03	1.17
Cer 18:1;O2/15:0	C33H65NO3	523.4975	8.24	ESI(+)	669964	627355	650720	649346	0.16	6.85
Cer 18:1;O2/16:0	C34H67NO3	537.5117	9.35	ESI(+)	2503258	2512849	2671599	2562569	0.45	18.87
Cer 18:1;O2/17:0	C35H69NO3	551.5192	10.29	ESI(+)	3285413	3259328	3353763	3299501	0.56	23.50
Cer 18:1;O2/18:0	C36H71NO3	565.5400	11.83	ESI(+)	7657595	7832795	8281673	7924021	1.25	52.55
Cer 18:1;O2/19:0	C37H73NO3	579.5551	11.96	ESI(+)	5724870	6754557	4554744	5678057	0.92	38.44
Cer 18:1;O2/20:0	C38H75NO3	593.5770	12.2	ESI(+)	3407687	8193215	4102513	5234472	0.85	35.65
Cer 18:1;O2/21:0	C39H75NO3	605.5822	11.83	ESI(+)	11353945	10613038	12062610	11343198	1.76	74.02
Cer 18:1;O2/22:0	C40H79NO3	621.6061	12.33	ESI(+)	5664847	2656842	2568924	3630204	0.61	25.58
Cer 18:1;O2/23:0	C41H81NO3	635.6241	12.5	ESI(+)	2891437	2312069	1016272	2073259	0.38	15.80
Cer 18:1;O2/24:0	C42H83NO3	649.6372	12.66	ESI(+)	3822232	3894469	3864865	3860522	0.64	27.02
Cer 18:1;O2/24:1	C42H81NO3	647.6211	12.31	ESI(+)	5985731	5888186	5964612	5946176	0.96	40.12
Cer 18:1;O2/24:2	C42H79NO3	645.6081	12.04	ESI(+)	6792276	3941361	10572975	7102204	1.13	47.39
Cer 18:1;O2/25:0	C43H85NO3	663.6549	12.32	ESI(+)	2057159	2264978	2195949	2172695	0.39	16.42
Cer 18:1;O2/25:1	C43H83NO3	661.6370	12.33	ESI(+)	1428432	2272984	1630808	1777408	0.33	13.94
Cer 18:1;O2/26:0	C44H87NO3	677.6691	12.51	ESI(+)	1654666	1357443	2721684	1911264	0.35	14.78
Cer 18:2;O2/16:0	C34H65NO3	535.4909	7.6	ESI(+)	6055092	6223481	5922855	6067143	0.97	40.88
Cer 18:2;O2/18:0	C36H69NO3	563.5215	9.83	ESI(+)	3906770	3658985	3873179	3812978	0.64	26.72
Cer 18:2;O2/20:0	C38H73NO3	591.5532	11.91	ESI(+)	7576801	4676840	3995891	5416511	0.88	36.80
Cer 18:2;O2/22:0	C40H77NO3	619.5934	12.07	ESI(+)	9481800	9527460	9580821	9530027	1.49	62.63
Cer 18:2;O2/23:0	C41H79NO3	633.6132	12.19	ESI(+)	5445175	5503712	5503139	5484009	0.89	37.22
Cer 18:2;O2/24:0	C42H81NO3	647.6211	12.31	ESI(+)	5985731	5888186	5964612	5946176	0.96	40.12
Cer 18:2;O2/24:1	C42H79NO3	645.6076	12.04	ESI(+)	6792276	3941361	1020321	3917986	0.65	27.38
Cer 18:2;O2/24:2	C42H77NO3	643.5896	11.78	ESI(+)	5920361	5291488	2356788	4522879	0.74	31.18
Cer 18:2;O2/25:0	C43H81NO3	659.6221	12.73	ESI(+)	4902494	4825342	4940027	4889288	0.80	33.49
Cer 18:2;O2/26:1	C44H83NO3	673.6371	12.26	ESI(-)	4024298	3362886	4061416	3816200	4.04	169.51
Cer 19:0;O2/24:2	C43H81NO3	659.6224	12.74	ESI(+)	3686725	1894329	2452417	2677824	0.47	19.59
Cer 19:1;O2/17:0	C36H69NO3	563.5212	11.88	ESI(+)	3168025	3000489	11569137	5912550	0.95	39.91
CL 16:0_18:0_22:4_22:6	C87H150O17P2	1529.036	7.78	ESI(+)	244552	289344	324991	286296	0.90	37.89
DG 14:0_18:1	C35H66O5	566.4905	11.7	ESI(+)	2644722	2595016	2608877	2616205	0.97	40.89
DG 16:0_16:0	C35H68O5	568.5063	11.91	ESI(+)	1211689	1145192	1216328	1191070	0.71	29.67
DG 16:0_16:1	C35H66O5	566.4902	11.51	ESI(+)	2420418	2373679	2346592	2380230	0.93	39.03
DG 16:0_18:1	C37H70O5	594.5224	11.62	ESI(+)	1139662	1027837	1112716	1093405	0.69	28.91
DG 16:0_18:2	C37H68O5	592.4990	11.85	ESI(+)	5488725	5602811	5574648	5555395	1.52	64.02
DG 16:0_20:3	C39H70O5	618.5231	11.47	ESI(+)	9361886	9073874	9377831	9271197	2.22	93.26
DG 16:0_20:4	C39H68O5	616.5060	11.48	ESI(+)	2508969	2525414	2432802	2489062	0.95	39.89
DG 16:0_22:4	C41H72O5	644.5372	12.24	ESI(+)	675685	672098	741847	696543	0.61	25.78
DG 16:0_22:5	C41H70O5	642.5208	11.37	ESI(+)	1668295	1628917	1604060	1633757	0.79	33.16
DG 16:0_22:6	C41H68O5	640.5059	11.2	ESI(+)	2233128	2260273	2273721	2255707	0.91	38.05
DG 16:1_16:0	C35H66O5	566.4907	11.83	ESI(+)	2001812	2156963	1981162	2046646	0.87	36.41
DG 16:1_18:1	C37H68O5	592.4983	11.34	ESI(+)	3792234	3664399	3749812	3735482	1.18	49.70
DG 16:1_18:2	C37H66O5	590.4898	10.06	ESI(+)	6901536	6629184	6844299	6791673	1.76	73.75
DG 17:0_20:4	C40H70O5	630.5223	11.77	ESI(+)	3241557	3259646	3162362	3221188	1.09	45.65
DG 18:0_18:1	C39H74O5	622.5540	11.93	ESI(+)	7524598	7474905	7359328	7452944	1.88	78.95

DG 18:0_18:2	C39H72O5	620.5476	11.98	ESI(+)	8417865	2037151	2023397	4159471	1.26	53.03
DG 18:0_20:3	C41H74O5	646.5503	11.94	ESI(+)	4359934	4346507	4258960	4321800	1.29	54.31
DG 18:0_20:4	C41H72O5	644.5379	11.94	ESI(+)	9487118	9609970	9401310	9499466	2.26	95.05
DG 18:0_22:4	C43H76O5	672.5696	12.15	ESI(+)	2354034	2409856	2397882	2387257	0.93	39.09
DG 18:0_22:5	C43H74O5	670.5470	12.66	ESI(+)	1036967	1082853	1035371	1051730	0.68	28.58
DG 18:0_22:6	C43H72O5	668.5365	11.88	ESI(+)	5185260	5761964	5073091	5340105	1.48	62.32
DG 18:1_16:0	C37H70O5	594.5219	11.94	ESI(+)	2240022	2187500	2270110	2232544	0.90	37.87
DG 18:1_18:1	C39H72O5	620.5462	11.98	ESI(+)	5727100	5622516	5593059	5647558	1.54	64.74
DG 18:1_18:2	C39H70O5	618.5215	11.68	ESI(+)	13639805	13254817	13609720	13501447	3.01	126.55
DG 18:1_18:3	C39H68O5	616.5060	10.84	ESI(+)	467166	466318	484437	472640	0.57	24.02
DG 18:1_20:3	C41H72O5	644.5381	11.94	ESI(+)	8334838	9098618	8868603	8767353	2.13	89.29
DG 18:1_20:4	C41H70O5	642.5214	11.56	ESI(+)	2911915	2885043	2807509	2868156	1.02	42.87
DG 18:1_22:4	C43H74O5	670.5530	11.96	ESI(+)	2937589	2856135	2808422	2867382	1.02	42.87
DG 18:1_22:6	C43H70O5	666.5262	11.94	ESI(+)	7590202	7440382	7513271	7514618	1.89	79.44
DG 18:2_16:0	C37H68O5	592.4988	11.63	ESI(+)	15553598	15815877	15637954	15669143	3.42	143.61
DG 18:2_18:0	C39H72O5	620.5483	11.68	ESI(+)	3855892	3747251	3808416	3803853	1.20	50.23
DG 18:2_18:1	C39H70O5	618.5224	11.51	ESI(+)	6271170	6281953	6343584	6298902	1.66	69.87
DG 18:2_18:2	C39H68O5	616.5060	10.73	ESI(+)	1325618	1345213	1201455	1290762	0.73	30.46
DG 18:2_18:3	C39H66O5	614.4811	9.18	ESI(+)	2986537	2962883	3064710	3004710	1.05	43.95
DG 18:2_22:6	C43H68O5	664.4978	9.69	ESI(+)	1522842	670717	927242	1040267	0.68	28.49
DG 20:1_20:4	C43H74O5	670.5484	11.33	ESI(+)	827915	914633	857469	866672	0.65	27.12
DG 20:4_20:4	C43H68O5	664.4945	9.73	ESI(+)	5567573	5320513	5526997	5471694	1.51	63.36
DG 22:4_18:1	C43H74O5	670.5511	11.61	ESI(+)	1816500	1789226	1826192	1810639	0.82	34.55
FA 14:0	C14H28O2	228.2090	2.52	ESI(-)	6962490	6867790	6658734	6829671	7.44	312.53
FA 15:0	C15H30O2	242.2246	2.55	ESI(-)	1403842	1483674	1198505	1362007	1.02	42.64
FA 16:1	C16H30O2	254.2244	2.74	ESI(-)	1573061	1639972	304729	1172587	0.79	33.29
FA 17:0	C17H34O2	270.2558	3.85	ESI(-)	5663918	5520450	5375589	5519986	5.90	247.88
FA 18:1	C18H34O3	298.2507	1.52	ESI(-)	3353449	3411190	3384860	3383166	3.39	142.40
FA 18:3	C18H30O2	278.2242	2.5	ESI(-)	612064	612495	634184	619581	0.14	5.99
FA 19:0	C19H38O2	298.2871	4.65	ESI(-)	2445947	2370358	2167764	2328023	2.15	90.32
FA 20:0	C20H40O2	312.3027	5.09	ESI(-)	7759010	7544237	7545064	7616104	8.37	351.35
FA 20:1	C20H38O2	310.2871	4.36	ESI(-)	1611913	754903	1835870	1400895	1.06	44.56
FA 20:3	C20H34O2	306.2555	3.3	ESI(-)	579859	505308	1037488	707552	0.25	10.33
FA 20:5	C20H30O2	302.2244	2.39	ESI(-)	1011108	969710	1050298	1010372	0.60	25.28
FA 22:0	C22H44O2	340.3340	6.19	ESI(-)	4145223	4071463	4343697	4186794	4.34	182.07
FA 22:4	C22H36O2	332.2719	3.6	ESI(-)	522261	507022	566288	531857	0.04	1.66
FA 22:5	C22H34O2	330.2558	3.1	ESI(-)	1083777	1025411	967018	1025402	0.62	26.02
FA 24:0	C24H48O2	368.3652	5.57	ESI(-)	895309	930054	797188	874184	0.44	18.56
FA 24:1	C24H46O2	366.3495	6.22	ESI(-)	979798	959901	979758	973152	0.56	23.44
FA 25:0	C25H50O2	382.3806	12.04	ESI(-)	4889687	4722571	4405515	4672591	4.91	206.05
FA 28:0	C28H56O2	424.4280	11.82	ESI(-)	9179694	9260669	6384341	8274901	9.14	383.87
FA 30:0	C30H60O2	452.4592	12.24	ESI(-)	6225311	6601208	6636326	6487615	7.04	295.64
FA 32:0	C32H64O2	480.4904	12.62	ESI(-)	3873946	3797834	3863922	3845234	3.93	165.21
FA 36:0	C36H70O2	534.5373	11.97	ESI(-)	3067018	3023762	3154390	3081723	3.04	127.52
FA(12:1)-3OHb	C12H22O3	214.1602	1.06	ESI(+)	182544	197721	187791	189352	0.09	3.72
FA(12:1)-O2Hb	C12H22O3	214.1587	0.82	ESI(+)	289737	285295	274642	283225	0.10	4.21
FA(12:2)-O2Hb	C12H20O3	212.1424	0.81	ESI(+)	566209	581316	573851	573792	0.14	5.73

FA(14:0)-3OHa	C14H28O3	244.2038	1.29	ESI(+)	1944097	1881559	1942477	1922711	0.30	12.77
FA(14:0)-O2Ha	C14H28O3	244.2081	0.82	ESI(+)	2344841	2436362	2301230	2360811	0.36	15.06
FA(16:0)-3OHa	C16H32O3	272.2376	1.34	ESI(+)	675237	714139	706587	698654	0.15	6.38
FA(16:0)-O2Ha	C16H32O3	272.2382	0.94	ESI(+)	423396	470900	432670	442322	0.12	5.04
FA(16:1)-3OHb	C16H30O3	270.2207	1.51	ESI(+)	604641	586001	581956	590866	0.14	5.82
FA(16:1)-O2Hb	C16H30O3	270.2203	0.94	ESI(+)	565128	572958	596415	578167	0.14	5.75
FA(18:0)-3OHa	C18H36O3	300.2663	2.09	ESI(+)	476620	427579	446604	450268	0.12	5.08
FA(18:1)-3OHb	C18H34O3	298.2514	1.71	ESI(+)	549100	544807	537889	543932	0.13	5.57
FA(18:1)-O2Ha	C18H34O3	298.2515	1.41	ESI(+)	252005	246665	254145	250938	0.10	4.04
FA(18:4)-3OHb	C18H28O3	292.2037	1.51	ESI(+)	616610	564817	627227	602885	0.14	5.88
FAHFA 2:0_18:2	C20H34O4	338.2435	3.42	ESI(-)	1480453	1404048	1339326	1407942	1.07	44.90
FAHFA 2:0_20:2	C22H38O4	366.2748	4.27	ESI(-)	1972958	3175615	3002099	2716891	2.61	109.52
FAHFA 2:0_20:4	C22H34O4	362.2456	3.00	ESI(-)	674010	614881	608367	632419	0.16	6.62
FAHFA 2:0_22:0	C24H46O4	398.3395	4.09	ESI(-)	1150696	1125369	1093513	1123193	0.73	30.85
HexCer 18:0;O2/22:1	C40H77NO8	699.5638	7.66	ESI(+)	1368483	1475834	1342889	1395735	0.27	11.54
HexCer 18:1;O2/16:0	C46H89NO8	783.6525	12.20	ESI(+)	5993373	7721244	7425433	7046683	1.12	47.04
HexCer 18:1;O2/22:0	C46H89NO8	783.6525	12.22	ESI(+)	7698936	7681090	7272913	7550980	1.20	50.20
HexCer 18:1;O2/24:0	C48H93NO8	811.6912	12.01	ESI(+)	33098872	34321058	33359733	33593221	5.09	213.78
HexCer 18:1;O2/24:1	C48H91NO8	809.6703	11.69	ESI(+)	16574171	7586000	15657853	13272675	2.05	86.14
LPC 0:0/14:0	C22H46NO7P	467.2993	1.54	ESI(+)	170157	176075	156289	167507	0.09	3.61
LPC 0:0/15:0	C23H46NO7P	479.3086	2.31	ESI(+)	860301	906166	793742	853403	0.17	7.19
LPC 0:0/16:0	C24H50NO7P	495.3322	2.31	ESI(+)	3235804	3273752	2959108	3156221	0.46	19.21
LPC 0:0/16:1	C24H48NO7P	493.3166	1.69	ESI(+)	336147	390884	291143	339391	0.11	4.50
LPC 0:0/18:0	C26H54NO7P	523.3637	3.19	ESI(+)	2173630	2211186	2029579	2138132	0.33	13.89
LPC 0:0/18:1	C26H52NO7P	521.3479	2.49	ESI(+)	1453556	1460680	1314003	1409413	0.24	10.09
LPC 0:0/18:2	C26H50NO7P	519.3296	1.92	ESI(+)	1855902	1712766	1759111	1775926	0.29	12.00
LPC 0:0/20:2	C28H54NO7P	547.3579	2.68	ESI(+)	374463	375664	387199	379109	0.11	4.71
LPC 0:0/20:3	C28H52NO7P	545.3497	1.81	ESI(+)	362037	343827	225474	310446	0.10	4.35
LPC 0:0/20:4	C28H50NO7P	543.3321	1.89	ESI(+)	824857	867406	743786	812016	0.17	6.97
LPC 0:0/20:5	C28H48NO7P	541.3189	1.5	ESI(+)	150628	140453	112425	134502	0.08	3.43
LPC 0:0/22:4	C30H54NO7P	571.3637	2.51	ESI(+)	187935	195215	170810	184653	0.09	3.70
LPC 0:0/22:5	C30H52NO7P	569.3475	2.04	ESI(+)	466148	438885	463739	456257	0.12	5.11
LPC 0:0/22:6	C30H50NO7P	567.3322	1.92	ESI(+)	1588974	1578185	1561735	1576298	0.26	10.96
LPC 0:0/24:0	C36H66NO7P	655.4592	3.08	ESI(+)	651070	647710	667940	655573	0.15	6.15
LPC 14:0/0:0	C22H46NO7P	467.2999	1.67	ESI(+)	594417	572316	580898	582544	0.14	5.77
LPC 15:0/0:0	C23H46NO7P	479.3091	2.49	ESI(+)	5803866	5581177	5504685	5629909	0.76	32.12
LPC 16:0/0:0	C24H50NO7P	495.3327	2.49	ESI(+)	21694594	20740702	20630816	21022037	2.68	112.47
LPC 16:1/0:0	C24H48NO7P	493.3165	1.84	ESI(+)	1626323	1537486	1590178	1584662	0.26	11.00
LPC 17:0/0:0	C25H52NO7P	509.3474	2.94	ESI(+)	1082341	1097997	1125037	1101792	0.20	8.48
LPC 18:0/0:0	C26H54NO7P	523.3638	3.38	ESI(+)	15008329	14427109	14393583	14609674	1.88	79.00
LPC 18:1/0:0	C26H52NO7P	521.348	2.66	ESI(+)	6882984	6603021	6584669	6690225	0.90	37.66
LPC 18:2/0:0	C26H50NO7P	519.3318	2.09	ESI(+)	8002037	7641060	7795247	7812781	1.04	43.52
LPC 18:3/0:0	C26H48NO7P	517.314	2.49	ESI(+)	1766501	1683520	1701579	1717200	0.28	11.70
LPC 19:0/0:0	C27H56NO7P	537.3792	3.79	ESI(+)	856476	799192	819583	825084	0.17	7.04
LPC 20:0/0:0	C28H58NO7P	551.3955	4.18	ESI(+)	1110688	1047667	1080066	1079474	0.20	8.37
LPC 20:1/0:0	C28H56NO7P	549.3785	3.49	ESI(+)	865007	800109	870014	845043	0.17	7.14
LPC 20:2/0:0	C28H54NO7P	547.3593	2.89	ESI(+)	784082	774651	783530	780754	0.16	6.81

LPC 20:3/0:0	C28H52NO7P	545.3486	1.92	ESI(+)	2044696	1911411	1995870	1983992	0.31	13.09
LPC 20:4/0:0	C28H50NO7P	543.3327	2.02	ESI(+)	3336060	3116249	3213287	3221865	0.47	19.55
LPC 20:5/0:0	C28H48NO7P	541.3164	1.61	ESI(+)	414309	415673	416075	415352	0.12	4.90
LPC 22:4/0:0	C30H54NO7P	571.3645	2.69	ESI(+)	725924	726784	729980	727563	0.16	6.53
LPC 22:5/0:0	C30H52NO7P	569.3474	2.19	ESI(+)	930871	787859	877222	865317	0.17	7.25
LPC 22:6/0:0	C30H50NO7P	567.3325	1.81	ESI(+)	315473	318325	252187	295328	0.10	4.27
LPC 24:0/0:0	C32H66NO7P	607.4581	3.12	ESI(+)	1021468	985977	995103	1000849	0.19	7.96
LPE 0:0/20:4	C25H44NO7P	501.2857	1.92	ESI(+)	1479420	1522184	1376533	1459379	0.93	38.89
LPE 0:0/22:4	C27H48NO7P	529.316	2.66	ESI(+)	789870	738297	767974	765380	0.21	8.71
LPE 0:0/22:5	C27H46NO7P	527.3055	2.36	ESI(+)	478896	1053349	978827	837024	0.28	11.83
LPE 0:0/22:6	C27H44NO7P	525.286	1.87	ESI(+)	642192	650055	673652	655300	0.09	3.93
LPE 16:0/0:0	C21H44NO7P	453.2851	2.61	ESI(+)	643392	619961	740100	667818	0.11	4.47
LPE 18:0/0:0	C23H48NO7P	481.3168	3.49	ESI(+)	567007	671596	534101	590901	0.03	1.13
LPE 18:1	C23H46NO7P	479.3067	2.51	ESI(+)	2239638	2550113	2493499	2427750	1.93	80.99
LPE 20:4/0:0	C25H44NO7P	501.2856	2.09	ESI(+)	2378882	2205858	2190735	2258492	1.75	73.63
LPE 22:4/0:0	C27H48NO7P	529.3156	2.89	ESI(+)	978774	939329	1009779	975961	0.43	17.87
LPE 22:5/0:0	C27H46NO7P	527.3014	2.37	ESI(+)	1009856	950147	932417	964140	0.41	17.35
LPE 22:6/0:0	C27H44NO7P	525.2856	2.02	ESI(+)	2643729	2579696	2491841	2571755	2.08	87.25
LPE O-16:1; P-16:0	C21H44NO6P	437.2906	3.01	ESI(+)	623195	675965	658924	652695	0.09	3.81
LPE O-18:1: P-18:0	C23H48NO6P	465.3212	3.84	ESI(+)	748126	768038	725776	747313	0.19	7.93
LPG 16:0/0:0	C22H45O9P	484.2797	2.07	ESI(+)	560556	519835	512728	531040	2.91	122.16
LPI 18:0/0:0	C27H53O12P	600.3252	2.72	ESI(+)	368994	386169	412134	389099	0.12	5.02
LPS 18:0	C24H48NO9P	525.3064	2.84	ESI(-)	810801	813693	802343	808946	0.69	28.99
MG 18:1	C21H40O4	356.2923	3.31	ESI(+)	198319	188640	214628	200529	0.19	8.15
MG 18:2	C23H44O4	384.3222	2.24	ESI(+)	99079	202275	96676	132677	0.16	6.77
MG 20:1	C21H38O4	354.277	1.52	ESI(+)	594182	627287	587935	603135	0.39	16.32
MG 20:2	C23H42O4	382.3084	2.27	ESI(+)	1624420	1606537	1613610	1614856	0.88	36.86
MG 20:3	C23H40O4	380.2934	1.71	ESI(+)	502719	563978	553517	540071	0.36	15.04
MG 20:4	C23H38O4	378.2766	2.93	ESI(+)	557902	577534	592742	576059	0.38	15.77
MG 22:3	C25H44O4	408.3238	2.46	ESI(+)	1480893	1287818	1335711	1368141	0.76	31.85
MG 22:3	C25H44O4	408.3238	4.36	ESI(+)	165660	358856	130455	218324	0.20	8.51
MG 22:6	C25H38O4	402.277	2.82	ESI(+)	344064	313974	300948	319662	0.25	10.57
PC 13:0_22:6	C43H74NO8P	763.5138	8.11	ESI(+)	8234451	8828853	8487653	8516986	2.47	103.81
PC 14:0/16:0	C38H76NO8P	705.5306	7.66	ESI(+)	6992507	7611345	7310710	7304854	2.19	91.80
PC 15:0_16:0	C39H78NO8P	719.5462	8.36	ESI(+)	12295183	12064387	12029609	12129726	3.32	139.59
PC 15:0_18:2	C41H78NO8P	743.5461	10.99	ESI(+)	4637680	4551922	4663296	4617633	1.55	65.19
PC 16:0/14:0	C38H76NO8P	705.531	7.43	ESI(+)	55898231	55134393	51282104	54104909	13.22	555.36
PC 16:0/16:0	C40H80NO8P	733.5626	9.48	ESI(+)	3.15E+08	3.16E+08	3.18E+08	316743514	75.16	3156.80
PC 16:0/16:1	C40H78NO8P	731.5468	7.68	ESI(+)	1.22E+08	1.19E+08	1.15E+08	118590256	28.43	1194.09
PC 16:0/18:0	C42H84NO8P	761.5903	11.88	ESI(+)	2772770	2627835	2607609	2669405	1.09	45.89
PC 16:0/18:1	C42H82NO8P	759.5781	9.77	ESI(+)	1.67E+08	1.63E+08	1.58E+08	162600362	38.81	1630.01
PC 16:0/18:2	C42H80NO8P	757.5625	8.13	ESI(+)	1.36E+08	1.32E+08	1.33E+08	133727745	32.00	1344.03
PC 16:0_15:0	C39H78NO8P	719.5455	8.09	ESI(+)	2504457	2393068	2390946	2429490	1.04	43.51
PC 16:0_20:2	C44H84NO8P	785.593	10.19	ESI(+)	13816155	13150327	12630679	13199054	3.58	150.19
PC 16:0_20:4	C44H80NO8P	781.5625	7.85	ESI(+)	47969473	46598125	44939721	46502440	11.43	480.06
PC 16:0_20:5	C44H78NO8P	779.547	6.83	ESI(+)	7539678	7458261	7256354	7418098	2.21	92.92
PC 16:0_22:4	C46H84NO8P	809.5931	9.4	ESI(+)	6112391	5968221	6043458	6041357	1.89	79.29

PC 16:0_22:5	C46H82NO8P	807.5775	8.01	ESI(+)	9994327	9802228	9516291	9770949	2.77	116.23
PC 16:0_22:6	C46H80NO8P	805.5624	7.46	ESI(+)	44457997	43910089	40317379	42895155	10.58	444.33
PC 16:1/16:0	C40H78NO8P	731.5439	8.71	ESI(+)	5860269	5886527	5963770	5903522	1.86	77.92
PC 18:0_16:0	C42H84NO8P	761.5936	11.56	ESI(+)	22905903	22844784	23032338	22927675	5.87	246.55
PC 18:0_18:1	C44H86NO8P	787.6093	11.64	ESI(+)	32001694	32800157	31636304	32146052	8.04	337.86
PC 18:0_18:2	C44H84NO8P	785.5931	10.19	ESI(+)	13683560	13186558	12577452	13149190	3.56	149.69
PC 18:0_20:2	C46H88NO8P	813.6251	11.73	ESI(+)	5324027	5234055	5361153	5306412	1.71	72.01
PC 18:0_20:4	C46H84NO8P	809.5935	10.09	ESI(+)	26150436	25333539	25115886	25533287	6.48	272.36
PC 18:0_20:5	C46H82NO8P	807.5779	8.61	ESI(+)	3064271	3042299	2710781	2939117	1.16	48.56
PC 18:1/16:0	C42H82NO8P	759.5775	9.98	ESI(+)	34145135	35712381	36848241	35568586	8.85	371.76
PC 18:1/18:1	C44H84NO8P	785.5934	10.5	ESI(+)	46192772	45517069	44875546	45528462	11.20	470.41
PC 18:1/22:6	C48H82NO8P	831.5771	7.66	ESI(+)	2983638	2989531	2933343	2968837	1.16	48.85
PC 18:1_18:2	C44H82NO8P	783.5769	8.33	ESI(+)	8541848	8179437	8014821	8245369	2.41	101.12
PC 18:1_20:4	C46H82NO8P	807.5776	8.01	ESI(+)	10983043	10613896	10537168	10711369	2.99	125.54
PC 18:2/14:0	C40H76NO8P	729.5306	6.5	ESI(+)	10726442	10468950	10125192	10440195	2.93	122.86
PC 18:2/16:0	C42H80NO8P	757.5621	8.28	ESI(+)	27328239	27540437	28710955	27859877	7.03	295.40
PC 18:2/18:0	C44H84NO8P	785.5934	10.5	ESI(+)	43957444	43227127	42543666	43242746	10.66	447.77
PC 18:2/18:2	C44H80NO8P	781.5615	7.05	ESI(+)	1688787	1245260	1800756	1578268	0.84	35.08
PC 18:2_14:0	C40H76NO8P	729.5308	6.69	ESI(+)	2171776	1934036	1856025	1987279	0.93	39.13
PC 18:2_18:1	C44H82NO8P	783.577	8.33	ESI(+)	8376468	8188865	8031668	8199000	2.40	100.66
PC 18:2_18:2	C44H80NO8P	781.5612	7.04	ESI(+)	2519663	2429022	2418135	2455607	1.04	43.77
PC 19:2_18:3	C45H80NO8P	793.5619	7.36	ESI(+)	934763	917347	894990	915700	0.68	28.52
PC 20:0_16:0	C44H88NO8P	789.6251	12.11	ESI(+)	3703525	3213027	2380873	3099142	1.19	50.15
PC 20:0_18:2	C46H88NO8P	813.6218	11.19	ESI(+)	2442146	2358416	2425370	2408644	1.03	43.31
PC 20:1_18:1	C46H88NO8P	813.6256	11.73	ESI(+)	8903298	8782394	8925186	8870293	2.55	107.31
PC 20:4_18:1	C46H82NO8P	807.5772	8.61	ESI(+)	4040749	3933938	3878087	3950925	1.39	58.58
PC 22:0_16:0	C46H92NO8P	817.6576	12.01	ESI(+)	14272203	14155233	14383510	14270315	3.83	160.80
PC 22:6/22:6	C52H80NO8P	877.5637	5.86	ESI(+)	407480	412158	401978	407205	0.56	23.48
PC 22:6_16:0	C46H80NO8P	805.5619	7.61	ESI(+)	8335382	8392495	8730569	8486149	2.46	103.50
PC 28:0	C36H72NO8P	677.499	6.03	ESI(+)	2344299	2250515	2321484	2305433	1.01	42.28
PC 29:0	C37H74NO8P	691.5142	6.66	ESI(+)	1815487	1671430	1759694	1748870	0.88	36.77
PC 30:1	C38H74NO8P	703.5145	6.19	ESI(+)	5287105	5060971	5031516	5126531	1.67	70.23
PC 32:3	C40H74NO8P	727.5153	6.26	ESI(+)	6093278	5894561	5984920	5990920	1.88	78.79
PC 33:0	C41H82NO8P	747.5764	10.86	ESI(+)	4091049	4086709	3975766	4051175	1.42	59.58
PC 33:0	C41H82NO8P	747.5764	10.36	ESI(+)	4163295	3998366	3988725	4050129	1.42	59.56
PC 33:1	C41H80NO8P	745.5596	8.63	ESI(+)	3633371	3811055	3577942	3674123	1.33	55.84
PC 33:4	C41H74NO8P	739.5149	8.13	ESI(+)	93419588	90406432	89533466	91119829	21.95	921.99
PC 34:3	C42H78NO8P	755.5462	7	ESI(+)	2713446	2656607	2474777	2614943	1.08	45.35
PC 34:3	C42H78NO8P	755.5462	7.01	ESI(+)	1871505	1743860	1611978	1742448	0.87	36.71
PC 34:4	C42H76NO8P	753.5282	6.33	ESI(+)	807433	874998	825108	835846	0.66	27.73
PC 35:0	C43H86NO8P	775.6087	12.06	ESI(+)	4466478	4373707	4275265	4371817	1.49	62.75
PC 35:1	C43H84NO8P	773.5931	11.14	ESI(+)	4372848	4365083	4404332	4380754	1.50	62.84
PC 35:2	C43H82NO8P	771.5773	9.16	ESI(+)	2908775	2958361	2977386	2948174	1.16	48.65
PC 35:5	C43H76NO8P	765.5336	7.45	ESI(+)	1081138	1211648	948073	1080286	0.72	30.15
PC 36:0	C44H88NO8P	789.627	11.2	ESI(+)	6452184	6242604	6249026	6314605	1.95	81.99
PC 36:3	C44H82NO8P	783.5738	7.83	ESI(+)	9714137	9713242	9811652	9746344	2.76	115.99
PC 36:5	C44H78NO8P	779.5467	6.83	ESI(+)	6369414	6044834	5340414	5918221	1.86	78.07

PC 36:6	C44H76NO8P	777.5312	6.07	ESI(+)	1576405	1633254	1576547	1595402	0.84	35.25
PC 37:4	C45H82NO8P	795.5771	8.86	ESI(+)	1599419	1620663	1596469	1605517	0.84	35.35
PC 37:6	C45H78NO8P	791.5465	10.08	ESI(+)	25983390	25548215	25064360	25531988	6.48	272.34
PC 37:7	C45H76NO8P	789.533	8.03	ESI(+)	9770734	9585044	9462517	9606098	2.73	114.60
PC 37:7	C45H76NO8P	789.529	11.72	ESI(+)	1409618	1331251	1402442	1381104	0.79	33.13
PC 38:1	C46H90NO8P	815.6411	11.68	ESI(+)	8938784	8942330	9020842	8967319	2.58	108.27
PC 38:3	C46H86NO8P	811.6143	11.2	ESI(+)	9640676	9656302	9568543	9621840	2.73	114.75
PC 38:4	C46H84NO8P	809.5914	8.76	ESI(+)	2009109	1923694	1861429	1931411	0.92	38.58
PC 38:6	C46H80NO8P	805.562	6.82	ESI(+)	1633254	1561652	1579034	1591313	0.84	35.21
PC 38:7	C46H78NO8P	803.5454	6.42	ESI(+)	1968125	1890995	1829123	1896081	0.91	38.23
PC 39:0	C47H94NO8P	831.6704	11.66	ESI(+)	2375889	2371906	2531199	2426331	1.04	43.48
PC 39:4	C47H86NO8P	823.6088	11.32	ESI(+)	785432	654966	583847	674748	0.62	26.13
PC 39:6	C47H82NO8P	819.5877	8.38	ESI(+)	3563522	3478675	3424913	3489037	1.29	54.01
PC 40:0	C48H96NO8P	845.687	12.75	ESI(+)	891358.5	703837	1078880	891359	0.67	28.28
PC 40:1	C48H94NO8P	843.6718	12.38	ESI(+)	2441646	2399885	2414784	2418772	1.03	43.41
PC 40:2	C48H92NO8P	841.6567	12.06	ESI(+)	13246037	13386304	12979466	13203936	3.58	150.23
PC 40:3	C48H90NO8P	839.6401	11.76	ESI(+)	1891441	1738373	1948774	1859529	0.90	37.87
PC 40:4	C48H88NO8P	837.6244	11.53	ESI(+)	5286091	5319697	5309468	5305085	1.71	72.00
PC 40:5	C48H86NO8P	835.6086	10.33	ESI(+)	5307274	5092319	4993868	5131154	1.67	70.27
PC 40:6	C48H84NO8P	833.5943	9.5	ESI(+)	10990815	10604470	10614863	10736716	3.00	125.80
PC 40:6	C48H84NO8P	833.6038	8.64	ESI(+)	2017226	1957614	1970690	1981843	0.93	39.08
PC 40:7	C48H82NO8P	831.5771	9.48	ESI(+)	1664349	1751079	1783135	1732854	0.87	36.61
PC 40:8	C48H80NO8P	829.5615	6.5	ESI(+)	892960	872223	807727	857637	0.67	27.94
PC 42:1	C50H98NO8P	871.7029	12.78	ESI(+)	2553584	2573809	2575628	2567674	1.07	44.88
PC 42:10	C50H80NO8P	853.5706	6.31	ESI(+)	789313	788539	790921	789591	0.65	27.27
PC 42:2	C50H96NO8P	869.6875	12.47	ESI(+)	4165200	3962057	3974537	4033931	1.41	59.40
PC 42:3	C50H94NO8P	867.6725	12.08	ESI(+)	6610491	6604844	6480946	6565427	2.01	84.48
PC 42:4	C50H92NO8P	865.6554	11.89	ESI(+)	4902918	4971949	4920559	4931809	1.63	68.30
PC 42:5	C50H90NO8P	863.6403	11.63	ESI(+)	3356494	3468242	3442294	3422343	1.27	53.35
PC O-16:1_20:4	C44H80NO7P	765.5669	8.73	ESI(+)	15036931	14440758	14376031	14617907	3.91	164.24
PC O-18:1_16:0	C42H84NO7P	745.5982	11.33	ESI(+)	5588269	5858325	6011161	5819252	1.84	77.09
PC O-30:0	C38H78NO7P	691.5515	8.65	ESI(+)	763911	587879	575985	642592	0.61	25.81
PC O-30:1	C38H76NO7P	689.534	8.32	ESI(+)	1082878	1141408	1086245	1103510	0.72	30.38
PC O-32:0	C40H82NO7P	719.582	11.2	ESI(+)	7876935	7489727	7438705	7601789	2.26	94.74
PC O-32:1	C40H80NO7P	717.567	10.85	ESI(+)	24822509	24407188	23690184	24306627	6.20	260.21
PC O-34:0	C42H86NO7P	747.6137	11.91	ESI(+)	5858441	5710901	5694252	5754531	1.82	76.45
PC O-34:2	C42H82NO7P	743.5825	9.78	ESI(+)	16871903	17107011	16775194	16918036	4.45	187.02
PC O-36:1	C44H88NO7P	773.6314	11.2	ESI(+)	8633953	8414052	8461254	8503086	2.47	103.67
PC O-36:2	C44H86NO7P	771.6203	11.64	ESI(+)	4013423	5532329	3568006	4371253	1.49	62.75
PC O-36:4	C44H82NO7P	767.5852	9.25	ESI(+)	13445425	13372490	13206530	13341482	3.61	151.60
PC O-37:6	C45H80NO7P	777.5674	11.53	ESI(+)	5978281	6055913	5991042	6008412	1.88	78.96
PC O-38:4	C46H86NO7P	795.6136	11.19	ESI(+)	3105035	3267353	2951169	3107852	1.20	50.23
PC O-38:6	C46H82NO7P	791.5813	8.63	ESI(+)	4090414	3801911	3770022	3887449	1.38	57.95
PC O-38:7	C46H80NO7P	789.5706	8.28	ESI(+)	3131883	3258968	2791327	3060726	1.18	49.76
PC O-40:4	C48H90NO7P	823.6449	11.84	ESI(+)	7983854	8042241	8193106	8073067	2.37	99.41
PC O-40:7	C48H84NO7P	817.5992	8.96	ESI(+)	3172073	3108166	3071369	3117203	1.20	50.32
PC O-40:8	C48H82NO7P	815.5839	9.49	ESI(+)	12447349	12491037	12381949	12440112	3.40	142.67

PE 16:0_16:1	C37H72NO8P	689.4994	8	ESI(+)	275448	297178	322442	298356	0.23	9.76
PE 16:0_18:1	C39H76NO8P	717.5306	10.07	ESI(-)	1783630	1853658	1981616	1872968	1.73	72.67
PE 16:0_18:2	C39H74NO8P	715.5147	8.48	ESI(+)	2334001	2214620	2173187	2240603	2.16	90.64
PE 16:0_20:4	C41H74NO8P	739.514	8.34	ESI(+)	905212	1027964	986104	973093	0.90	37.86
PE 16:0_20:5	C41H72NO8P	737.4995	7.09	ESI(+)	1491482	1478194	1480763	1483480	1.41	59.11
PE 16:0_22:5	C43H76NO8P	765.5279	8.46	ESI(+)	923406	882922	922216	909515	0.84	35.21
PE 16:0_22:6	C43H74NO8P	763.5146	7.75	ESI(-)	3896980	3930936	3962893	3930270	3.40	142.80
PE 18:0_18:1	C41H80NO8P	745.5618	11.67	ESI(-)	2642290	2651858	2677036	2657061	2.37	99.40
PE 18:0_18:2	C41H78NO8P	743.5459	10.84	ESI(-)	1875428	1834830	1794881	1835046	1.70	71.38
PE 18:0_20:3	C43H80NO8P	769.5619	11.41	ESI(+)	1699904	1720939	1692743	1704529	1.63	68.31
PE 18:0_20:4	C43H78NO8P	767.5459	10.46	ESI(-)	2972964	2982053	2909469	2954829	2.61	109.55
PE 18:0_22:4	C45H82NO8P	795.5772	11.61	ESI(-)	2829954	2193369	2068190	2363838	2.13	89.40
PE 18:0_22:5	C45H80NO8P	793.5615	10.81	ESI(+)	1169096	1285620	1376039	1276918	1.20	50.51
PE 18:0_22:6	C45H78NO8P	791.5459	9.9	ESI(-)	3560836	3181198	2966390	3236141	2.84	119.14
PE 18:1_18:2	C41H76NO8P	741.5306	9.09	ESI(+)	736752	785126	743866	755248	0.69	28.79
PE 18:1_20:4	C43H76NO8P	765.5296	8.38	ESI(+)	1369267	1281135	1283234	1311212	1.24	51.94
PE 18:1_22:6	C45H76NO8P	789.5369	7.62	ESI(-)	2113691	2111000	2189416	2138036	1.95	81.70
PE 18:1_O-16:1	C39H76NO7P	701.5354	11.32	ESI(-)	3370200	3304072	3306324	3326865	2.91	122.23
PE 18:2_18:1	C41H76NO8P	741.5301	8.7	ESI(+)	1234985	1309762	1299767	1281505	1.21	50.70
PE 18:2_18:2	C41H74NO8P	739.5146	8.14	ESI(-)	2703517	2785064	2717030	2735204	2.43	102.06
PE 19:1_24:0	C48H94NO8P	843.6707	12.36	ESI(+)	1604927	1546498	1573500	1574975	1.50	62.92
PE 22:6_16:0	C43H74NO8P	763.5145	7.91	ESI(+)	1207741	1258193	1372606	1279513	1.21	50.62
PE 22:6_18:0	C45H78NO8P	791.5444	10.54	ESI(+)	734304	769620	761462	755129	0.69	28.78
PE 22:6_18:1	C45H76NO8P	789.5329	7.93	ESI(+)	2331944	2314823	2270933	2305900	2.22	93.36
PE 23:0_13:1	C41H80NO8P	745.5629	11.69	ESI(-)	2349227	3447014	3010679	2935640	2.59	108.89
PE 36:4	C41H74NO8P	739.5124	10.25	ESI(+)	1724075	1649469	1626760	1666768	1.59	66.74
PE 38:5	C43H76NO8P	765.5267	10.99	ESI(+)	1523394	1556898	1483866	1521386	1.44	60.69
PE 38:8	C43H70NO8P	759.4852	7.09	ESI(+)	1007175	971386	1049070	1009210	0.94	39.36
PE 40:3	C45H84NO8P	797.597	11.89	ESI(+)	2692036	2654896	2560987	2635973	2.55	107.10
PE 40:6	C45H78NO8P	791.5469	10.19	ESI(+)	1091310	1095658	1127291	1104753	1.03	43.34
PE 40:7	C45H76NO8P	789.5278	10.47	ESI(-)	1579004	1492150	1103798	1391651	1.34	56.26
PE 40:9	C45H72NO8P	785.4967	7.76	ESI(+)	1534570	1814281	1559406	1636086	1.56	65.47
PE 42:7	C47H80NO8P	817.56	11.63	ESI(+)	2079138	2147626	2083703	2103489	2.02	84.93
PE 42:8	C47H78NO8P	815.5441	10.83	ESI(+)	970634	896994	978647	948758	0.88	36.85
PE 42:9	C47H76NO8P	813.5283	9.96	ESI(+)	2456007	2377367	2410924	2414766	2.33	97.89
PE O-16:0	C21H44NO7P	453.2851	2.61	ESI(+)	526647	510701	539623	525657	0.46	19.23
PE O-16:1/16:1	C37H72NO7P	673.5021	9.09	ESI(+)	1039344	1097518	1041093	1059318	0.99	41.45
PE O-16:1_18:1	C39H76NO7P	701.5365	11.54	ESI(-)	1992243	1966605	2001328	1986725	1.82	76.55
PE O-16:1_18:2	C39H74NO7P	699.5202	9.52	ESI(-)	808527	150799	781900	580409	0.68	28.61
PE O-16:1_20:1	C41H80NO7P	729.5672	11.92	ESI(+)	1913111	1916202	2068358	1965890	1.89	79.20
PE O-16:1_20:4	C41H74NO7P	723.5197	9.17	ESI(-)	8680910	8555580	8439876	8558789	7.16	300.58
PE O-16:1_20:5	C41H72NO7P	721.5031	7.91	ESI(+)	1140649	1093650	1000977	1078425	1.01	42.24
PE O-16:1_22:4	C43H78NO7P	751.551	11.09	ESI(-)	2332599	2695474	2635666	2554580	2.28	95.90
PE O-16:1_22:5	C43H76NO7P	749.5353	9.41	ESI(-)	3858531	3850945	3863779	3857752	3.34	140.33
PE O-16:1_24:4	C45H82NO7P	779.5851	11.81	ESI(-)	2529172	2744139	2538547	2603953	2.32	97.59
PE O-18:0	C23H48NO7P	481.3167	3.49	ESI(+)	977415	1007806	969270	984830	0.91	38.35
PE O-18:0_20:4	C43H80NO7P	753.5645	11.42	ESI(-)	1297041	1305039	1240597	1280892	1.25	52.49

PE O-18:0_22:4	C45H84NO7P	781.5978	11.84	ESI(-)	2226236	2214918	2145034	2195396	1.99	83.66
PE O-18:0_22:6	C45H80NO7P	777.5668	11.51	ESI(-)	1941650	2093066	1988781	2007832	1.84	77.27
PE O-18:1/18:1	C41H80NO7P	729.5678	11.71	ESI(+)	1577475	1631831	1638198	1615835	1.54	64.62
PE O-18:1_18:2	C41H78NO7P	727.5517	11.45	ESI(-)	2777863	2667900	2694886	2713550	2.41	101.32
PE O-18:1_20:3	C43H80NO7P	753.569	11.42	ESI(-)	2776330	2779762	2777069	2777720	2.46	103.51
PE O-18:1_20:4	C43H78NO7P	751.551	11.09	ESI(-)	2332599	2656523	2571070	2520064	2.26	94.73
PE O-18:1_22:3	C45H84NO7P	781.6134	11.58	ESI(+)	2413742	2433804	2499061	2448869	2.36	99.31
PE O-18:1_22:4	C45H82NO7P	779.5811	11.63	ESI(+)	2096373	2155612	2189602	2147196	2.07	86.75
PE O-18:1_22:5	C45H80NO7P	777.5684	11.91	ESI(+)	1551125	1560085	1613655	1574955	1.50	62.92
PE O-18:2_18:1| PE P-18:1_18:1	C41H78NO7P	727.5558	11.85	ESI(-)	1650499	1538434	1492137	1560357	1.48	62.01
PE O-18:2/18:2	C41H76O7PN	725.5339	9.551	ESI(+)	1657739	1650907	1659057	1655901	1.58	66.29
PE O-18:2_20:4| PE P-18:1_20:4	C43H76NO7P	749.5355	9.41	ESI(-)	4294737	4054737	3997466	4115647	3.55	149.12
PE O-18:2_22:5	C45H78NO7P	775.5556	9.32	ESI(-)	2372459	1578163	2694985	2215202	2.01	84.34
PE O-18:2_22:6	C45H76NO7P	773.5355	8.93	ESI(+)	821123	739273	750673	770356	0.70	29.42
PE O-18:4_22:4	C45H76NO7P	773.5337	11.15	ESI(-)	1360614	1289364	1356617	1335532	1.29	54.35
PE O-20:1_20:4	C45H82NO7P	779.5827	12.2	ESI(+)	496823	556740	513877	522480	0.45	19.10
PE O-20:1_22:5	C47H84NO7P	805.5978	11.93	ESI(+)	1641624	1578985	1617039	1612549	1.54	64.49
PE O-20:1_22:6	C47H82NO7P	803.5934	11.58	ESI(+)	2286249	2259921	2393520	2313230	2.23	93.66
PE O-20:2_18:1	C43H82NO7P	755.5838	11.92	ESI(-)	1267368	1269119	1303477	1279988	1.25	52.45
PG 14:0_16:0	C36H71O10P	694.4776	5.98	ESI(+)	678529	631827	668815	659724	3.61	151.66
PG 16:0_16:0	C38H75O10P	722.5083	7.26	ESI(+)	961475	879606	1029925	957002	5.23	219.81
PG 16:0_16:1	C38H73O10P	720.4978	6.11	ESI(+)	1952864	1948862	1999642	1967123	10.75	451.37
PG 16:0_18:1	C40H77O10P	748.5249	7.45	ESI(+)	2227221	1979893	1983102	2063405	11.27	473.44
PG 16:0_18:2	C40H75O10P	746.5094	6.43	ESI(+)	2297689	2123747	2006625	2142687	11.71	491.62
PG 16:0_20:4	C42H75O10P	770.5082	6.26	ESI(+)	1468438	1451155	1450421	1456671	7.96	334.36
PG 16:0_20:5	C42H73O10P	768.493	5.63	ESI(+)	774368	760682	781679	772243	4.23	177.46
PG 16:0_22:5	C44H77O10P	796.5244	7.11	ESI(+)	551311	584338	561091	565580	3.10	130.08
PG 16:0_22:6	C44H75O10P	794.5091	6.04	ESI(+)	959091	862651	939834	920525	5.03	211.45
PG 18:0_18:2	C42H79O10P	774.541	7.92	ESI(-)	1607632	1593619	1484310	1561854	0.77	32.26
PG 18:0_20:4	C44H79O10P	798.54	8.13	ESI(+)	821617	542547	570415	644860	3.53	148.26
PG 18:1/18:1	C42H79O10P	774.5408	7.64	ESI(-)	4313011	4239440	4215820	4256090	2.10	88.23
PG 18:1_18:2	C42H77O10P	772.5253	6.6	ESI(-)	341135	563256	542752	482381	0.23	9.83
PG 18:1_20:4	C44H77O10P	796.5248	6.36	ESI(+)	977814	985182	998943	987313	5.40	226.76
PG 18:1_22:6	C46H77O10P	820.5254	5.47	ESI(-)	630958	615556	612265	619593	0.30	12.68
PG 18:2_22:6	C46H75O10P	818.5097	5.07	ESI(-)	273070	247133	267018	262407	0.13	5.26
PG 20:0_18:5	C44H77O10P	796.5238	6.87	ESI(+)	770600	813940	744872	776471	4.25	178.43
PG 20:4_20:4	C46H75O10P	818.5088	4.91	ESI(+)	956769	1002133	932326	963743	5.27	221.36
PG 20:4_22:6	C48H75O10P	842.5085	4.96	ESI(-)	135277	109844	103527	116216	0.05	2.23
PG 22:5_16:0	C44H77O10P	796.522	6.03	ESI(+)	741393	701321	727215	723310	3.96	166.24
PG 22:6_18:2	C46H75O10P	818.5096	5.07	ESI(-)	259972	271654	291850	274492	0.13	5.51
PG 22:6_22:6	C50H75O10P	866.5096	4.81	ESI(+)	497954	515130	477226	496770	2.72	114.31
PI 16:0_18:2	C43H79O13P	834.5214	6.43	ESI(+)	612038	580223	611400	601220	0.43	18.22
PI 16:0_20:4	C45H79O13P	858.5248	6.05	ESI(+)	673097	652709	570500	632102	0.48	20.14
PI 16:0_22:5	C47H81O13P	884.5402	6.49	ESI(+)	394056	386128	399624	393269	0.13	5.28
PI 16:0_22:6	C47H79O13P	882.5266	5.83	ESI(+)	425648	442710	461991	443450	0.20	8.40
PI 16:1_18:2	C43H77O13P	832.5092	8.67	ESI(-)	789402	816083	791708	799064	0.26	10.72
PI 18:0_18:2	C45H83O13P	862.5566	7.82	ESI(-)	1842977	1789564	1818086	1816876	1.31	55.08

PI 18:0_18:2	C45H83O13P	862.5558	7.83	ESI(+)	1145848	1222534	1087944	1152109	1.25	52.50
PI 18:0_20:4	C47H83O13P	886.5568	7.36	ESI(+)	2472261	2586966	2393113	2484113	3.22	135.40
PI 18:0_22:5	C49H85O13P	912.5726	7.48	ESI(+)	578687	570557	568636	572627	0.39	16.44
PI 18:0_22:6	C49H83O13P	910.557	7.04	ESI(+)	368587	383215	392429	381410	0.11	4.54
PI 18:1_20:4	C47H81O13P	884.5403	6.16	ESI(+)	303864	304168	355231	321088	0.02	0.79
PI 20:4_18:1	C47H81O13P	884.5385	5.84	ESI(+)	344398	284907	337094	322133	0.02	0.85
PI 22:5_16:0	C47H81O13P	884.54	6.14	ESI(+)	559005	517692	549966	542221	0.35	14.55
PI 34:1	C43H81O13P	836.5392	7.24	ESI(+)	2495967	2572909	2710161	2593012	3.39	142.17
PI 34:3	C43H77O13P	832.5086	9.31	ESI(+)	1127131	984585	933339	1015018	1.05	43.97
PI 36:3	C45H81O13P	860.5387	6.06	ESI(+)	343143	378987	301583	341238	0.05	2.04
PI 38:3	C47H85O13P	888.565	7.41	ESI(-)	1605239	1607580	1678815	1630545	1.12	46.96
PI 38:3	C47H85O13P	888.5716	7.36	ESI(+)	886254	902403	891437	893365	0.87	36.40
PS 16:0_18:1	C40H76NO10P	761.5201	7.39	ESI(-)	819326	931678	771218	840741	0.72	30.34
PS 16:0_20:4	C45H76NO10P	821.5189	8.12	ESI(-)	1359240	1386522	1384666	1376809	1.26	53.06
PS 16:1_18:0	C40H76NO10P	761.5196	7.39	ESI(-)	858937	931678	880040	890218	0.77	32.44
PS 18:0_18:1	C42H80NO10P	789.5517	9.29	ESI(-)	2101364	2251380	2257854	2203533	2.10	88.10
PS 18:0_18:2	C42H78NO10P	787.5362	7.84	ESI(-)	1693845	1738796	1717111	1716584	1.61	67.46
PS 18:0_20:3	C44H80NO10P	813.5517	8.37	ESI(-)	1905720	1897635	1841840	1881732	1.77	74.46
PS 18:0_20:4	C44H78NO10P	811.5362	7.64	ESI(-)	2186395	2191726	2138212	2172111	2.07	86.77
PS 18:0_22:4	C46H82NO10P	839.5671	9.04	ESI(-)	1864779	1838451	1884483	1862571	1.75	73.65
PS 18:0_22:5	C46H80NO10P	837.5515	7.82	ESI(-)	1423620	1408837	2011374	1614610	1.50	63.14
PS 18:0_22:6	C46H78NO10P	835.5362	7.32	ESI(-)	2169493	2137956	2196509	2167986	2.06	86.59
PS 18:1_18:1	C42H78NO10P	787.5375	8.12	ESI(-)	636470	700004	703116	679863	0.56	23.52
PS 18:2_20:1	C44H80NO10P	813.5516	8.37	ESI(-)	1835080	1818619	1776804	1810168	1.70	71.43
PS 20:3_20:4	C46H76NO10P	833.5181	7.65	ESI(-)	1645679	1614610	1632996	1631095	1.52	63.84
PS 20:3_22:6	C48H76NO10P	857.5184	7.32	ESI(-)	1107922	1126473	1126370	1120255	1.00	42.19
PS 20:4_18:0	C44H78NO10P	811.5362	7.87	ESI(-)	821259	850710	834603	835524	0.72	30.12
PS 21:2_18:1	C45H82NO10P	827.5659	11.67	ESI(-)	1392529	1417920	1366758	1392402	1.28	53.72
PS 21:2_20:4	C47H80NO10P	849.549	10.47	ESI(-)	1139726	1123641	1174649	1146005	1.03	43.28
PS 22:6_18:0	C46H78NO10P	835.5351	7.61	ESI(-)	1623889	1605711	1501036	1576879	1.47	61.54
PS 22:6_18:1	C46H76NO10P	833.5183	8.01	ESI(-)	262510	279942	262673	268375	0.14	6.08
PS 22:7_18:0	C46H76NO10P	833.5217	7.45	ESI(-)	206578	222532	251116	226742	0.10	4.32
PS 36:1	C42H80NO10P	789.5547	8.26	ESI(-)	417602	365711	422264	401859	0.28	11.74
PS 38:2	C44H82NO10P	815.5651	9.34	ESI(-)	6645854	6615522	6507585	6589654	6.52	273.99
PS 38:6	C44H74NO10P	807.5051	6.04	ESI(-)	447661	449828	449018	448836	0.33	13.73
PS 40:2	C46H86NO10P	843.5977	11.51	ESI(-)	2233404	2116430	2133255	2161030	2.05	86.30
PS 40:4	C46H82NO10P	839.5651	8.05	ESI(-)	2221715	2212185	2138402	2190767	2.08	87.56
PS 40:6	C46H78NO10P	835.5347	8.39	ESI(-)	255057	255978	947977	486337	0.36	15.32
PS 42:6	C48H82NO10P	863.5629	9.62	ESI(-)	370280	342372	372763	361805	0.24	10.04
PS 42:9	C48H76NO10P	857.5173	9.39	ESI(-)	875662	834709	852715	854362	0.74	30.92
SM 18:0;2O/16:0	C39H81N2O6P	704.583	8.11	ESI(+)	9443755	9197614	9195708	9279025.7	1.45	61.06
SM 18:0;2O/18:0	C41H85N2O6P	732.6147	10.53	ESI(+)	1823025	1759873	1475539	1686145.7	0.32	13.37
SM 18:0;2O/20:0	C43H89N2O6P	760.6503	11.8	ESI(+)	2949424	2889891	2924437	2921250.7	0.50	21.12
SM 18:0;2O/22:0	C45H93N2O6P	788.6794	11.68	ESI(+)	14838293	15053424	14689898	14860538	2.29	96.12
SM 18:0;2O/24:0	C47H97N2O6P	816.7105	12.04	ESI(+)	3523523	2087243	3360064	2990276.7	0.51	21.56
SM 18:0;2O/24:1	C47H95N2O6P	814.6927	12.36	ESI(+)	11759767	11725626	11619207	11701533	1.82	76.27
SM 18:0;O2/22:3	C45H87N2O6P	782.6371	11.91	ESI(+)	6929647	6515766	6712076	6719163	1.07	44.98

SM 18:1;2O/14:0	C37H75N2O6P	674.5354	6	ESI(+)	177070	466013	494721	379268	0.12	5.16
SM 18:1;2O/15:0	C38H77N2O6P	688.5515	6.62	ESI(+)	1947639	514947	1756750	1406445.3	0.28	11.61
SM 18:1;2O/16:0	C39H79N2O6P	702.5678	7.4	ESI(+)	80199550	76770306	70525807	75831888	11.41	479.10
SM 18:1;2O/17:0	C40H81N2O6P	716.5798	8.32	ESI(+)	1124601	2993308	1014643	1710850.7	0.32	13.52
SM 18:1;2O/18:0	C41H83N2O6P	730.5985	9.46	ESI(+)	11886570	11560423	11781463	11742819	1.82	76.53
SM 18:1;2O/20:0	C43H87N2O6P	758.6301	11.58	ESI(+)	7306070	9361945	9198066	8622027	1.36	56.93
SM 18:1;2O/21:0	C44H89N2O6P	772.6454	11.85	ESI(+)	1168841	1530960	1259109	1319636.7	0.26	11.06
SM 18:1;2O/22:0	C45H91N2O6P	786.6616	12.03	ESI(+)	12610163	12396655	12120137	12375652	1.92	80.51
SM 18:1;2O/23:0	C46H93N2O6P	800.6758	11.88	ESI(+)	3500255	3627139	3753201	3626865	0.61	25.56
SM 18:1;2O/24:0	C47H95N2O6P	814.6911	12.83	ESI(+)	1639598	1662709	1603356	1635221	0.31	13.05
SM 18:1;2O/24:1	C47H93N2O6P	812.6777	12.01	ESI(+)	41068648	38165193	40015655	39749832	6.01	252.45
SM 18:1;2O/24:2	C47H91N2O6P	810.6619	11.68	ESI(+)	19795228	19448658	19075756	19439881	2.97	124.88
SM 18:1;2O/25:0	C48H97N2O6P	828.7088	12.76	ESI(+)	2741953	1780380	2291293	2271208.7	0.41	17.04
SM 18:1;2O/26:1	C49H97N2O6P	840.7094	12.02	ESI(+)	4425126	1031818	2803124	2753356	0.48	20.07
SM 18:1;O2/19:0	C42H85O6PN2	744.6152	10.45	ESI(+)	969009	950197	917546	959603	0.21	8.80
SM 18:2;2O/16:0	C39H77N2O6P	700.5507	6.21	ESI(+)	3642694	3132696	3032074	3269154.7	0.55	23.31
SM 18:2;2O/18:0	C41H81N2O6P	728.5826	7.71	ESI(+)	1456963	1450389	1156401	1354584.3	0.27	11.28
SM 18:2;2O/20:0	C43H85N2O6P	756.6139	9.93	ESI(+)	1815625	1459701	1063579	1446301.7	0.28	11.86
SM 18:2;2O/22:0	C45H89N2O6P	784.6461	11.68	ESI(+)	6668024	6611808	6562695	6614175.7	1.06	44.32
SM 18:2;2O/22:1	C45H87N2O6P	782.6331	10.09	ESI(+)	724230	755033	693427	724230	0.17	7.32
SM 18:2;2O/23:0	C46H91N2O6P	798.6666	12.15	ESI(+)	2231608	2366327	2264167	2287367.3	0.41	17.14
SM 18:2;2O/24:0	C47H93N2O6P	812.6736	11.68	ESI(+)	4365466	4435482	4297766	4366238	0.72	30.20
SM 18:2;2O/25:0	C48H95N2O6P	826.694	12	ESI(+)	2823493	7863740	8006612	6231281.7	1.00	41.91
SM 19:1;2O/22:0	C46H93N2O6P	800.6771	12.19	ESI(+)	7006742	6896212	6958297	6953750.3	1.11	46.45
SM 24:1;2O/17:1	C46H91N2O6P	798.6616	11.91	ESI(+)	6033206	6091479	6005292	6043325.7	0.97	40.73
SM 41:0;2O	C47H89N2O6P	808.6455	10.79	ESI(+)	236763	128952	612980	326231.67	0.11	4.82
SM 42:4;2O	C49H91N2O6P	834.6596	12.01	ESI(+)	10618184	9658252	11755215	10677217	1.66	69.84
SM 44:5;O2	C56H115N2O6P	942.8486	12.39	ESI(+)	2224244	2133681	2194721	2184215.3	0.39	16.49
SM 51:0;2O	C46H95N2O6P	802.6924	12.04	ESI(+)	7754362	4013035	7386953	6384783.3	1.02	42.88
SPB 18:0;O2	C18H39NO2	301.2965	1.85	ESI(+)	2330018	2303164	2482386	2371856	0.36	15.11
SPB 18:1;O2	C18H37NO2	299.2826	2.1	ESI(+)	648991	661326	620636	643651	0.15	6.09
TG 10:0_14:0_16:0	C43H82O6	939.9278	13.05	ESI(+)	1916431	1911300	1902723	1910151	0.49	20.58
TG 10:0_14:0_18:0	C45H86O6	722.64	13.37	ESI(+)	1874803	992895	765785	1211161	0.31	13.14
TG 10:0_16:0_16:0	C45H86O6	722.6398	13.36	ESI(+)	5317794	5414167	5382074	5371345	1.37	57.41
TG 10:0_18:0_8:0	C39H74O6	638.5563	11.98	ESI(+)	7166389	7198031	7053178	7139199	1.81	76.22
TG 10:0_8:0_8:0	C29H54O6	498.3923	5.96	ESI(+)	8203184	8074247	8126759	8134730	2.07	86.81
TG 12:0/12:0/12:0	C39H74O6	638.5573	12.29	ESI(+)	5219887	5158725	5094976	5157863	1.31	55.14
TG 12:0_10:0_14:0	C39H74O6	638.5579	11.56	ESI(+)	8776765	8486383	8740424	8667857	2.2	92.49
TG 12:0_12:0_18:0	C45H86O6	722.64	13.37	ESI(+)	790093	1421252	765785	992377	0.26	10.82
TG 12:0_14:0_16:0	C45H86O6	722.6405	13.36	ESI(+)	3785151	3622390	3712784	3706775	0.95	39.7
TG 12:0_14:0_6:0	C35H66O6	582.4834	11.68	ESI(+)	9139870	9060198	9111904	9103991	2.31	97.13
TG 12:0_18:2_16:0	C49H90O6	774.6725	13.45	ESI(+)	3829405	3775280	3761152	3788612	0.97	40.57
TG 14:0/14:0/14:0	C45H86O6	722.6401	13.36	ESI(+)	2715019	2647260	2629507	2663929	0.68	28.6
TG 14:0_12:0_18:1	C47H88O6	748.6565	13.38	ESI(+)	2827286	2679410	2795870	2767522	0.71	29.7
TG 14:0_14:0_16:1	C47H88O6	748.6565	13.06	ESI(+)	2014494	2137519	2031808	2061274	0.53	22.19
TG 14:0_14:0_18:0	C49H94O6	778.7045	14.4	ESI(+)	3942765	4609771	3782877	4111804	1.05	44.01
TG 14:0_16:0_18:1	C51H96O6	804.7199	14.36	ESI(+)	5405978	5376474	5398700	5393717	1.37	57.65

TG 14:0_16:0_18:2	C51H94O6	802.7041	13.84	ESI(+)	4812791	4766365	4795354	4791503	1.22	51.24
TG 14:0_18:1_20:1	C55H102O6	1085.943	15.21	ESI(+)	603902	552589	578345	578279	0.15	6.41
TG 14:0_18:2_18:2	C53H94O6	826.7046	13.39	ESI(+)	3653420	3675536	3643458	3657471	0.93	39.17
TG 14:0_22:5_18:0	C57H100O6	880.7504	13.86	ESI(+)	199429	397254	2055899	884194	0.23	9.66
TG 16:0/16:0/16:0	C51H98O6	806.736	15.06	ESI(+)	5192943	5208234	5181545	5194241	1.32	55.52
TG 16:0_10:0_10:0	C39H74O6	638.5592	11.4	ESI(+)	6014261	5823658	5920399	5919439	1.51	63.24
TG 16:0_15:0_18:1	C52H98O6	818.7359	14.4	ESI(+)	4423283	4302680	4464438	4396800	1.12	47.04
TG 16:0_16:0_17:1	C52H98O6	818.7353	14.69	ESI(+)	2677960	2736784	2811959	2742234	0.7	29.43
TG 16:0_16:0_18:0	C53H102O6	1061.946	15.85	ESI(+)	1849352	1969595	1939878	1919608	0.49	20.68
TG 16:0_16:0_18:1	C53H100O6	832.7506	14.71	ESI(+)	1827866	1865302	1788876	1827348	0.47	19.7
TG 16:0_16:0_18:2	C53H98O6	830.7357	14.39	ESI(+)	6459931	6479092	6377465	6438829	1.64	68.77
TG 16:0_16:0_18:3	C53H96O6	828.7211	13.83	ESI(+)	5722302	5710404	5683761	5705489	1.45	60.96
TG 16:0_16:0_20:1	C55H104O6	860.7829	15.81	ESI(+)	2250638	2354474	2328233	2311115	0.59	24.85
TG 16:0_16:0_20:3	C55H100O6	856.7511	14.36	ESI(+)	3319659	3121807	3378712	3273393	0.84	35.09
TG 16:0_16:0_4:0	C39H74O6	638.5583	12.64	ESI(+)	3803655	3874839	3825641	3834712	0.98	41.06
TG 16:0_16:1_17:1	C52H96O6	816.7195	14.14	ESI(+)	2803208	2816892	2824192	2814764	0.72	30.21
TG 16:0_16:1_18:0	C53H100O6	832.7541	14.57	ESI(+)	1602219	1591255	1703088	1632187	0.42	17.62
TG 16:0_16:1_18:2	C53H96O6	828.7188	13.67	ESI(+)	1037016	1082995	1020194	1046735	0.27	11.39
TG 16:0_16:1_20:3	C55H98O6	854.7351	14.31	ESI(+)	1372371	1315095	1557283	1414916	0.36	15.31
TG 16:0_17:0_16:1	C52H98O6	818.7364	13.86	ESI(+)	3727771	3569971	3636136	3644626	0.93	39.04
TG 16:0_18:0_16:1	C53H100O6	832.7558	14.38	ESI(+)	3108246	3094306	3074415	3092322	0.79	33.16
TG 16:0_18:0_18:1	C55H104O6	860.7818	14.97	ESI(+)	3122706	3118865	3041356	3094309	0.79	33.18
TG 16:0_18:0_18:2	C55H102O6	1085.947	15.03	ESI(+)	671144	583860	654089	636364	0.17	7.03
TG 16:0_18:1_16:0	C53H100O6	832.751	15.01	ESI(+)	5194300	5135624	5145102	5158342	1.31	55.14
TG 16:0_18:1_16:1	C53H98O6	830.7339	14.11	ESI(+)	1996563	2048507	2026298	2023789	0.52	21.79
TG 16:0_18:1_18:0	C55H104O6	860.7829	15.29	ESI(+)	1302191	1357159	1398627	1352659	0.35	14.65
TG 16:0_18:1_18:2	C55H100O6	856.7509	14.38	ESI(+)	5697966	5612407	5760350	5690241	1.45	60.8
TG 16:0_18:1_22:5	C59H102O6	1133.954	14.28	ESI(+)	3082253	2926925	2898009	2969062	0.76	31.85
TG 16:0_18:1_22:6	C59H100O6	904.7503	14.35	ESI(+)	1281476	1275468	1313874	1290273	0.33	13.99
TG 16:0_18:2_14:0	C51H94O6	802.7024	14.41	ESI(+)	1377701	1379689	1424550	1393980	0.36	15.09
TG 16:0_18:2_16:0	C53H98O6	830.7386	13.83	ESI(+)	2492732	2524218	2507096	2508015	0.64	26.94
TG 16:0_18:2_17:0	C54H100O6	844.7524	14.36	ESI(+)	3034827	3122736	3049329	3068964	0.78	32.91
TG 16:0_18:2_22:4	C59H102O6	1133.953	14.57	ESI(+)	1320517	1321304	1262897	1301573	0.34	14.11
TG 16:0_18:2_22:5	C59H100O6	904.7519	13.88	ESI(+)	1077179	1161867	1139533	1126193	0.29	12.24
TG 16:0_18:2_22:6	C59H98O6	902.7331	13.85	ESI(+)	2213227	2231460	2187473	2210720	0.57	23.78
TG 16:0_18:3_16:0	C53H96O6	828.721	13.39	ESI(+)	1253383	1262461	1229960	1248601	0.32	13.54
TG 16:0_18:4_18:1	C55H96O6	852.7207	13.41	ESI(+)	7669567	7681259	7723570	7691465	1.95	82.1
TG 16:0_20:2_20:2	C59H106O6	910.7992	15.81	ESI(+)	636873	797235	817039	750382	0.2	8.24
TG 16:0_20:3_16:1	C55H98O6	854.7374	13.23	ESI(+)	3787932	3663928	3722482	3724781	0.95	39.89
TG 16:0_20:3_20:3	C59H102O6	1133.949	14.21	ESI(+)	952976	990767	905621	949788	0.25	10.36
TG 16:0_20:3_22:6	C61H100O6	928.7555	13.41	ESI(+)	5307423	5499746	5389723	5398964	1.37	57.7
TG 16:0_20:4_22:5	C61H100O6	928.7541	12.86	ESI(+)	8374853	8558471	7990284	8307869	2.11	88.66
TG 16:0_22:2_18:2	C59H106O6	910.7983	14.66	ESI(+)	1033829	1053427	1035747	1041001	0.27	11.33
TG 16:0_4:0_16:0	C39H74O6	638.5592	11.26	ESI(+)	4275026	3931356	4191782	4132721	1.05	44.23
TG 16:0_4:0_18:1	C41H76O6	664.5712	12.08	ESI(+)	4326352	4406404	4155465	4296074	1.09	45.97
TG 16:0_8:0_12:0	C39H74O6	638.5568	11.98	ESI(+)	12650521	12457384	12536124	12548010	3.19	133.77
TG 16:1_16:0_16:1	C51H94O6	802.7045	13.58	ESI(+)	1680160	1587418	1530589	1599389	0.41	17.27

TG 16:1_16:0_18:2	C53H96O6	828.7188	14.41	ESI(+)	1190374	1327902	1294843	1271040	0.33	13.78
TG 16:1_16:0_18:3	C53H94O6	826.7051	12.88	ESI(+)	5929559	6086541	5821934	5946011	1.51	63.52
TG 16:1_16:1_18:1	C53H96O6	828.7195	13.73	ESI(+)	1549971	1410928	1534372	1498424	0.39	16.2
TG 16:1_18:1_14:0	C51H94O6	802.7053	13.38	ESI(+)	1539790	1625778	1460464	1542011	0.4	16.66
TG 16:1_18:1_17:1	C54H98O6	842.7364	13.83	ESI(+)	2714060	2798948	2716514	2743174	0.7	29.44
TG 16:1_18:1_18:3	C55H96O6	852.7183	14.39	ESI(+)	3661131	3669760	3690574	3673822	0.94	39.35
TG 16:1_18:2_18:2	C55H96O6	852.722	12.8	ESI(+)	4014381	3744774	3915246	3891467	0.99	41.66
TG 16:1_18:3_18:2	C55H94O6	850.7039	12.74	ESI(+)	4513335	4706414	4509735	4576495	1.17	48.95
TG 16:1_18:4_18:1	C55H94O6	850.7047	13.02	ESI(+)	2116871	2067185	2165486	2116514	0.54	22.78
TG 17:1/17:1/17:1	C54H98O6	842.7354	14.36	ESI(+)	1347373	1418389	1360409	1375390	0.35	14.89
TG 17:1_16:1_18:1	C54H98O6	842.7361	14.05	ESI(+)	1391096	1360438	1497698	1416411	0.36	15.33
TG 17:1_18:0_18:2	C56H102O6	1097.945	14.18	ESI(+)	2324283	2424573	2406151	2385002	0.61	25.63
TG 17:1_18:1_16:0	C54H100O6	844.7516	14.69	ESI(+)	2090206	2243924	2218294	2184141	0.56	23.5
TG 17:1_18:1_18:1	C56H102O6	1097.946	14.56	ESI(+)	1836423	1833854	1741465	1803914	0.46	19.45
TG 18:0_16:1_18:2	C55H100O6	856.7488	15.89	ESI(+)	967799	1054332	1027273	1016468	0.26	11.07
TG 18:0_18:0_18:2	C57H106O6	886.7975	15.77	ESI(+)	348438	324122	389803	354121	0.1	4.02
TG 18:0_18:1_16:0	C55H104O6	860.7834	15.81	ESI(+)	2082163	2135272	2140516	2119317	0.54	22.81
TG 18:0_20:4_20:4	C61H102O6	1157.945	14.16	ESI(+)	721113	698614	780039	733255	0.19	8.06
TG 18:1/18:1/18:1	C57H104O6	884.7787	14.35	ESI(+)	2013172	1962880	1951559	1975870	0.51	21.28
TG 18:1_14:0_18:0	C53H100O6	832.7533	14.14	ESI(+)	1976733	1994613	2038384	2003243	0.51	21.57
TG 18:1_16:0_18:2	C55H100O6	856.7523	12.44	ESI(+)	4182956	4379570	4109679	4224068	1.08	45.2
TG 18:1_16:0_20:1	C57H106O6	886.7981	15.75	ESI(+)	1673702	1726140	1630244	1676695	0.43	18.1
TG 18:1_16:0_20:3	C57H102O6	1109.945	14.56	ESI(+)	3664427	3606924	3651003	3640785	0.93	39
TG 18:1_16:0_4:0	C41H76O6	664.5687	11.94	ESI(+)	5126515	4767553	4970979	4955016	1.26	52.98
TG 18:1_16:1_16:0	C53H98O6	830.7362	13.38	ESI(+)	1152328	1236816	1160324	1183156	0.31	12.85
TG 18:1_16:1_17:0	C54H100O6	844.7529	13.83	ESI(+)	4058160	3976382	3951275	3995272	1.02	42.77
TG 18:1_16:1_18:1	C55H100O6	856.7521	12.73	ESI(+)	2679428	2464826	2685846	2610033	0.67	28.03
TG 18:1_16:1_19:1	C56H102O6	1097.947	14.76	ESI(+)	1014354	963673	1003387	993805	0.26	10.83
TG 18:1_17:0_16:2	C54H98O6	842.7335	13.38	ESI(+)	2363533	2420835	2379050	2387806	0.61	25.66
TG 18:1_17:1_16:0	C54H100O6	844.7505	15.04	ESI(+)	1271159	1332199	1385404	1329587	0.34	14.4
TG 18:1_17:1_18:1	C56H102O6	1097.947	13.83	ESI(+)	978801	1005183	900909	961631	0.25	10.49
TG 18:1_18:0_14:0	C53H100O6	832.7542	13.81	ESI(+)	1847879	1901590	1894385	1881285	0.48	20.27
TG 18:1_18:0_18:2	C57H104O6	884.783	14.66	ESI(+)	1202395	1166832	1108975	1159401	0.3	12.59
TG 18:1_18:0_18:3	C57H102O6	1109.943	14.56	ESI(+)	2083406	1916375	1987731	1995837	0.51	21.49
TG 18:1_18:1_14:0	C53H98O6	830.7371	13.67	ESI(+)	1870697	1794629	1855769	1840365	0.47	19.84
TG 18:1_18:1_18:3	C57H100O6	880.7508	14.36	ESI(+)	1171118	1196106	1213697	1193640	0.31	12.96
TG 18:1_18:1_20:2	C59H106O6	910.7984	15.16	ESI(+)	722993	695611	736791	718465	0.19	7.9
TG 18:1_18:1_20:3	C59H104O6	908.7831	14.53	ESI(+)	1162632	1164852	1152576	1160020	0.3	12.6
TG 18:1_18:2_16:0	C55H100O6	856.7558	12.23	ESI(+)	2750491	3058345	2879750	2896195	0.74	31.07
TG 18:1_18:2_18:0	C57H104O6	884.784	14.08	ESI(+)	967407	1010981	1012523	996970	0.26	10.86
TG 18:1_18:2_18:3	C57H98O6	878.7352	13.44	ESI(+)	1967263	1913135	1918676	1933025	0.5	20.82
TG 18:1_18:4_18:1	C57H98O6	878.7357	13.86	ESI(+)	1074495	1095317	1216366	1128726	0.29	12.27
TG 18:1_20:2_18:1	C59H106O6	910.8001	14.3	ESI(+)	1071015	1058098	988685	1039266	0.27	11.31
TG 18:1_20:3_16:0	C57H102O6	1109.945	14.18	ESI(+)	4243106	4142999	3946073	4110726	1.05	44
TG 18:1_20:3_18:0	C59H106O6	910.7989	15.22	ESI(+)	845385	905306	805718	852136	0.22	9.32
TG 18:1_20:4_20:3	C61H102O6	1157.947	13.66	ESI(+)	671586	605700	714077	663788	0.17	7.32
TG 18:1_20:4_20:4	C61H100O6	928.755	12.48	ESI(+)	13673247	13658563	13566939	13632916	3.46	145.32

TG 18:1_22:2_16:0	C59H108O6	912.8145	15.76	ESI(+)	718680	671810	691303	693931	0.18	7.64
TG 18:1_4:0_16:0	C41H76O6	664.5765	11.39	ESI(+)	5079193	5152816	5028619	5086876	1.29	54.38
TG 18:2/18:2/18:2	C57H98O6	878.7372	13.03	ESI(+)	9785206	9529631	9745258	9686698	2.46	103.33
TG 18:2_16:0_16:0	C53H98O6	830.7347	12.8	ESI(+)	4996229	5064010	4911996	4990745	1.27	53.36
TG 18:2_16:0_16:1	C53H96O6	828.72	13.84	ESI(+)	2477591	2453972	2522484	2484682	0.64	26.69
TG 18:2_16:0_17:1	C54H98O6	842.7357	13.06	ESI(+)	2604622	2711917	2634758	2650432	0.68	28.46
TG 18:2_16:0_18:3	C55H96O6	852.7204	12.41	ESI(+)	4495128	4377252	4167131	4346504	1.11	46.5
TG 18:2_16:0_18:4	C55H94O6	850.7046	12.59	ESI(+)	2924383	2871627	3009508	2935173	0.75	31.49
TG 18:2_16:1_18:2	C55H96O6	852.7179	12.21	ESI(+)	3228671	3596918	3458592	3428060	0.87	36.73
TG 18:2_17:0_18:1	C56H102O6	1097.946	15.79	ESI(+)	713548	705568	718242	712453	0.19	7.84
TG 18:2_17:1_18:0	C56H102O6	1097.943	13.21	ESI(+)	534503	590561	556965	560676	0.15	6.22
TG 18:2_18:0_14:0	C53H98O6	830.7399	12.54	ESI(+)	2656559	2697859	2484826	2613081	0.67	28.06
TG 18:2_18:0_18:3	C57H100O6	880.7541	12.93	ESI(+)	3093661	3078951	2852521	3008378	0.77	32.27
TG 18:2_18:1_15:0	C54H98O6	842.7339	15.34	ESI(+)	780030	746016	759031	761692	0.2	8.36
TG 18:2_18:1_16:2	C55H96O6	852.7198	12.01	ESI(+)	7001578	6804546	6951159	6919094	1.76	73.88
TG 18:2_18:1_16:3	C55H94O6	850.7043	12.05	ESI(+)	6067868	6254700	6076387	6132985	1.56	65.51
TG 18:2_18:2_18:1	C57H100O6	880.7497	14.98	ESI(+)	2003994	2030504	2009910	2014803	0.52	21.69
TG 18:2_18:3_16:1	C55H94O6	850.7033	11.98	ESI(+)	6011358	5982797	5896981	5963712	1.52	63.71
TG 18:2_18:3_18:1	C57H98O6	878.7343	14.36	ESI(+)	2396546	2526145	2534579	2485757	0.64	26.71
TG 18:2_18:4_16:0	C55H94O6	850.7062	11.72	ESI(+)	4380482	4100682	4323076	4268080	1.09	45.67
TG 18:2_20:1_18:2	C59H104O6	908.7839	14.12	ESI(+)	2301678	2235959	2235536	2257724	0.58	24.28
TG 18:2_20:2_16:0	C57H102O6	1109.944	14.2	ESI(+)	2530297	3784497	2039354	2784716	0.71	29.89
TG 18:2_20:5_18:1	C59H98O6	902.7355	13.43	ESI(+)	3098585	3247751	3267757	3204698	0.82	34.36
TG 18:2_22:5_16:1	C59H98O6	902.738	13.56	ESI(+)	1395194	1420467	1472856	1429506	0.37	15.47
TG 18:2_22:5_18:2	C61H100O6	928.7553	12.03	ESI(+)	5044540	4984215	5025928	5018228	1.28	53.65
TG 18:3_16:0_18:2	C55H96O6	852.7207	11.73	ESI(+)	4749726	4492002	4692148	4644625	1.18	49.68
TG 18:3_18:1_18:3	C57H96O7	892.7173	13.41	ESI(+)	4763442	4835534	4913446	4837474	1.23	51.73
TG 20:2_16:0_20:2	C59H106O6	910.7997	13.84	ESI(+)	1986358	2041286	2023436	2017027	0.52	21.72
TG 20:2_18:2_18:0	C59H106O6	910.7972	13.54	ESI(+)	2100982	2057337	2143850	2100723	0.54	22.61
TG 20:3_18:2_20:3	C61H102O6	1157.945	14.56	ESI(+)	647148	655928	572440	625172	0.16	6.91
TG 20:4_16:0_18:1	C57H100O6	880.7521	14.36	ESI(+)	1541391	1664912	1614920	1607074	0.41	17.36
TG 20:4_16:1_16:1	C55H94O6	850.7032	14.14	ESI(+)	914827	956777	959452	943685	0.25	10.3
TG 20:4_18:0_18:1	C59H104O6	908.7823	15.04	ESI(+)	1841068	1714856	1945589	1833838	0.47	19.77
TG 20:4_18:1_18:0	C59H104O6	908.783	14.34	ESI(+)	3961206	3993401	3942696	3965768	1.01	42.45
TG 20:4_18:2_18:2	C59H98O6	902.7355	14.33	ESI(+)	1011928	1040320	1001969	1018072	0.26	11.09
TG 22:5_16:0_20:4	C61H100O6	928.7537	11.89	ESI(+)	3928357	4166316	3887905	3994193	1.02	42.76
TG 22:5_18:0_16:0	C59H104O6	908.7827	14.66	ESI(+)	1096036	1052906	1020716	1056553	0.27	11.5
TG 22:5_18:1_18:1	C61H104O6	932.7835	14.35	ESI(+)	1995813	2111563	1858940	1988772	0.51	21.42
TG 22:6_16:1_16:0	C57H96O6	876.7183	13.86	ESI(+)	1204349	1204506	1186317	1198391	0.31	13.01
TG 22:6_16:1_18:1	C59H98O6	902.7384	12.86	ESI(+)	5636345	5661243	5429757	5575782	1.42	59.58
TG 22:6_18:1_18:1	C61H102O6	1157.946	13.46	ESI(+)	555843	555389	547484	552905	0.15	6.14
TG 22:6_18:2_16:0	C59H98O6	902.7348	14.99	ESI(+)	962048	1038283	1011576	1003969	0.26	10.94
TG 22:6_18:2_18:0	C61H102O6	1157.947	12.96	ESI(+)	573081	562069	592593	575914	0.15	6.38
TG 22:6_18:2_18:1	C61H100O6	928.7514	13.89	ESI(+)	2217816	2171578	2123713	2171036	0.56	23.36
TG 4:0_18:0_16:1	C41H76O6	664.5734	12.43	ESI(+)	3181533	3036449	3168658	3128880	0.8	33.55
TG 44:0*	C47H90O6	750.673	13.72	ESI(+)	1391060	1349522	1449431	1396671	0.36	15.12
TG 44:0*	C47H90O6	750.6731	13.83	ESI(+)	2679949	2703620	2866835	2750135	0.7	29.52

TG 48:0*	C51H98O6	806.7362	14.91	ESI(+)	2258930	2211352	2218163	2229482	0.57	23.98
TG 50:2*	C53H98O6	830.7361	15.09	ESI(+)	1401737	1362073	1381224	1381678	0.36	14.96
TG 50:3*	C53H96O6	828.7202	13.83	ESI(+)	3257028	3284452	3347686	3296389	0.84	35.33
TG 50:4*	C53H94O6	826.7048	13.39	ESI(+)	2714206	2711077	2673963	2699749	0.69	28.98
TG 50:4*	C53H94O6	826.7035	14.36	ESI(+)	3313557	3269724	3358701	3313994	0.85	35.52
TG 51:3*	C54H98O6	842.7341	15.08	ESI(+)	1062522	1068346	1080832	1070567	0.28	11.65
TG 52:3*	C55H100O6	856.7517	13.04	ESI(+)	3366437	3238027	3489483	3364649	0.86	36.06
TG 52:3*	C55H100O6	856.7471	13.86	ESI(+)	2009664	1983657	1967577	1986966	0.51	21.4
TG 52:3*	C55H100O6	856.7507	15.01	ESI(+)	1033769	1085792	995077	1038213	0.27	11.3
TG 52:4*	C55H98O6	854.7354	13.86	ESI(+)	1444674	1186266	1321212	1317384	0.34	14.27
TG 52:4*	C55H98O6	854.7356	13.86	ESI(+)	1444674	2643074	2798665	2295471	0.59	24.68
TG 52:4*	C55H98O6	854.736	13.86	ESI(+)	4612520	4492320	4740259	4615033	1.18	49.36
TG 52:4*	C55H98O6	854.7342	15.01	ESI(+)	2251262	2207271	2215517	2224683	0.57	23.93
TG 52:5*	C55H96O6	852.7208	13.39	ESI(+)	5126852	5009655	3820759	4652422	1.18	49.76
TG 52:5*	C55H96O6	852.7191	13.81	ESI(+)	1860068	1764204	1817291	1813854	0.47	19.56
TG 53:3*	C56H102O6	1097.948	15.56	ESI(+)	1272239	1246053	1290748	1269680	0.33	13.77
TG 54:3*	C57H104O6	884.7822	14.95	ESI(+)	3577209	3684071	3642304	3634528	0.93	38.93
TG 54:4*	C57H102O6	1109.941	14.2	ESI(+)	2598952	3898456	3521496	3339635	0.85	35.79
TG 54:5*	C57H100O6	880.7482	13.61	ESI(+)	3133559	3200366	3234280	3189402	0.81	34.19
TG 54:5*	C57H100O6	880.7494	13.86	ESI(+)	3642915	3642178	3712100	3665731	0.93	39.26
TG 54:7*	C57H96O6	876.7191	13.86	ESI(+)	1712961	1668530	1741717	1707736	0.44	18.43
TG 54:7*	C57H96O6	876.7191	14.26	ESI(+)	964106	935727	1062520	987451	0.26	10.76
TG 56:3*	C59H108O6	912.8149	15.88	ESI(+)	1054463	1028759	1091160	1058127	0.27	11.52
TG 56:4*	C59H106O6	910.7986	14.99	ESI(+)	1501553	1572167	1507309	1527010	0.39	16.5
TG 56:4*	C59H106O6	910.7967	15.98	ESI(+)	505277	590497	628778	574851	0.15	6.37
TG 56:5*	C59H104O6	908.7837	14.35	ESI(+)	2656116	2576723	2631071	2621303	0.67	28.15
TG 8:0_8:0_10:0	C29H54O6	498.3925	6.26	ESI(+)	13500137	13059707	13298976	13286273	3.37	141.63
