# Supplementary material for: LiLA: lipid lung-based ATLAS built through a comprehensive workflow designed for an accurate lipid annotation
Source: Commun Biol. 2024 Jan 5;7:45. doi: 10.1038/s42003-023-05680-7 (PMC10770321; doi:10.1038/s42003-023-05680-7)
Supplement: Supplementary file 6 — Supplementary Data 4 [file 42003_2023_5680_MOESM6_ESM.pdf]

Supplementary Data 4. Lipid alterations detected after comparing *Mtb*$^+$*4w* vs *Mtb*$^-$ and *Mtb*$^+$*12w* vs *Mtb*$^-$. The table includes the lipid candidate, the formula, retention time (RT) in minutes, the ionization mode where the lipid species were detected, the percentage of change calculated within each comparison, and the univariate (Mann-Whitney U test p-value and Benjamini-Hochberg corrected p-value, pBH) and multivariate (variable influence on projection, VIP) statistical analysis results for each comparison.

					***Mtb*$^+$*4w* vs *Mtb*$^-$**				***Mtb*$^+$*12w* vs *Mtb*$^-$**			
**Lipid name**	**Formula**	**Mass**	**RT**	**ESI Mode**	**% change**	**p-value**	**pBH**	**VIP**	**% change**	**p-value**	**pBH**	**VIP**
BMP 16:0_16:0	C38H75O10P	722.5094	7.13	LC-ESI(-) MS	18.4	0.026	0.050	0.64	50.4	0.0043	0.013	1.14
BMP 16:0_20:4	C42H75O10P	770.5087	6.26	LC-ESI(-) MS	38.5	0.0022	0.0080	0.78	96.7	0.0043	0.013	1.22
BMP 22:6_22:6	C50H75O10P	866.5091	4.86	LC-ESI(-) MS	110.3	0.0022	0.0080	0.90	159.3	0.0043	0.013	1.05
Car 16:0	C23H45NO4	399.3354	2.38	LC-ESI(+) MS	74.1	0.015	0.056	1.26	67.2	0.082	0.12	0.82
Car 18:1	C25H47NO4	425.3512	2.58	LC-ESI(+) MS	80.9	0.0043	0.023	1.02	50.3	0.13	0.17	0.54
Cer 16:0;2O/18:1	C34H67NO3	537.5126	9.06	LC-ESI(-) MS	54.5	0.0022	0.0080	2.06	72.2	0.0043	0.013	2.32
Cer 18:0;2O/16:0	C34H69NO3	539.5274	10.08	LC-ESI(-) MS	112.6	0.0022	0.0080	1.05	196.6	0.0043	0.013	1.30
Cer 18:0;2O/24:0	C42H85NO3	651.6522	12.72	LC-ESI(-) MS	63.6	0.015	0.032	1.34	71.7	0.0087	0.023	1.35
Cer 18:0;2O/24:1	C42H83NO3	649.6359	12.39	LC-ESI(-) MS	101.6	0.0022	0.0080	1.63	152.2	0.0043	0.013	1.88
Cer 18:1;2O/16:0	C34H67NO3	537.5125	9.19	LC-ESI(-) MS	28.9	0.041	0.075	1.89	52.1	0.0087	0.023	2.38
Cer 18:1;2O/24:0	C42H83NO3	649.6384	12.58	LC-ESI(-) MS	19.4	0.041	0.075	2.90	31.6	0.0087	0.023	3.83
Cer 18:1;2O/24:1	C42H81NO3	647.6228	12.27	LC-ESI(-) MS	35.9	0.0022	0.0080	4.20	47.9	0.0043	0.013	4.72
Cer 18:1;2O/24:2	C42H79NO3	645.6061	12.04	LC-ESI(-) MS	28.5	0.015	0.032	2.41	24.3	0.13	0.19	2.22
Cer 18:2;2O/24:0	C42H81NO3	647.6224	12.27	LC-ESI(-) MS	36.0	0.0022	0.0080	4.21	48.0	0.0043	0.013	4.73
Cer 18:2;2O/24:1	C42H79NO3	645.6059	12.04	LC-ESI(-) MS	28.5	0.015	0.032	2.41	24.3	0.13	0.19	2.22
DG 14:0_18:1	C35H66O5	566.4914	11.48	LC-ESI(-) MS	15.1	0.24	0.32	0.47	67.2	0.0043	0.013	1.07
DG 16:0_16:0	C35H68O5	568.5079	11.93	LC-ESI(-) MS	45.5	0.0022	0.0080	1.49	65.0	0.0043	0.013	1.72
DG 16:0_16:1	C35H66O5	566.4914	11.48	LC-ESI(-) MS	15.1	0.24	0.32	0.47	67.2	0.0043	0.013	1.07
DG 16:0_18:1	C37H70O5	594.5232	11.96	LC-ESI(-) MS	-2.9	0.70	0.78	0.78	25.4	0.017	0.039	1.60
DG 16:0_20:3	C39H70O5	618.5235	11.73	LC-ESI(-) MS	-34.1	0.0022	0.0080	2.85	-38.5	0.0043	0.013	2.85
DG 16:0_20:4	C39H68O5	616.5086	11.55	LC-ESI(-) MS	-42.0	0.0022	0.0080	2.25	-2.6	0.93	0.94	0.85
DG 16:0_22:5	C41H70O5	642.5174	11.64	LC-ESI(-) MS	-45.2	0.0022	0.0080	2.66	-20.5	0.017	0.039	1.70
DG 16:1_16:0	C35H66O5	566.4914	11.48	LC-ESI(-) MS	15.1	0.24	0.32	0.47	67.2	0.0043	0.013	1.07
DG 18:0_20:4	C41H72O5	644.5403	11.98	LC-ESI(+) MS	-46.1	0.0022	0.017	2.41	-44.1	0.0043	0.010	1.56
DG 18:0_22:6	C43H72O5	668.5266	11.91	LC-ESI(-) MS	-39.4	0.0022	0.0080	1.73	-41.8	0.0043	0.013	1.72
DG 18:1_16:0	C37H70O5	594.5232	11.96	LC-ESI(-) MS	-2.9	0.70	0.78	0.78	25.4	0.017	0.039	1.60
DG 18:1_18:2	C39H70O5	618.5235	11.73	LC-ESI(-) MS	-34.1	0.0022	0.0080	2.85	-38.5	0.0043	0.013	2.85
DG 18:1_18:3	C39H68O5	616.5064	10.89	LC-ESI(-) MS	-41.9	0.0022	0.0080	1.35	-58.1	0.0043	0.013	1.52
DG 18:1_20:4	C41H70O5	642.5234	11.64	LC-ESI(+) MS	-39.9	0.0022	0.017	1.02	-23.4	0.0087	0.019	0.51
DG 18:1_22:4	C43H74O5	670.5518	12.00	LC-ESI(-) MS	-24.6	0.0022	0.0080	1.18	-21.8	0.052	0.092	0.91
DG 18:1_22:6	C43H70O5	666.5233	11.47	LC-ESI(-) MS	-29.4	0.0022	0.0080	1.28	-22.2	0.017	0.039	1.02
DG 18:2_18:1	C39H70O5	618.5227	11.74	LC-ESI(+) MS	-33.4	0.0022	0.017	1.07	-39.1	0.0043	0.010	0.77
DG 22:4_18:1	C43H74O5	670.5376	12.00	LC-ESI(-) MS	-30.8	0.0022	0.0080	1.41	-20.5	0.0087	0.023	1.12
FA 14:0	C14H28O2	228.2097	2.35	LC-ESI(-) MS	110.7	0.0022	0.0080	4.90	109.1	0.0043	0.013	4.53
FA 16:1	C16H30O2	254.2249	2.57	LC-ESI(-) MS	60.3	0.0022	0.0080	1.71	51.6	0.017	0.039	1.53
FA 18:1	C18H34O2	282.2567	3.47	LC-ESI(-) MS	21.1	0.026	0.050	2.53	3.5	0.66	0.74	0.62
FA 18:2	C18H32O2	280.2410	2.86	LC-ESI(-) MS	29.7	0.0022	0.0080	2.58	-22.0	0.017	0.039	1.90
FA 20:4	C20H32O2	304.2410	2.77	LC-ESI(-) MS	84.0	0.0022	0.0080	3.61	77.1	0.0087	0.023	3.23
FA 22:4	C22H36O2	332.2720	3.50	LC-ESI(-) MS	38.2	0.0022	0.0080	0.69	113.5	0.0043	0.013	1.12
FA 22:5	C22H34O2	330.2562	2.99	LC-ESI(-) MS	42.9	0.0022	0.0080	1.08	7.8	0.54	0.62	0.53
FA 22:6	C22H32O2	328.2408	2.58	LC-ESI(-) MS	39.5	0.0087	0.022	1.84	3.0	0.93	0.94	0.84
LPC 18:1/0:0	C26H52NO7P	521.3493	2.77	LC-ESI(+) MS	70.5	0.0087	0.038	1.05	-7.0	0.79	0.84	0.40
LPC 18:2/0:0	C26H50NO7P	519.3326	2.20	LC-ESI(+) MS	95.0	0.0022	0.017	1.46	-17.5	0.18	0.24	0.47
LPC 20:4/0:0	C28H50NO7P	543.3336	2.15	LC-ESI(+) MS	121.2	0.0022	0.017	1.23	20.1	0.43	0.52	0.39
MG 18:1	C21H40O4	356.2923	3.74	LC-ESI(-) MS	85.2	0.0022	0.0080	1.25	41.6	0.030	0.059	0.73
MG 20:2	C23H42O4	382.3092	2.38	LC-ESI(+) MS	73.2	0.015	0.056	1.26	66.4	0.082	0.12	0.82
MG 22:6	C25H38O4	402.2770	2.99	LC-ESI(-) MS	-19.7	0.093	0.15	0.87	-45.5	0.017	0.039	1.33
PC 13:0_22:6	C43H74NO8P	763.5179	7.86	LC-ESI(+) MS	37.6	0.0022	0.017	3.71	101.7	0.0043	0.010	4.06
PC 14:0_16:0	C38H76NO8P	705.5316	7.37	LC-ESI(+) MS	45.1	0.0043	0.023	3.96	92.1	0.0043	0.010	3.71
PC 15:0_16:0	C39H78NO8P	719.5465	8.24	LC-ESI(+) MS	24.4	0.0087	0.038	1.11	8.3	0.66	0.72	0.52
PC 16:0_14:0	C38H76NO8P	705.5314	7.37	LC-ESI(+) MS	45.2	0.0043	0.023	3.97	92.2	0.0043	0.010	3.71
PC 16:0_15:0	C39H78NO8P	719.5457	8.23	LC-ESI(+) MS	23.8	0.0087	0.038	1.10	8.3	0.54	0.61	0.52
PC 16:0_16:0	C40H80NO8P	733.5622	9.27	LC-ESI(+) MS	15.6	0.0087	0.038	6.42	38.2	0.0043	0.010	6.85
PC 16:0_16:1	C40H78NO8P	731.5469	7.64	LC-ESI(+) MS	39.4	0.0022	0.017	5.51	78.5	0.0043	0.010	5.11
PC 16:0_18:1	C42H82NO8P	759.5785	9.59	LC-ESI(+) MS	14.9	0.041	0.13	4.02	21.4	0.052	0.084	3.40
PC 16:0_18:2	C42H80NO8P	757.5625	8.08	LC-ESI(+) MS	13.8	0.041	0.13	3.50	4.0	0.66	0.72	2.03
PC 16:0_20:2	C44H84NO8P	785.5930	9.96	LC-ESI(+) MS	27.8	0.0043	0.023	1.13	54.9	0.0043	0.010	1.08
PC 16:0_20:4	C44H80NO8P	781.5627	7.86	LC-ESI(+) MS	41.5	0.0022	0.017	4.31	120.7	0.0043	0.010	4.87

PC 16:0_22:4	C46H84NO8P	809.5960	9.34	LC-ESI(+) MS	55.3	0.0043	0.023	1.52	150.1	0.0043	0.010	1.66
PC 16:0_22:5	C46H82NO8P	807.5785	8.07	LC-ESI(+) MS	47.7	0.0087	0.038	2.21	83.7	0.0043	0.010	1.93
PC 16:0_22:6	C46H80NO8P	805.5643	7.51	LC-ESI(+) MS	34.5	0.015	0.056	3.46	55.1	0.017	0.034	2.95
PC 16:1_16:0	C40H78NO8P	731.5470	7.64	LC-ESI(+) MS	39.5	0.0022	0.017	5.53	78.6	0.0043	0.010	5.11
PC 18:0_18:2	C44H84NO8P	785.5931	10.34	LC-ESI(+) MS	13.6	0.065	0.19	1.78	-23.7	0.0043	0.010	1.63
PC 18:0_20:4	C46H84NO8P	809.5932	10.00	LC-ESI(+) MS	30.6	0.0043	0.023	2.39	64.8	0.0043	0.010	2.35
PC 18:1/18:1	C44H84NO8P	785.5931	9.96	LC-ESI(+) MS	27.1	0.0043	0.023	1.10	55.1	0.0043	0.010	1.06
PC 18:1_16:0	C42H82NO8P	759.5782	9.60	LC-ESI(+) MS	15.1	0.041	0.13	4.04	21.7	0.052	0.084	3.41
PC 18:1_18:2	C44H82NO8P	783.5776	8.32	LC-ESI(+) MS	37.6	0.0022	0.017	1.58	25.4	0.13	0.17	0.86
PC 18:1_20:4	C46H82NO8P	807.5785	8.07	LC-ESI(+) MS	47.7	0.0087	0.038	2.21	83.7	0.0043	0.010	1.93
PC 18:2/14:0	C40H76NO8P	729.5312	6.54	LC-ESI(+) MS	125.9	0.0022	0.017	1.39	193.8	0.0043	0.010	1.16
PC 18:2/18:0	C44H84NO8P	785.5929	10.34	LC-ESI(+) MS	13.6	0.093	0.24	1.76	-24.2	0.0043	0.010	1.63
PC 18:2_16:0	C42H80NO8P	757.5625	8.08	LC-ESI(+) MS	13.7	0.041	0.13	3.50	4.0	0.66	0.72	2.03
PC 20:4_18:1	C46H82NO8P	807.5785	8.07	LC-ESI(+) MS	47.7	0.0087	0.038	2.21	83.7	0.0043	0.010	1.93
PC 22:6_16:0	C46H80NO8P	805.5645	7.51	LC-ESI(+) MS	34.4	0.015	0.056	3.46	55.0	0.017	0.034	2.95
PC 33:4	C41H74NO8P	739.5148	8.11	LC-ESI(-) MS	29.3	0.0043	0.013	0.86	88.9	0.0043	0.013	1.50
PC 34:3	C42H78NO8P	755.5460	6.76	LC-ESI(+) MS	68.9	0.0022	0.017	1.04	72.5	0.0043	0.010	0.68
PC 37:6	C45H78NO8P	791.5498	10.00	LC-ESI(+) MS	30.9	0.0043	0.023	2.28	54.6	0.0043	0.010	2.05
PC 37:7	C45H76NO8P	789.5357	8.06	LC-ESI(+) MS	48.3	0.0043	0.023	1.95	77.4	0.0087	0.019	1.65
PC O-32:0	C40H82NO7P	719.5830	10.99	LC-ESI(+) MS	84.6	0.0022	0.017	1.82	95.3	0.0043	0.010	1.25
PC O-36:4	C44H82NO7P	767.5832	9.14	LC-ESI(+) MS	98.3	0.0022	0.017	1.91	173.5	0.0043	0.010	1.65
PE 16:0_20:4	C41H74NO8P	739.5148	8.11	LC-ESI(-) MS	29.7	0.0043	0.013	0.86	90.8	0.0043	0.013	1.52
PE 16:0_22:5	C43H76NO8P	765.5322	8.33	LC-ESI(+) MS	32.0	0.0043	0.023	1.35	27.8	0.017	0.034	0.84
PE 16:0_22:6	C43H74NO8P	763.5179	7.86	LC-ESI(+) MS	37.7	0.0022	0.017	3.71	101.7	0.0043	0.010	4.06
PE 18:0_20:4	C43H78NO8P	767.5460	10.35	LC-ESI(-) MS	34.5	0.0043	0.013	1.38	58.1	0.13	0.19	1.75
PE 18:0_22:4	C45H82NO8P	795.5787	11.60	LC-ESI(-) MS	45.3	0.0022	0.008	0.70	121.1	0.0043	0.013	1.12
PE 18:0_22:6	C45H78NO8P	791.5462	9.83	LC-ESI(-) MS	33.2	0.015	0.032	1.08	38.1	0.030	0.060	1.21
PE 18:1_20:4	C43H76NO8P	765.5318	8.33	LC-ESI(+) MS	36.5	0.0043	0.023	1.41	32.2	0.017	0.034	0.89
PE 18:1_22:6	C45H76NO8P	789.5357	8.06	LC-ESI(+) MS	47.0	0.0043	0.023	1.94	76.6	0.0087	0.019	1.65
PE 18:1_O-16:1|PE 18:1_P-16:0	C39H76NO7P	701.5348	11.29	LC-ESI(-) MS	-31.5	0.0022	0.0080	1.50	-21.3	0.0043	0.013	1.22
PE 18:2_18:2	C41H74NO8P	739.5148	8.11	LC-ESI(-) MS	30.0	0.0043	0.013	0.86	91.4	0.0043	0.013	1.52
PE 22:6_16:0	C43H74NO8P	763.5178	7.85	LC-ESI(+) MS	30.5	0.0043	0.023	3.70	53.6	0.0043	0.010	4.06
PE 22:6_18:0	C45H78NO8P	791.5493	10.00	LC-ESI(+) MS	48.6	0.0043	0.023	2.27	77.8	0.0087	0.019	2.04
PE 40:6	C45H78NO8P	791.5497	10.00	LC-ESI(+) MS	11.3	0.48	0.76	2.28	30.2	0.030	0.053	2.05
PE 40:9	C45H72NO8P	785.4992	7.84	LC-ESI(+) MS	28.7	0.065	0.19	1.20	60.6	0.0087	0.019	1.32
PE O-16:1_18:1|PE P-16:0_18:1	C39H76NO7P	701.5348	11.29	LC-ESI(-) MS	-31.5	0.0022	0.0080	1.50	-21.3	0.0043	0.013	1.22
PE O-16:1_20:1|PE P-16:0_20:1	C41H80NO7P	729.5660	11.90	LC-ESI(-) MS	-34.5	0.0022	0.0080	1.28	-27.2	0.0043	0.013	1.11
PE O-16:1_20:4|PE P-16:0_20:4	C41H74NO7P	723.5203	9.12	LC-ESI(-) MS	22.1	0.065	0.11	1.40	75.8	0.0043	0.013	2.83
PE O-16:1_22:4|PE P-16:0_22:4	C43H78NO7P	751.5511	11.02	LC-ESI(-) MS	5.4	0.59	0.67	0.35	55.3	0.0087	0.023	1.03
PE O-18:1_18:1|PE P-18:0_18:1	C41H80NO7P	729.5652	11.90	LC-ESI(-) MS	-34.2	0.0022	0.0080	1.25	-27.0	0.0043	0.013	1.09
PE O-18:2_18:2|PE P-18:1_18:2	C41H76NO7P	725.5335	9.39	LC-ESI(+) MS	108.3	0.0022	0.017	1.32	80.6	0.0043	0.010	0.73
PG 16:0_16:0	C38H75O10P	722.5094	7.13	LC-ESI(-) MS	18.3	0.026	0.050	0.64	50.8	0.0043	0.013	1.14
PG 16:0_18:1	C40H77O10P	748.5255	7.25	LC-ESI(-) MS	9.0	0.39	0.49	0.75	63.3	0.0043	0.013	2.40
PG 16:0_20:4	C42H75O10P	770.5087	6.26	LC-ESI(-) MS	38.1	0.0022	0.0080	0.77	96.7	0.0043	0.013	1.22
PG 22:6_22:6	C50H75O10P	866.5092	4.86	LC-ESI(-) MS	110.3	0.0022	0.0080	0.90	159.3	0.0043	0.013	1.05
PI 16:0_20:4	C45H79O13P	858.5253	6.04	LC-ESI(-) MS	183.3	0.0022	0.0080	1.60	284.5	0.0043	0.013	1.89
PI 16:0_22:5	C47H81O13P	884.5403	6.17	LC-ESI(-) MS	203.8	0.0022	0.0080	1.24	292.4	0.0043	0.013	1.40
PI 16:0_22:6	C47H79O13P	882.5248	5.87	LC-ESI(-) MS	135.4	0.0022	0.0080	0.95	197.5	0.0043	0.013	1.09
PI 18:0_20:4	C47H83O13P	886.5573	7.27	LC-ESI(-) MS	135.2	0.0022	0.0080	4.06	145.0	0.052	0.092	3.78
PI 22:5_16:0	C47H81O13P	884.5403	6.17	LC-ESI(-) MS	203.6	0.0022	0.0080	1.24	292.6	0.0043	0.013	1.40
PS 18:0_18:2	C42H78NO10P	787.5279	7.86	LC-ESI(+) MS	37.0	0.0022	0.017	0.97	93.2	0.0043	0.010	1.08
PS 18:1_18:1	C42H78NO10P	787.5278	7.86	LC-ESI(+) MS	33.3	0.0022	0.017	1.00	84.4	0.0043	0.010	1.05
PS 21:2_20:4	C47H80NO10P	849.5484	10.33	LC-ESI(-) MS	29.5	0.0043	0.013	0.81	57.6	0.0043	0.013	1.13
PS 38:2	C44H82NO10P	815.5653	9.24	LC-ESI(-) MS	7.8	0.0043	0.013	0.97	17.8	0.0043	0.013	1.52
SM 18:0;2O/16:0	C39H81N2O6P	704.5828	7.95	LC-ESI(+) MS	60.3	0.0022	0.017	1.47	83.6	0.0043	0.010	1.13
SM 18:1;2O/16:0	C39H79N2O6P	702.5682	7.29	LC-ESI(+) MS	56.3	0.0022	0.017	4.50	52.8	0.0043	0.010	2.85
SM 18:1;2O/24:1	C47H93N2O6P	812.6788	11.98	LC-ESI(+) MS	21.1	0.015	0.056	1.98	26.6	0.030	0.053	1.51
SM 42:4;2O	C49H91N2O6P	834.6618	11.98	LC-ESI(+) MS	25.4	0.0022	0.017	1.15	29.8	0.0043	0.010	0.83
TG 14:0_18:1_20:1	C55H102O6	858.7685	14.82	LC-ESI(+) MS	-8.0	0.82	0.92	1.26	-43.3	0.0043	0.010	1.69
TG 16:0_16:0_18:2	C53H98O6	830.7371	14.27	LC-ESI(+) MS	-9.3	0.59	0.84	1.68	-54.0	0.0043	0.010	1.94
TG 16:0_16:0_18:3	C53H96O6	828.7213	13.79	LC-ESI(+) MS	-5.5	0.82	0.92	1.33	-59.7	0.0043	0.010	1.45
TG 16:0_16:0_20:3	C55H100O6	856.7528	14.28	LC-ESI(+) MS	-7.0	0.82	0.92	1.86	-65.6	0.0043	0.010	2.63
TG 16:0_16:1_18:1	C53H98O6	830.7371	14.27	LC-ESI(+) MS	-9.3	0.59	0.84	1.68	-54.0	0.0043	0.010	1.94
TG 16:0_16:1_18:2	C53H96O6	828.7211	13.78	LC-ESI(+) MS	-4.8	0.82	0.92	1.35	-60.2	0.0043	0.010	1.42
TG 16:0_16:1_20:3	C55H98O6	854.7370	13.83	LC-ESI(+) MS	-10.2	0.39	0.66	1.53	-71.1	0.0043	0.010	2.07
TG 16:0_18:0_18:2	C55H102O6	858.7685	14.82	LC-ESI(+) MS	-13.3	0.70	0.91	1.38	-44.1	0.0043	0.010	1.68
TG 16:0_18:1_16:1	C53H98O6	830.7371	14.27	LC-ESI(+) MS	-9.4	0.59	0.84	1.68	-53.1	0.0043	0.010	1.92
TG 16:0_18:1_18:1	C55H102O6	858.7685	14.82	LC-ESI(+) MS	-13.3	0.70	0.91	1.38	-44.2	0.0043	0.010	1.69
TG 16:0_18:1_18:2	C55H100O6	856.7528	14.28	LC-ESI(+) MS	-7.2	0.82	0.92	1.86	-65.6	0.0043	0.010	2.62
TG 16:0_18:1_18:3	C55H98O6	854.7370	13.83	LC-ESI(+) MS	-10.2	0.39	0.66	1.52	-71.7	0.0043	0.010	2.07
TG 16:0_18:2_16:0	C53H98O6	830.7371	14.27	LC-ESI(+) MS	-9.4	0.59	0.84	1.68	-53.9	0.0043	0.010	1.93

TG 16:0_18:2_18:0	C55H102O6	858.7685	14.82	LC-ESI(+) MS	-13.3	0.70	0.91	1.38	-44.0	0.0043	0.010	1.68
TG 16:0_18:2_18:1	C55H100O6	856.7528	14.28	LC-ESI(+) MS	-7.0	0.82	0.92	1.86	-65.5	0.0043	0.010	2.62
TG 16:0_18:2_18:2	C55H98O6	854.7369	13.83	LC-ESI(+) MS	-10.4	0.39	0.66	1.53	-71.5	0.0043	0.010	2.07
TG 16:0_18:2_22:6	C59H98O6	902.7346	13.84	LC-ESI(+) MS	-11.3	0.39	0.66	0.69	-54.2	0.0043	0.010	1.04
TG 16:0_18:3_16:0	C53H96O6	828.7210	13.78	LC-ESI(+) MS	-6.2	0.82	0.92	1.36	-61.9	0.0043	0.010	1.44
TG 16:0_18:3_18:0	C55H100O6	856.7528	14.28	LC-ESI(+) MS	-7.0	0.82	0.92	1.86	-65.5	0.0043	0.010	2.62
TG 16:0_18:3_18:1	C55H98O6	854.7369	13.83	LC-ESI(+) MS	-10.2	0.39	0.66	1.53	-71.4	0.0043	0.010	2.07
TG 16:0_20:2_16:1	C55H100O6	856.7528	14.28	LC-ESI(+) MS	-7.5	0.82	0.92	1.86	-65.6	0.0043	0.010	2.63
TG 16:0_20:3_16:0	C55H100O6	856.7528	14.28	LC-ESI(+) MS	-7.0	0.82	0.92	1.86	-65.6	0.0043	0.010	2.63
TG 16:0_20:3_16:1	C55H98O6	854.7370	13.83	LC-ESI(+) MS	-10.2	0.39	0.66	1.53	-71.5	0.0043	0.010	2.07
TG 16:1_16:0_18:2	C53H96O6	828.7210	13.78	LC-ESI(+) MS	-5.0	0.82	0.92	1.35	-62.0	0.0043	0.010	1.44
TG 16:1_16:1_18:1	C53H96O6	828.7210	13.78	LC-ESI(+) MS	-5.2	0.82	0.92	1.35	-60.7	0.0043	0.010	1.43
TG 16:1_18:1_16:1	C53H96O6	828.7211	13.78	LC-ESI(+) MS	-5.6	0.82	0.92	1.35	-60.7	0.0043	0.010	1.43
TG 16:1_18:1_18:2	C55H98O6	854.7369	13.83	LC-ESI(+) MS	-10.3	0.39	0.66	1.53	-71.2	0.0043	0.010	2.07
TG 18:0_16:1_18:2	C55H100O6	856.7528	14.28	LC-ESI(+) MS	-8.1	0.70	0.91	1.86	-63.9	0.0043	0.010	2.62
TG 18:1/18:1/14:0	C53H98O6	830.7371	14.27	LC-ESI(+) MS	-9.2	0.59	0.84	1.68	-53.9	0.0043	0.010	1.93
TG 18:1/18:1/16:0	C55H102O6	858.7685	14.82	LC-ESI(+) MS	-13.3	0.70	0.91	1.38	-44.2	0.0043	0.010	1.68
TG 18:1/18:1/16:1	C55H100O6	856.7528	14.28	LC-ESI(+) MS	-7.1	0.82	0.92	1.86	-65.6	0.0043	0.010	2.63
TG 18:1_14:0_18:1	C53H98O6	830.7371	14.27	LC-ESI(+) MS	-9.4	0.59	0.84	1.68	-53.9	0.0043	0.010	1.93
TG 18:1_14:0_18:2	C53H96O6	828.7210	13.78	LC-ESI(+) MS	-6.2	0.82	0.92	1.36	-61.9	0.0043	0.010	1.44
TG 18:1_14:0_22:5	C57H98O6	878.7365	13.47	LC-ESI(+) MS	-15.1	0.31	0.60	0.76	-58.4	0.0043	0.010	1.03
TG 18:1_16:0_18:1	C55H102O6	858.7685	14.82	LC-ESI(+) MS	-13.3	0.70	0.91	1.38	-44.3	0.0043	0.010	1.69
TG 18:1_16:0_18:2	C55H100O6	856.7528	14.28	LC-ESI(+) MS	-6.6	0.82	0.92	1.85	-65.4	0.0043	0.010	2.61
TG 18:1_16:0_20:2	C57H104O6	884.7843	14.82	LC-ESI(+) MS	-5.7	0.48	0.76	1.01	-34.3	0.0043	0.010	1.59
TG 18:1_16:0_20:3	C57H102O6	882.7686	14.28	LC-ESI(+) MS	-8.8	0.70	0.91	1.30	-59.7	0.0043	0.010	2.00
TG 18:1_16:1_16:0	C53H98O6	830.7371	14.27	LC-ESI(+) MS	-9.2	0.59	0.84	1.68	-54.0	0.0043	0.010	1.93
TG 18:1_16:1_18:0	C55H102O6	858.7685	14.82	LC-ESI(+) MS	-7.9	0.82	0.92	1.26	-43.4	0.0043	0.010	1.69
TG 18:1_16:1_18:1	C55H100O6	856.7528	14.28	LC-ESI(+) MS	-6.8	0.82	0.92	1.85	-65.4	0.0043	0.010	2.62
TG 18:1_16:2_16:0	C53H96O6	828.7210	13.78	LC-ESI(+) MS	-6.0	0.82	0.92	1.36	-60.5	0.0043	0.010	1.43
TG 18:1_16:2_18:1	C55H98O6	854.7369	13.83	LC-ESI(+) MS	-10.2	0.39	0.66	1.52	-71.6	0.0043	0.010	2.07
TG 18:1_18:0_18:2	C57H104O6	884.7843	14.82	LC-ESI(+) MS	-5.6	0.48	0.76	1.00	-34.3	0.0043	0.010	1.59
TG 18:1_18:0_18:3	C57H102O6	882.7686	14.28	LC-ESI(+) MS	-8.8	0.70	0.91	1.30	-59.7	0.0043	0.010	2.00
TG 18:1_18:1_18:1	C57H104O6	884.7843	14.82	LC-ESI(+) MS	-5.6	0.48	0.76	1.00	-34.3	0.0043	0.010	1.59
TG 18:1_18:1_18:2	C57H102O6	882.7686	14.28	LC-ESI(+) MS	-8.7	0.70	0.91	1.30	-59.7	0.0043	0.010	2.00
TG 18:1_18:1_18:3	C57H100O6	880.7527	13.84	LC-ESI(+) MS	-12.4	0.59	0.84	1.17	-62.4	0.0043	0.010	1.70
TG 18:1_18:1_18:4	C57H98O6	878.7365	13.47	LC-ESI(+) MS	-14.6	0.39	0.66	0.75	-57.9	0.0043	0.010	1.03
TG 18:1_18:2_16:0	C55H100O6	856.7528	14.28	LC-ESI(+) MS	-6.8	0.82	0.92	1.85	-65.4	0.0043	0.010	2.62
TG 18:1_18:2_18:0	C57H104O6	884.7843	14.82	LC-ESI(+) MS	-5.5	0.48	0.76	1.00	-34.3	0.0043	0.010	1.58
TG 18:1_18:2_18:1	C57H102O6	882.7686	14.28	LC-ESI(+) MS	-8.7	0.70	0.91	1.30	-59.7	0.0043	0.010	2.00
TG 18:1_18:2_18:2	C57H100O6	880.7527	13.84	LC-ESI(+) MS	-12.1	0.59	0.84	1.16	-62.5	0.0043	0.010	1.70
TG 18:1_18:2_18:3	C57H98O6	878.7365	13.47	LC-ESI(+) MS	-14.2	0.39	0.66	0.75	-57.5	0.0043	0.010	1.03
TG 18:1_18:4_18:1	C57H98O6	878.7365	13.47	LC-ESI(+) MS	-14.4	0.39	0.66	0.75	-58.3	0.0043	0.010	1.02
TG 18:1_20:3_16:0	C57H102O6	882.7686	14.28	LC-ESI(+) MS	-8.8	0.70	0.91	1.30	-59.7	0.0043	0.010	2.00
TG 18:2/18:2/16:0	C55H98O6	854.7369	13.83	LC-ESI(+) MS	-10.2	0.39	0.66	1.52	-71.7	0.0043	0.010	2.07
TG 18:2/18:2/18:0	C57H102O6	882.7686	14.28	LC-ESI(+) MS	-8.9	0.70	0.91	1.30	-59.8	0.0043	0.010	2.00
TG 18:2/18:2/18:1	C57H100O6	880.7527	13.84	LC-ESI(+) MS	-12.1	0.59	0.84	1.17	-61.5	0.0043	0.010	1.70
TG 18:2_14:0_18:1	C53H96O6	828.7211	13.78	LC-ESI(+) MS	-5.6	0.82	0.92	1.35	-60.7	0.0043	0.010	1.43
TG 18:2_15:0_18:1	C54H98O6	842.7306	13.77	LC-ESI(+) MS	-9.7	0.94	0.96	1.49	-42.4	0.017	0.034	1.41
TG 18:2_16:0_16:0	C53H98O6	830.7371	14.27	LC-ESI(+) MS	-9.4	0.59	0.84	1.68	-53.1	0.0043	0.010	1.92
TG 18:2_16:0_16:1	C53H96O6	828.7211	13.78	LC-ESI(+) MS	-5.6	0.82	0.92	1.35	-60.7	0.0043	0.010	1.43
TG 18:2_16:0_17:1	C54H98O6	842.7313	13.77	LC-ESI(+) MS	-9.6	0.82	0.92	1.52	-43.1	0.017	0.034	1.46
TG 18:2_16:0_18:1	C55H100O6	856.7528	14.28	LC-ESI(+) MS	-7.2	0.82	0.92	1.86	-65.6	0.0043	0.010	2.62
TG 18:2_16:0_18:2	C55H98O6	854.7369	13.83	LC-ESI(+) MS	-10.2	0.39	0.66	1.52	-71.5	0.0043	0.010	2.07
TG 18:2_16:0_20:1	C57H104O6	884.7843	14.82	LC-ESI(+) MS	-5.6	0.48	0.76	1.00	-34.4	0.0043	0.010	1.58
TG 18:2_16:1_18:1	C55H98O6	854.7369	13.83	LC-ESI(+) MS	-10.2	0.39	0.66	1.52	-71.6	0.0043	0.010	2.07
TG 18:2_18:0_14:0	C53H98O6	830.7371	14.27	LC-ESI(+) MS	-9.2	0.59	0.84	1.68	-54.0	0.0043	0.010	1.93
TG 18:2_18:0_18:2	C57H102O6	882.7686	14.28	LC-ESI(+) MS	-8.8	0.70	0.91	1.30	-59.7	0.0043	0.010	2.00
TG 18:2_18:0_18:3	C57H100O6	880.7527	13.84	LC-ESI(+) MS	-12.4	0.59	0.84	1.17	-62.5	0.0043	0.010	1.70
TG 18:2_18:1_14:0	C53H96O6	828.7210	13.78	LC-ESI(+) MS	-6.0	0.82	0.92	1.36	-60.5	0.0043	0.010	1.43
TG 18:2_18:1_16:1	C55H98O6	854.7369	13.83	LC-ESI(+) MS	-10.2	0.39	0.66	1.52	-71.6	0.0043	0.010	2.07
TG 18:2_18:1_18:2	C57H100O6	880.7527	13.84	LC-ESI(+) MS	-12.1	0.59	0.84	1.16	-61.9	0.0043	0.010	1.70
TG 18:2_18:2_18:2	C57H98O6	878.7365	13.47	LC-ESI(+) MS	-14.9	0.31	0.60	0.77	-57.3	0.0043	0.010	1.03
TG 18:2_18:3_18:0	C57H100O6	880.7527	13.84	LC-ESI(+) MS	-12.2	0.59	0.84	1.16	-62.5	0.0043	0.010	1.70
TG 18:2_18:3_18:1	C57H98O6	878.7365	13.47	LC-ESI(+) MS	-14.8	0.31	0.60	0.76	-57.8	0.0043	0.010	1.03
TG 18:2_20:2_16:0	C57H102O6	882.7686	14.28	LC-ESI(+) MS	-8.7	0.70	0.91	1.30	-59.7	0.0043	0.010	2.00
TG 18:3_16:0_20:3	C57H98O6	878.7365	13.47	LC-ESI(+) MS	-14.5	0.39	0.66	0.76	-57.2	0.0043	0.010	1.03
TG 18:3_18:1_18:2	C57H98O6	878.7365	13.47	LC-ESI(+) MS	-14.8	0.31	0.60	0.77	-57.2	0.0043	0.010	1.03
TG 18:3_22:3_14:0	C57H98O6	878.7365	13.47	LC-ESI(+) MS	-15.2	0.31	0.60	0.77	-58.1	0.0043	0.010	1.03
